# Responses of transcriptome and metabolome in the roots of *Pugionium cornutum* (L.) Gaertn to exogenously applied phthalic acid

**DOI:** 10.1186/s12870-022-03927-9

**Published:** 2022-11-17

**Authors:** Xiaoyan Zhang, Kezhen Ning, Zhongren Yang, Xiumei Huang, Hongtao Yu, Nana Fu, Xinyuan Qin, Lizhen Hao, Fenglan Zhang

**Affiliations:** 1 College of Horticultural and Plant Protection, Inner Mongolia Agricultural University, Inner Mongolia Key Laboratory of Wild Peculiar Vegetable Germplasm Resource and Germplasm Enhancement, Hohhot, 010011 China; 2 Inner Mongolia Autonomous Region Key Laboratory of Big Data Research and Application for Agriculture and Animal Husbandry, Hohhot, 010011 China

**Keywords:** RNA-seq, Metabolites, *Pugionium cornutum* (L.) Gaertn., Autotoxicity, Phthalic acid

## Abstract

**Background:**

The yield and quality of *Pugionium cornutum* (L.) Gaertn., a healthy, green vegetable with low sugar and high protein contents and high medicinal value, is severely affected by autotoxicity, which is a leading factor in the formation of plant disease. To help characterize the autotoxicity mechanism of *P. cornutum* (L.) Gaertn., we performed transcriptomic and metabolic analysis of the roots of *P. cornutum* (L.) Gaertn. response to phthalic acid, an autotoxin from *P. cornutum* (L.) Gaertn.

**Results:**

In this study, high-throughput sequencing of nine RNA-seq libraries generated from the roots.of *P. cornutum* (L.) Gaertn. under different phthalic acid treatments yielded 37,737 unigenes. In total, 1085 (703 upregulated and 382 downregulated) and 5998 (4385 upregulated and 1613 downregulated) DEGs were identified under 0.1 and 10 mmol·L^− 1^ phthalic acid treatment, respectively, compared with the control treatment. Glutathione metabolism was among the top five important enriched pathways. In total, 457 and 435 differentially accumulated metabolites were detected under 0.1 and 10 mmol·L^− 1^ phthalic acid treatment compared with the control, respectively, of which 223 and 253, respectively, increased in abundance. With the increase in phthalic acid concentration, the accumulation of ten metabolites increased significantly, while that of four metabolites decreased significantly, and phthalic acid, dambonitol, 4-hydroxy-butyric acid, homocitrulline, and ethyl β-D-glucopyranoside were 100 times more abundant under the 10 mmol·L^− 1^ phthalic acid treatment than under the control. Seventeen differentially expressed genes significantly associated with phthalic acid content were identified. In addition, the L-histidinol content was highest under 0.1 mmol·L^− 1^ phthalic acid, and a total of eleven differentially expressed genes were significantly positively correlated with the L-histidinol content, all of which were annotated to heat shock proteins, aquaporins and cysteine proteases.

**Conclusions:**

Accumulation of autotoxins altered the metabolic balance in *P. cornutum* (L.) Gaertn. and influenced water absorption and carbon and nitrogen metabolism. These important results provide insights into the formation mechanisms of autotoxicity and for the subsequent development of new control measures to improve the production and quality of replanted plants.

**Supplementary Information:**

The online version contains supplementary material available at 10.1186/s12870-022-03927-9.

## Background

*Pugionium cornutum* (L.) Gaertn., *Brassicaceae*, is an endemic biennial herbaceous species from the sandy region of the Mongolian Plateau in central Asia and is found throughout the mobile dunes of Horqin, Hunshandake, Kubuqi (eastern), Mu Us and Ningxia in China [1]. This species has a well-developed root system and is extremely resistant to sand cover, which can effectively prevent sand dunes from moving forwards; thus, this species is excellent for sand fixation and soil erosion control. *P. cornutum* (L.) Gaertn. is also a healthy, green vegetable species with low sugar and high protein contents and high medicinal value [2, 3]. However, the numbers of individuals and the distribution range of population have been decreasing, and the species is currently endangered. After years of resource investigation and research, it has been found that one- and two-year-old wild plants of *P. cornutum* (L.) Gaertn. cannot coexist, and where members of the same wild population do exist, the plants grow continuously for no more than 2 years. In addition, productivity and quality significantly decline when *P. cornutum* (L.) Gaertn. is planted in the field for several years; this decline is commonly described as “replant disease” or “the consecutive monoculture problem”. This problem is not restricted to *P. cornutum* (L.) Gaertn. but has been found for various medicinal, vegetable and horticultural plant species [4]. It has been suggested that autotoxicity is the most frequent cause of replanting disease [5, 6].

Autotoxicity, one of many diverse abiotic stresses, leads to poor crop growth, frequent occurrence of pests and diseases, poor quality and reduced yields, and it has been confirmed in a number of plant species, including crop and weed species [7–12]. Autotoxic substances are the key to the production of autotoxicity, and root exudates and residues in plants are the main ways to produce autotoxic substances; thus, autotoxic substances that accumulate in soil are the most important factors leading to the formation of replanting disease and promoting the transformation of microbial communities [13]. Several groups of compounds, such as terpenoids and steroids, phenols, coumarins, flavonoids, tannins, and alkaloids, have been shown to be autotoxins. The phytotoxicity of phenolic compounds has been extensively researched [14, 15]. Autotoxins severely damage many physiological and biochemical processes of plants, even irreversibly. For instance, they result in membrane lipid peroxidation and impair the structure and function of the entire cell membrane by reactive oxygen species (ROS) accumulation and inhibiting the antioxidant systems of plants [16–20]; limit the ability of the plants to absorb essential ions, solutes and water by inhibiting membrane H^+^-ATPase activity [21–23]; and significantly affect respiration by disrupting oxidative phosphorylation, the normal function of mitochondria, and the adenosine triphosphate (ATP) synthase activity of plants [23, 24].

Phthalic acid is a principal autotoxin from root exudates and residues of *P. cornutum* (L.) Gaertn.. Members of our research group have treated *P. cornutum* (L.) Gaertn. seedlings with phthalic acid, and the results are as follows: the activity of antioxidant enzymes is disrupted, the structure and function of the cell membrane are destroyed, the photosynthetic rate is reduced, the operation of the photosynthetic system is affected, and its carbon assimilation operation and organic matter accumulation are further hindered in *P. cornutum* (L.) Gaertn. seedlings [25, 26]. However, the mechanism of how *P. cornutum* (L.) Gaertn. responds to autotoxins at the molecular and metabolic levels remains largely unknown. In this study, we constructed RNA sequencing (RNA-seq) libraries from RNA extracted from roots treated with phthalic acid. We identified enriched functional terms in response to this stress and used LC–MS technology to analyse the changes in metabolite accumulation. These results provide a comprehensive understanding of the *P. cornutum* (L.) Gaertn. response to phthalic acid stress and lay a foundation for improving the resistance or endurance of *P. cornutum* (L.) Gaertn. to autotoxins present in the environment.

## Methods

### Design of the experiment

The experimental site for this study was in the Intelligent Greenhouse of the Key Laboratory of Germplasm Resources and Germplasm Innovation of Wild Endemic Vegetables, Inner Mongolia Agricultural University, Hohhot, Inner Mongolia Autonomous Region, China. No specific permissions were required for this experiment or its related activities. Fenglan Zhang formally identified the plant material used in our study, and a voucher specimen of *P. cornutum* (L.) Gaertn. has been deposited in our laboratory. Fruits of wild *P. cornutum* (L.) Gaertn. were collected from Mu Us Sandy Land, Ordos city. After the fruits were allowed to naturally dried and stored for 6 months, the peels were removed, and the mature, undamaged and fully developed seeds were selected for testing. The sterilized seeds were placed in a dark intelligent lighting incubator at 28 °C for germination, and the seeds displaying consistent germination were sown in plastic pots (18 cm in height, 15 cm in diameter) filled with a mixed substrate of vermiculite and soil (*V:V*, 1:1). When the seedlings had developed fully expanded cotyledons, treatments were applied. The plants in the control (CK) group were watered with distilled water, and those in the treatment groups were treated with 0.1 and 10 mmol·L^− 1^ phthalic acid solution once every other 3 days (100 mL·pot^− 1^) each time. When the seedlings reached the six-leaf stage and produced one shoot, the roots were harvested for transcriptome and metabolome analyses. Each treatment was replicated four times, and the experiment for transcriptome sequencing was repeated three times with six biological replicates for metabolome analyses. All the methods, including those for the plant experimental research, were performed in accordance with relevant institutional, national, and international guidelines and legislation.

### Total RNA extraction and cDNA library construction

Total RNA was extracted using a mirVana miRNA Isolation Kit (Ambion) following the manufacturer’s protocol. RNA integrity was evaluated using an Agilent 2100 Bioanalyzer (Agilent Technologies, Santa Clara, CA, USA). The samples with an RNA integrity number (RIN) ≥ 7 were subjected to subsequent analysis. The libraries were constructed using a TruSeq Stranded mRNA LT Sample Prep Kit (Illumina, San Diego, CA, USA) according to the manufacturer’s instructions. Then, the nine libraries were sequenced on the Illumina sequencing platform (HiSeq™ 2500), and 150 bp paired-end reads were generated. The RNA-seq experimental process is shown in Fig. S1. (Refer to the technical documents of OE Biotech RNA-seq.)

### Sample preparation for LC–MS

The processing of the samples is shown in Fig. S2. Eighty mg accurately weighed sample was transferred to a 1.5 mL Eppendorf tube. Two small steel balls were added to the tube. Twenty μL of 2-chloro-l-phenylalanine (0.3 mg·mL^− 1^) dissolved in methanol as internal standard and 1 mL mixture of methanol and water (*V:V*, 7:3) were added to each sample, samples were placed at − 20 °C for 2 min. Then grinded at 60 HZ for 2 min, and ultrasonicated at ambient temperature for 30 min after vortexed, then placed at − 20 °C for night. Samples were centrifuged at 13000 rpm, 4 °C for 10 min. The supernatants (150 μL) from each tube were collected using crystal syringes, filtered through 0.22 μm microfilters and transferred to LC vials. The vials were stored at − 80 °C until LC -MS analysis. Quality control (QC) samples were prepared by mixing aliquots of the all samples to be a pooled sample, and the volume of the QC samples was the same as that of the samples (Methodology provided by Shanghai Lu-Ming Biotech Co., Ltd., Shanghai, China).

An LC–MS system consisting of an AB ExionLC ultrahigh-performance LC instrument in tandem with an AB TripleTOF 6600 plus high-resolution mass spectrometer was used to analyse the metabolic spectrum in positive and negative electrospray ionization (ESI) modes. The chromatography conditions were as follows: column, ACQUITY UPLC HSS T3 (100 mm × 2.1 mm, 1.8 μm); column temperature, 45 °C; mobile phase A, water (containing 0.1% formic acid); mobile phase B, acetonitrile (containing 0.1% formic acid); flow rate, 0.35 mL·min^− 1^; and injection volume, 2 μL.

### Bioinformatic analysis

#### QC and de novo assembly

Transcriptome sequencing and analysis were conducted by staff at OE Biotech Co., Ltd. (Shanghai, China). The raw data (raw reads) were processed using Trimmomatic [27]. The reads containing poly-N sequences and the low-quality reads were removed to obtain clean reads. After removing adaptor and low-quality sequences, the clean reads were assembled into expressed sequence tag clusters (contigs) and de novo assembled into transcripts by using Trinity (version 2.4) [28] with the paired-end method. The longest transcript was chosen as a unigene based on the similarity and length of a sequence for subsequent analysis.

### Unigene quantification, analysis of differentially expressed genes (DEGs), cluster analysis, and Kyoto encyclopedia of genes and genomes (KEGG) enrichment

Fragments per kilobase of transcript per million mapped reads (FPKM) [29] and the read count value of each unigene was calculated using Bowtie2 [30] and eXpress [31]. DEGs were identified using the DESeq [32] functions estimate Size Factors and nbinom Test. A *p*-value < 0.05 and fold-change (FC) > 2 or fold-change < 0.5 were set as the thresholds for significantly differential expression. Hierarchical cluster analysis of the DEGs was performed to explore transcript expression patterns. KEGG pathway enrichment analysis of the DEGs based on the hypergeometric distribution was performed using R.

### Data preprocessing and statistical analysis of metabolite

The acquired LC–MS raw data were analysed by progenesis QI software (Waters Corporation, Milford, USA). The metabolites were identified by progenesis QI (Waters Corporation, Milford, USA) data processing software based on the information within public databases such as http://www.hmdb.ca/;http://www.lipidmaps.org/and self-built databases. The positive and negative data were combined to obtain combined data, which were imported into the R ropls package. Principal component analysis (PCA) and (orthogonal) partial least-squares-discriminant analysis (O) PLS-DA were carried out to visualize the metabolic alterations among experimental groups after mean centring (Ctr) and Pareto variance (Par) scaling, respectively. In this study, the default 7-round cross-validation was applied with 1/seventh of the samples being excluded from the mathematical model in each round to guard against overfitting.

The differentially accumulated metabolites (DAMs) were selected on the basis of the combination of a statistically significant threshold of variable influence on projection (VIP) values obtained from the OPLS-DA model and *p*-values from a two-tailed Student’s t test applied to the normalized peak areas, where metabolites with VIP values greater than 1.0 and *p*-values less than 0.05 were considered differentially accumulated.

### qRT–PCR analysis

To experimentally validate the transcript abundance results from the sequencing and expression profiles, ten unigenes were randomly selected for qRT–PCR analysis using a Power SYBR® Premix Ex Taq™ II Kit (TaKaRa). The reaction conditions were as follows: holding at 95 °C for 10 min; 40 cycles of 95 °C for 15 s and 60 °C for 5 min; and, then, a dissociation step at 94 °C for 90 s, 45 °C for 3 min and 94 °C for 10 s. β-actin, referring to R1_Unigene_BMK.22570, was used as a control for normalization [2]. The relative expression levels of selected unigenes were calculated using the 2^-△△Ct^ method for standardized analysis [33]. Three technical replicates were included for each sample. Primers were generated using Primer 5 (Supplementary Table S1).

## Results

### Analysis of the transcriptome dataset and unigene assembly

The CK and treatment groups of the roots of *P. cornutum* (L.) Gaertn. seedlings treated with phthalic acid in a total of three samples were sequenced in our study, with each sample including three biological replicates (CK_1, CK_2, CK_3, T1_1, T1_2, T1_3, T2_1, T2_2, T2_3). RNA-seq of the nine libraries yielded approximately 46.8 million reads. Quality filtration for low-quality reads (Q < 20) and contaminants was performed. The total raw and clean reads in each sample ranged from 40.02 Mb to 51.61 Mb and from 39.52 Mb to 51.17 Mb, respectively (Supplementary Table S2). A total of 60.39 Gb of clean data was obtained. The Q30 was higher than 95%, and GC contents were, on average, 45.45%, which indicated that the transcriptome results were reliable and suitable for subsequent analysis (Supplementary Table S2). The clean reads were mapped using Bowtie2, yielding a transcriptome comprising 37,737 unigenes. The trend of gene expression verified by qRT–PCR was similar to that determined by RNA-seq (Fig. S3), indicating that the data obtained by transcriptome analysis were highly reliable.

### DEGs of *P. cornutum* (L.) Gaertn. Seedlings treated with phthalic acid

**Fig. 1:**
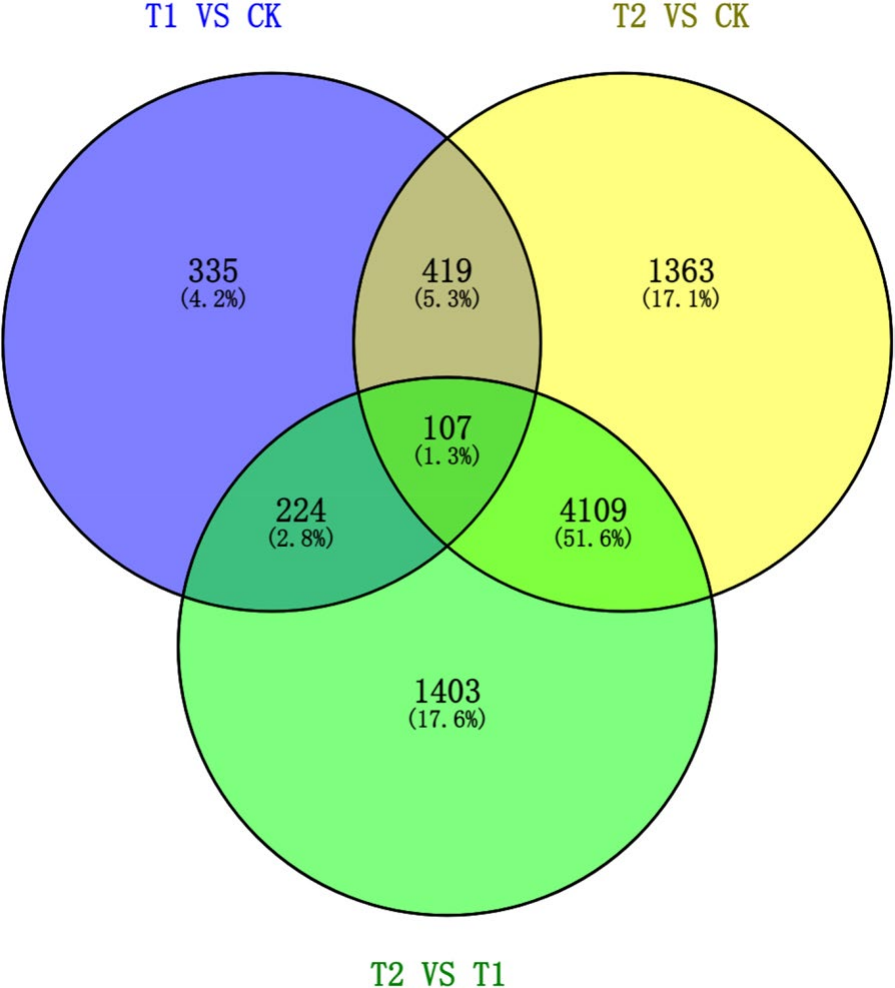
Venn diagram of DEGs. Note: The number 335 represents the number of DEGs detected only in T1 VS CK; 1363 represents the number of DEGs detected only in T2 VS CK; 1403 represents the number of DEGs detected only in T2 VS T1; (419 + 107) represents the number of DEGs shared between T1 VS CK and T2 VS CK; (224 + 107) represents the number of DEGs shared between T1 VS CK and T2 VS T1; and (4109 + 107) represents the number of DEGs shared between T2 VS CK and T2 VS T1

To identify DEGs among CK, T1, and T2, a transcriptomic comparison was carried out. The PCA score and results of the constructed heatmap proved the validity of the data (Supplementary Figs. S4 and S5). The samples could be separated into two clusters. Cluster I included T2, while T1 and CK were similar in cluster II according to the clustering analysis (Fig. S5). Based on the criteria of |log_2_FC| >1 and *p-*value < 0.05, 1085 (703 upregulated and 382 downregulated) and 5998 (4385 upregulated and 1613 downregulated) DEGs were identified in T1 and T2 compared with the CK, respectively. A total of 5843 DEGs were found in T2 VS T1, and 4023 of them were significantly upregulated. Interestingly, 107 (1.3%) DEGs were common across T1 VS CK, T2 VS CK, and T2 VS T1 (Fig. 1).

**Fig. 2:**
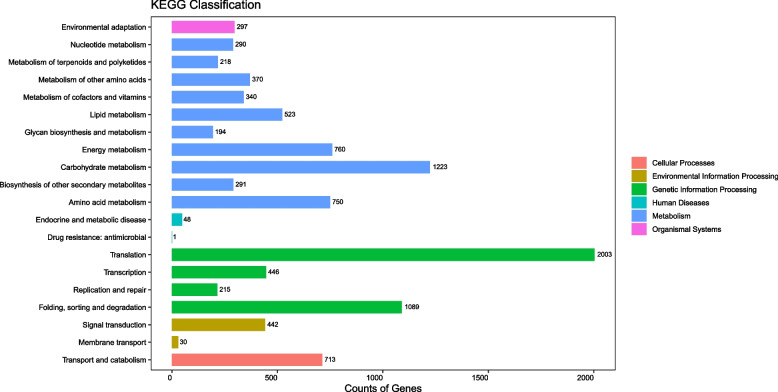
KEGG enriched pathways of DEGs detected in *P. cornutum* (L.) Gaertn. seedlings treated with phthalic acid

Furthermore, according to the KEGG database, 12,479 unigenes were assigned to 129 metabolic pathways, and translation (2003 unigenes), carbohydrate metabolism in metabolism (1223), folding, sorting and degradation (1089), energy metabolism (760), and amino acid metabolism (750) were the top 5 pathways (Fig. 2). All the DEGs were mapped to the KEGG database, and 275 and 2128 DEGs were annotated and assigned to 77 and 118 metabolic pathways based on the comparisons of T1 VS CK and T2 VS CK, respectively. In T1, the most important pathways of enrichment were protein processing in the endoplasmic reticulum (ko04141), phenylpropanoid biosynthesis (ko00940), glutathione metabolism (ko00480), anthocyanin biosynthesis (ko00942), and flavonoid biosynthesis (ko00941) (Supplementary Table S3). In T2, however, glutathione metabolism (ko00480); glucosinolate biosynthesis (ko00966); phenylalanine metabolism (ko00360); alanine, aspartate and glutamate metabolism (ko00250); and tyrosine metabolism (ko00350) were the most important pathways of enrichment (Supplementary Table S4). Together, these results provide important insights into how *P. cornutum* (L.) Gaertn. seedlings respond to low or high concentrations of phthalic acid.

### Untargeted metabolic profiling of *P. cornutum* (L.) Gaertn. Seedlings treated with phthalic acid

To comprehensively identify the metabolites in the roots of *P. cornutum* (L.) Gaertn. seedlings, an untargeted metabolic profiling was performed via LC–MS on three groups of plants treated with phthalic acid, which yielded 3841 ion features in the ESI (+) mode and 1294 ion features in the ESI (−) mode.

**Fig. 3:**
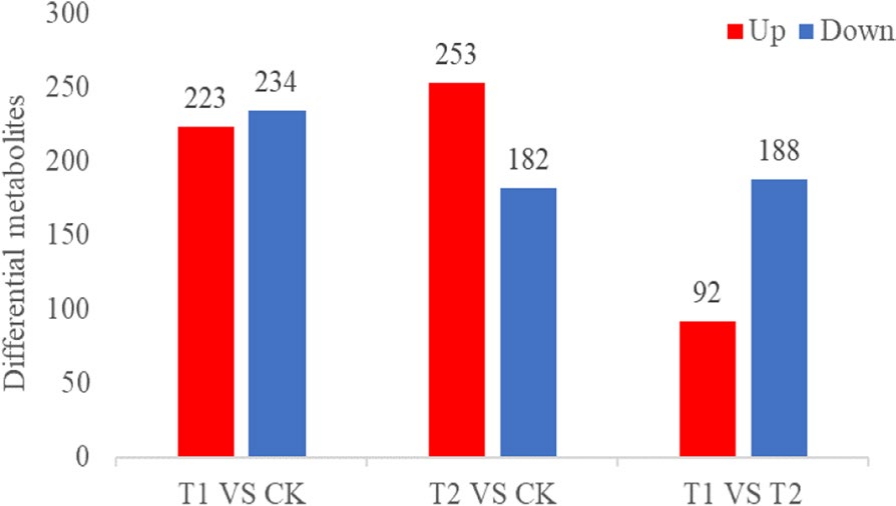
Number of DAMs after treatment with phthalic acid

**Fig. 4:**
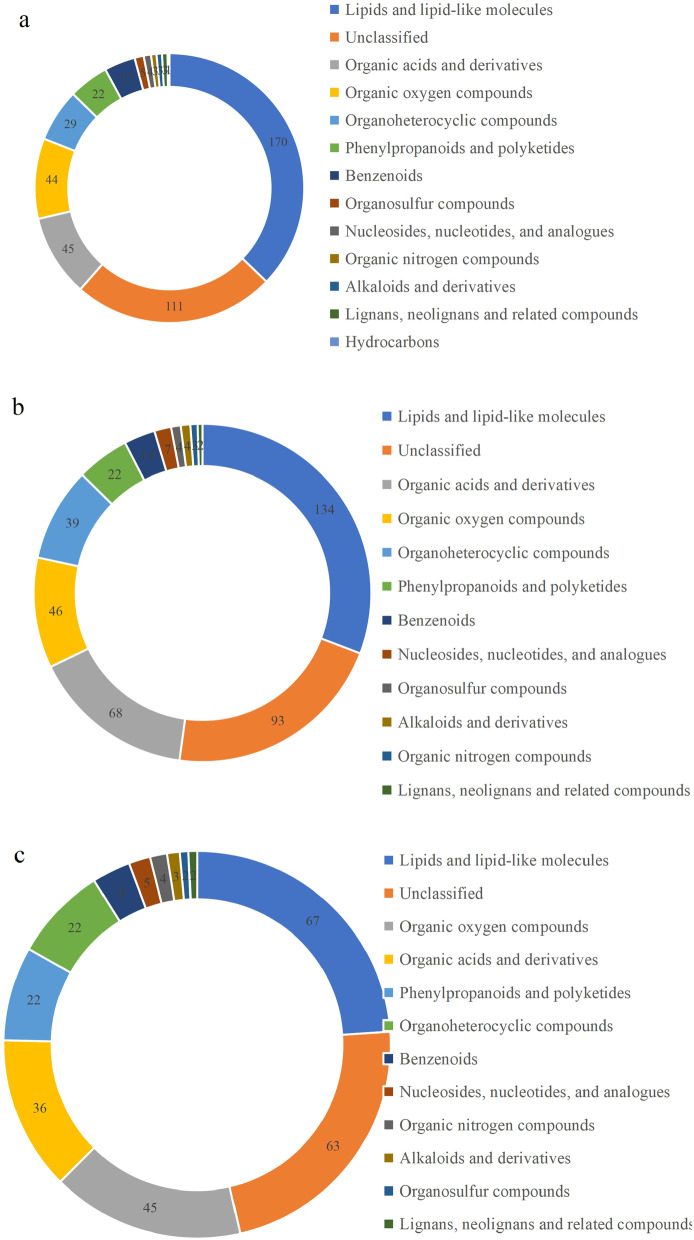
Distribution of DAM classes. Note: (**a**) T1 VS CK; (**b**) T2 VS CK

The DAMs were filtered based on a variable important in projection (VIP) > 1 and a *p-*value < 0.05 (T test results). Finally, a total of 457 DAMs (331 in positive and 126 in negative ESI modes) were common in T1 VS CK (Fig. 3) (Supplementary Table S5). The distribution of the DAM classes is shown in Fig. 4a, which includes 170 lipids and lipid-like molecules (77 fatty acyls, 37 glycerophospholipids, 20 prenol lipids, 11 steroids and steroid derivatives, 10 glycerolipids, 8 polyketides, and 7 sphingolipids); 45 organic acids and derivatives (39 carboxylic acids and derivatives, 2 hydroxy acids and derivatives, 1 keto acid and derivatives, 1 carboximidic acid, 1 thiophosphoric acid ester, and 1 organic phosphonic acid); 44 organic oxygen compounds, 29 organoheterocyclic compounds (5 indoles and derivatives, 3 benzopyrans, 3 quinolines and derivatives, 3 pyridines and derivatives, 2 imidazopyrimidines, 2 azoles, 2 lactones, 1 benzodiazepine, 1 pyrazolopyrimidine, 1 isobenzofuran, 1 diazine, 1 tetrahydrofuran, 1 epoxide, 1 pyrrolidine, 1 pteridines and derivative, and 1 heteroaromatic compound); 22 phenylpropanoids and polyketides (12 flavonoids, 3 linear 1,3-diarylpropanoids, 2 phenylpropanoic acids, 1 diarylheptanoid, 1 stilbene, 1 cinnamyl alcohol, 1 tannin, and 1 coumarin and its derivatives); 17 benzenoids (12 benzene and substituted derivatives, 2 naphthalenes, 2 phenols, and 1 dibenzocycloheptene); 5 organosulfur compounds (thiol, isothiocyanate, thioether, thioacetal, and sulfenyl compounds); 4 nucleosides, nucleotides, and analogues (3 purine nucleosides, and 1 nucleoside and nucleotide analogue); 3 organic nitrogen compounds, 3 alkaloids and derivatives (cytochalasin, harmala alkaloid, and aporphine); 3 lignans, neolignans and related compounds (2 lignan glycosides, and furanoid lignan); and 1 hydrocarbon. A total of 111 metabolites were unclassified.

A total of 435 DAMs (306 identified in positive ESI mode and 129 in negative ESI mode) were identified in T2 VS CK (Supplementary Table S5), in which 30 more metabolites were detected in positive ESI mode compared with that in T1 VS CK, and these metabolites were divided into 12 classes (Fig. 4b); hydrocarbons were not detected. Organic acids and derivatives and organoheterocyclic compounds in T2 VS CK were different from T1 VS CK not only in quantity but also in variety. In total, 68 metabolites belonged to organic acids and derivatives, which were assigned to four groups: carboxylic acids and derivatives, keto acids and derivatives, hydroxy acids and derivatives, and peptidomimetics. The first three compounds were also detected in T1 VS CK. Organoheterocyclic compounds included 23 compounds, among which azolidines, benzothiazines, cycloheptathiophenes, lactams, oxazinanes, piperazinoazepines, tetrapyrroles and derivatives, and triazines were not detected in T1 VS CK.

For the above statistical analysis, 280 critical metabolites detected in positive (Supplementary Table S6) and negative ion modes (Supplementary Table S7) were responsible for the metabolic changes between T1 and T2, and a heatmap of DAMs with the top 50 VIP values is shown in Fig. S6.

**Fig. 5:**
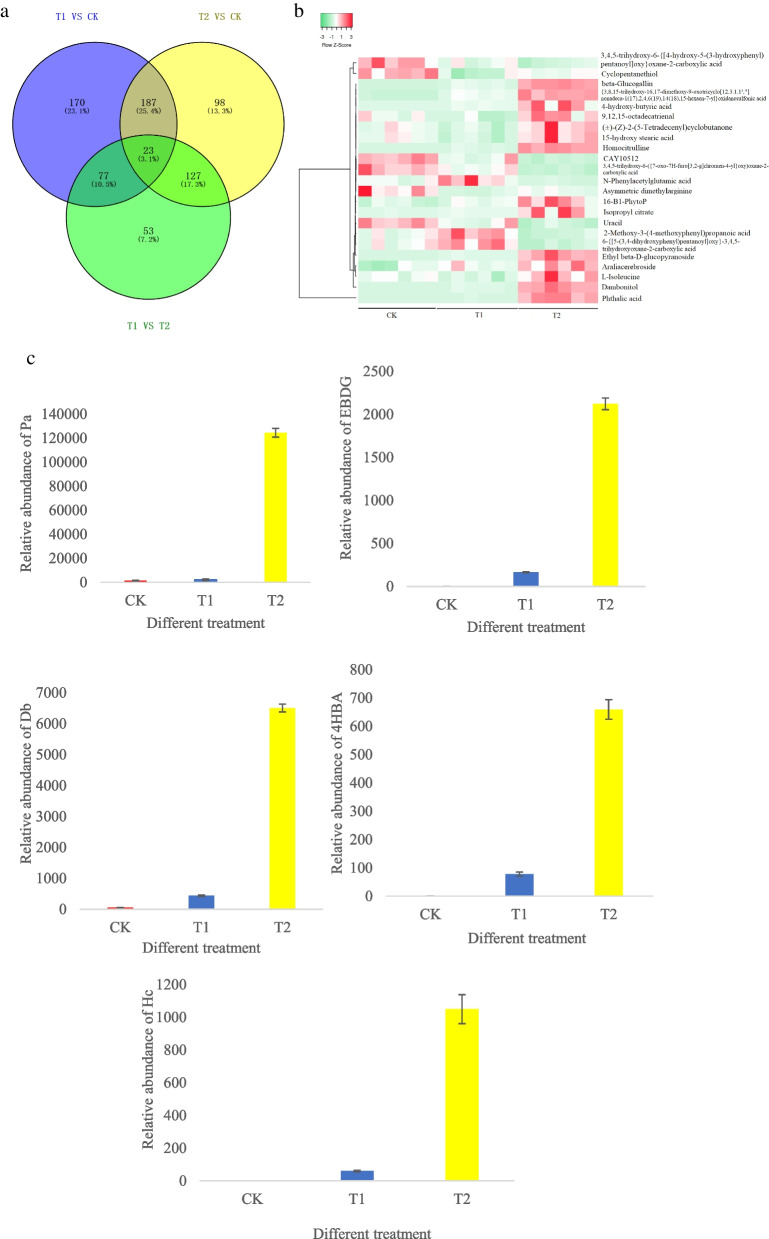
DAMs in all treatment groups of *P. cornutum* (L.) Gaertn. seedlings. Note: **a** Venn diagram of metabolites among all the treatment groups; **b** heatmap of metabolites among all the treatment groups; **c** relative abundances of Pa (phthalic acid), EBDG (ethyl β-D-glucopyranoside), Db (dambonitol), 4HBA (4-hydroxy-butyric acid), Hc (homocitrulline) in all the treatment groups

Based on comparative analysis of the significant DAM, a total of 23 (3.1%) metabolites specific to the three treatments were identified (Fig. 5a). Among these 23 DAMs, as the concentration of phthalic acid increased, the accumulation of 10 DAMs increased: phthalic acid (Pa), dambonitol (Db), araliacerebrosid, {3,8,15-trihydroxy-16,17-dimethoxy-9-oxotricyclo[12.3.1.1^2^,^6^]nonadeca-1(17),2,4,6(19),14(18),15-hexaen-7-yl}oxidanesulfonic acid, 16-B1-PhytoP, 4-hydroxy-butyric acid (4HBA), isopropyl citrate, homocitrulline (Hc), β-glucogallin, and ethyl β-D-glucopyranoside (EBDG). However, the accumulation of 4 DAMs (3,4,5-trihydroxy-6-{[4-hydroxy-5-(3-hydroxyphenyl) pentanoyl]oxy}oxane-2-carboxylic acid, uracil, 3,4,5-trihydroxy-6-({7-oxo-7H-furo[3,2-g]chromen-4-yl}oxy)oxane-2-carboxylic acid, and CAY10512) decreased significantly in all the treatment groups (Fig. 5b). Interestingly, among all the DAMs, the relative abundances of Pa, EBDG, Db, 4HBA, and Hc in T2 were 100 times greater than those in the CK, and these metabolites were rarely observed in the CK (Fig. 5c).

### Integrated analysis of the transcriptome and metabolome

To demonstrate the biosynthetic and regulatory characteristics of the top 20 DAMs and DEGs, subnetworks were constructed to show transcript–metabolite correlations (Supplementary Fig. S7). At the same time, increased and decreased metabolites between treatments were analysed. The findings showed that L-histidinol levels were higher in the T1 treatment than in the CK and T2 treatment, suggesting that L-histidinol played an important role in improving the growth of *P. cornutum* (L.) Gaertn. seedlings. A total of 11 DEGs were significantly positively correlated with L-histidinol content (Supplementary Fig. S8). Among them, the functions of TRINITY_DN15768_c0_g1_i1_2, TRINITY_DN16516_c0_g4_i1_1, TRINITY_DN23508_c0_g1_i1_2, TRINITY_DN24189_c0_g1_i2_2, TRINITY_DN24189_c0_g4_i2_ 2, and TRINITY_DN8295_c0_g1_i2_1 were the same as those of heat shock proteins (HSPs); TRINITY_DN15779_c0_g3_i1_3, and TRINITY_DN22187_c0_g2_i4_2 were annotated to aquaporins; TRINITY_DN9909_c0_g1_i1_1 encoded a cysteine protease; and TRINITY_DN8291_c0_g1_i1_1 and TRINITY_DN18787_c0_g1_i2_2 were not annotated in any database.

Phthalic acid (10 mmol·L^− 1^) was added to the water used to irrigate the *P. cornutum* (L.) Gaertn. seedlings. The roots absorbed and accumulated a large amount of phthalic acid, and then its content was greatest. Seventeen DEGs were significantly associated with phthalic acid (Supplementary Fig. S9). TRINITY_DN10949_c0_g1_i1_1 (cysteine-type peptidase activity), TRINITY_DN11148_c0_g1_i1_1 (defensin-like protein), TRINITY_DN12586_c0_g1_i3_2 (response to biotic stimulus), TRINITY_DN16111_c2_g2_i1_3 (glycine-rich protein), TRINITY_DN17657_c0_g1_i4_1 (jacalin-related lectin), TRINITY_DN18006_c1_g1_i1_1 (major latex), TRINITY_DN18632_c0_g2_i2_1 (defensin-like protein), TRINITY_DN21631_c0_g1_i1_2 (β-D-glucopyranosyl abscisate β-glucosidase), and TRINITY_DN23734_c1_4_i1_2 (nonclassic arabinogalactan protein) were significantly negatively correlated with phthalic acid.

TRINITY_DN14863_c0_g1_i1_2 (HSP), TRINITY_DN14945_c0_g2_i2_1 (ubiquitin-mediated proteolysis), TRINITY_DN14949_c0_g2_i1_2 (response to water deprivation),

TRINITY_DN15768_c0_g1_i1_2 (HSP), TRINITY_DN17933_c0_g2_i1_2, TRINITY_DN23638_c1_g3_i1_2, TRINITY_DN9949_c0_g1_i1_3 (glutathione S-transferase), and TRINITY_DN24538_c0_g1_i10_2 (glyceraldehyde-3-phosphate dehydrogenase) were significantly positively correlated with phthalic acid.

### Effects of N and C metabolism in *P. cornutum* (L.) Gaertn. Roots treated with phthalic acid

**Fig. 6:**
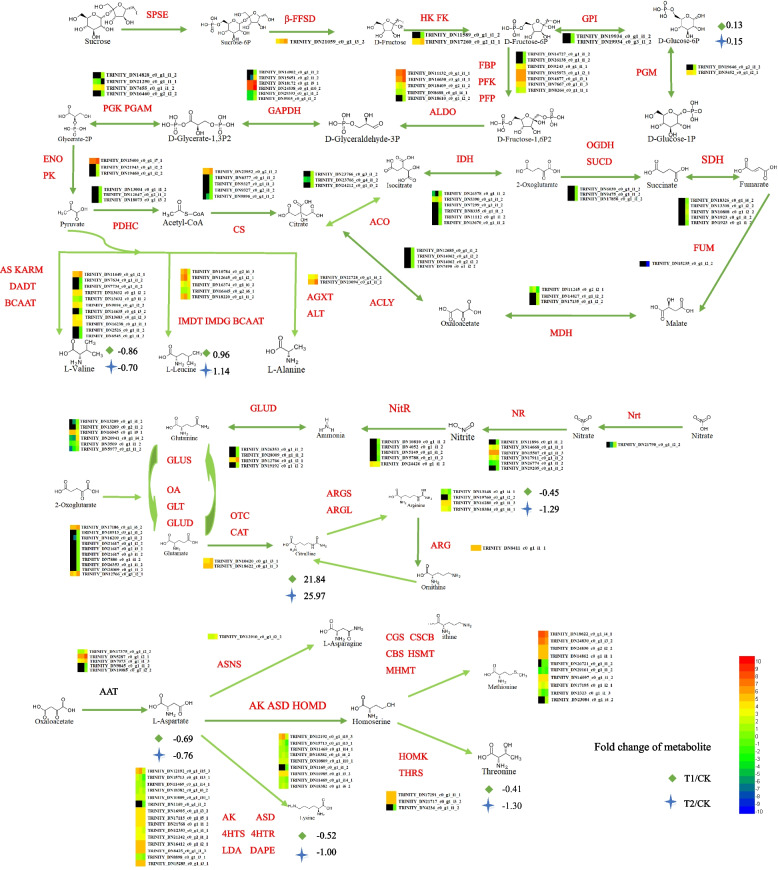
Carbon and nitrogen uptake and transport in roots of *P. cornutum* (L.) Gaertn. treated with phthalic acid. Note: The log_2_(ratios) of the genes and metabolites are shown, and the colour key is presented to the right of the figure. The red characters represent genes, and the black characters with no background represent metabolites. From left to right, the three squares illustrate changes in gene expression under CK, T1 and T2 treatment. *SPSE*, sucrose PTS system; EIIBCA or EIIBC component, *β-FFSD* β-fructofuranosidase; *HK*, hexokinase, *FK*, fructokinase; *GPI*, glucose-6-phosphate isomerase; *PGM*, phosphoglucomutase; *FBP*, fructose-1,6-bisphosphatase I; *PFK*, 6-phosphofructokinase 1; *PFP*, diphosphate-dependent phosphofructokinase; *ALDO*, fructose-bisphosphate aldolase; *GAPDH*, glyceraldehyde 3-phosphate dehydrogenase; *PGK*, phosphoglycerate kinase; *PGAM*, 2,3-bisphosphoglycerate-dependent phosphoglycerate mutase; *ENO*, enolase; *PK*, pyruvate kinase; *AS*, acetolactate synthase I/II/III large subunit; *KARM*, ketol-acid reductoisomerase; DADT, dihydroxy-acid dehydratase; *BCAAT*, branched-chain amino acid aminotransferase; *IMDT*, isopropylmalate dehydratase; *IMDG*, 3-isopropylmalate dehydrogenase; *BCAAT*, branched-chain amino acid aminotransferase; *AGXT*, alanine-glyoxylate transaminase/(R)-3-amino-2-methylpropionate-pyruvate transaminase; *ALT*, alanine transaminase; *PDE*, pyruvate dehydrogenase E1 component; *PDHC*, pyruvate dehydrogenase E2 component (dihydrolipoamide acetyltransferase); *CS*, citrate synthase; *ACO*, aconitate hydratase; *IDH*, isocitrate dehydrogenase; *OGDH*, 2-oxoglutarate dehydrogenase E1 component; *SUCD*, succinyl-CoA synthetase alpha subunit; *SDH*, succinate dehydrogenase (ubiquinone) flavoprotein subunit; *FUM*, fumarate hydratase; *MDH*, malate dehydrogenase; *ACLY*, ATP citrate (pro-S)-lyase; *AAT*, aspartate aminotransferase; *ASNS*, asparagine synthase; *AK*, aspartate kinase; *ASD*, aspartate-semialdehyde dehydrogenase; *HOMD*, homoserine dehydrogenase; *HOMK*, homoserine kinase; *THRS*, threonine synthase; *CGS*, cystathionine gamma-synthase; *CSCB*, cysteine-S-conjugate β-lyase; *CBS*, cysteine-S-conjugate β-lyase; *HSMT*, homocysteine S-methyltransferase; *MHMT*, 5-methyltetrahydropteroyltriglutamate--homocysteine methyltransferase; *AK*, aspartate kinase; *ASD*, aspartate-semialdehyde dehydrogenase; *4HTS*, 4-hydroxy-tetrahydrodipicolinate synthase; *4HTR*, 4-hydroxy-tetrahydrodipicolinate reductase; *LDA*, L-diaminopimelate aminotransferase; *DAPE*, diaminopimelate epimerase; *DAPD*, diaminopimelate decarboxylase; *OA*, omega-amidase; *GLT*, glutamate synthase; *GLUD*, glutamate dehydrogenase; *GLUS*, glutamine synthetase; *ACD*, acetylornithine deacetylase; *OTC*, ornithine carbamoyltransferase; *ARGS*, argininosuccinate synthase; *ARGL*, argininosuccinate lyase; *ARG*, arginase; *Nrt*, nitrate/nitrite transporter; *NR*, nitrate reductase; *NitR*, ferredoxin-nitrite reductase

Carbon and nitrogen uptake and transport in roots of *P. cornutum* (L.) Gaertn. was affected by phthalic acid (Fig. 6). Analysis of the transcriptome and metabolome results yielded consistent results; they showed that glycolysis, the citric acid cycle, N uptake, assimilation, amino acid metabolism, and aminoacyl-tRNA biosynthesis were significantly different after treatment with phthalic acid.

The transcripts of genes associated with N uptake and assimilation in the roots of *P. cornutum* (L.) Gaertn. differed under different concentrations of phthalic acid treatment. In T2 VS T1, the nitrate transporter gene Nrt was upregulated, and 6 nitrate assimilation genes were annotated as nitrate reductase (NR); none of them was significantly expressed. Moreover, 5 ammonium assimilation (GLUS) genes were upregulated, and only one was downregulated. The accumulation of the metabolites arginine, L-aspartate, threonine, lysine, and L-valine decreased to a lesser degree in the T1 treatment than in the T2 treatment, while the accumulation of L-leucine, D-glucose-6P, and citrulline increased. Consistent with these findings, the asparagine synthase (ASNS) gene was upregulated to a lesser extent in the T1 treatment than in the T2 treatment, while the aspartate kinase (AK) genes were downregulated to a greater extent in the T1 treatment than in the T2 treatment.

### Combined physiological and molecular analysis of *P. cornutum* (L.) Gaertn. Treated with different concentrations of phthalic acid revealed the mechanism involved in autotoxicity

**Fig. 7:**
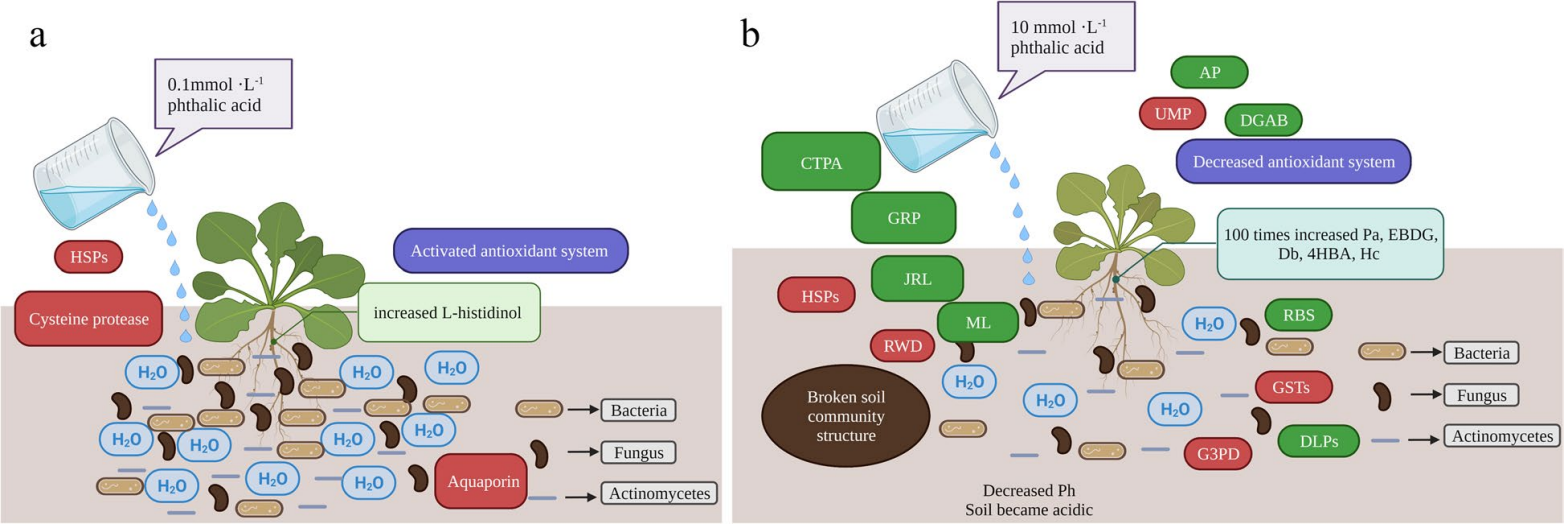
Proposed model for the mechanisms of autotoxicity in *P. cornutum* (L.) Gaertn. treated with phthalic acid. Note: **a** 0.1 mmol·L^− 1^ phthalic acid; **b** 10 mmol·L^− 1^ phthalic acid

The antioxidant system of *P. cornutum* (L.) Gaertn. seedlings treated with a low concentration of phthalic acid was activated [26], and the L-histidinol content in roots increased. The DEGs encoding HSPs, aquaporins, and cysteine proteases were upregulated (Fig. 7a).

After watering the *P. cornutum* (L.) Gaertn. seedlings with an aqueous solution with a high concentration of phthalic acid, the roots absorbed the phthalic acid from the soil, and the relative abundance of phthalic acid in the roots was up to 50 times higher than that when low concentrations of phthalic acid were used. The relative abundances of 4-hydroxy-butyric acid, ethyl β-D-glucopyranoside, dambonitol, and homocitrulline were 100 times higher than those in the CK. In addition, the soil moisture was also lower than that of the low-phthalic acid-concentration treatment. The accumulation of these substances and a decrease in moisture may be the cause of the inhibition of seedling growth and the decreased activity of antioxidant enzymes [26]. At the same time, phthalic acid is a strong acid at a high concentration, and with the prolongation of watering time, the soil pH was reduced, the soil became acidic, the number of bacteria, fungi and actinomycetes in the soil was reduced, and the community structure of the soil was destroyed, requiring follow-up experiments to verify changes in soil microbes. Cysteine-type peptidase activity, defensin-like proteins, response to biotic stimulus, glycine-rich protein, jacalin-related lectin, major latex, β-D-glucopyranosyl abscisate β-glucosidase, and nonclassic arabinogalactan protein-related DEGs were downregulated, but DEGs associated with HSPs, ubiquitin-mediated proteolysis, response to water deprivation, glutathione S-transferase, and glyceraldehyde-3-phosphate dehydrogenase were upregulated (Fig. 7b).

There were no pictures similar to the seedlings of *P. cornutum* (L.) Gaertn. in the mapping software; the plants in the picture were used instead of *P. cornutum* (L.) Gaertn. The plants in the picture are for reference only. The red boxes in the figure represent upregulated genes, and the green boxes represent downregulated genes. *Pa*, phthalic acid; *EBDG*, ethyl β-D-glucopyranoside; *Db*, dambonitol; *4HBA*, 4-hydroxy-butyric acid; *Hc*, homocitrulline; *CTPA*, cysteine-type peptidase activity; *DLP*, defensin-like protein; *RBS*, response to biotic stimulus; *GR*,*P* glycine-rich protein*; JRL*, jacalin-related lectin; *ML*, major latex; *DGAB*, β-D-glucopyranosyl abscisate β-glucosidase; *AP*, nonclassic arabinogalactan protein; *HSP*, heat shock protein; *UMP*, ubiquitin-mediated proteolysis; *RWD*, response to water deprivation; GST, glutathione S-transferases; *G3P*, glyceraldehyde-3-phosphate dehydrogenase.

## Discussion

Autotoxicity is an internal allelopathic effect that plays an important role in inhibiting plant growth and building plant communities [8]. The autotoxicity of *P. cornutum* (L.) Gaertn. not only restricts the distribution and protection of its wild resources but also affects its green-type cultivation and development and utilization in China. The formation of phytotoxicity is a complex process involving the interactions of plants, autotoxins and microorganisms [34–36]. Therefore, clarifying how *P. cornutum* (L.) Gaertn. response to autotoxins is key for a complete understanding of the formation mechanism of autotoxicity. The aqueous extracts of root exudates and residues of *P. cornutum* (L.) Gaertn can inhibit seed germination and seedling growth and has a significant inhibitory effect on the germination of some weeds. When exogenous organic compounds were tested for verification, phthalic acid was found to be a major autotoxin. Wu et al. [37] also found that phthalic acid was a principal autotoxin from root exudates of Lanzhou lily, which is known to cause soil toxicity by inducing autotoxicity. At present, research on *P. cornutum* (L.) Gaertn. seedlings treated with phthalic acid are focused on physiological and biochemical aspects [25, 26], but no reports at the transcriptomic or metabolic level are available. We constructed RNA-seq libraries of roots of *P. cornutum* (L.) Gaertn. seedlings treated with phthalic acid and obtained full metabolite information via LC–MS technology. Next-generation sequencing technology was used, which revealed 37,737 unigenes, of which 1085 (703 upregulated and 382 downregulated) and 5998 (4385 upregulated and 1613 downregulated) were differentially expressed in T1 and T2 compared with the CK, respectively. On the basis of the above data, the number of DEGs in T2 was much greater than that in T1, which was more than 5 times that of T1, and the number of upregulated genes in T1 and T2 was greater than the number of downregulated genes, demonstrating that *P. cornutum* (L.) Gaertn. was severely affected by phthalic acid. Chi et al. showed that more transcripts responded to short ferulic acid exposure (1- and 3-h treatments, 1204 genes) than long exposure (24 h, 176 genes) in rice roots; after short ferulic acid treatment, 972 genes were upregulated, and 232 were downregulated [38]. The above results showed that different autotoxins and different treatment methods have different changes in DEGs in different plant species. Through analysis of changed gene expression, we identified several metabolic pathways, including glycolysis, the citric acid cycle, N uptake, assimilation, amino acid metabolism, and aminoacyl-tRNA biosynthesis, that respond to autotoxicity in the roots of *P. cornutum* (L.) Gaertn.

### Phthalic acid-responsive changes to the transcriptome and metabolome

L-histidinol is a natural amino alcohol that serves as an intermediate in the biosynthesis of the amino acid L-histidine in plants [39]. L-histidine, one of the 20 standard amino acid constituents of proteins, plays a vital role in plant growth and development [40]. The results of this study showed that the content of L-histidinol in the roots of *P. cornutum* (L.) Gaertn. was increased, and biosynthesis of amino acids was one of the top 5 significantly enriched metabolic pathways under the treatment of 0.1 mmol·L^− 1^ phthalic acid. Eleven DEGs, of which 6 encoded HSPs, 2 were annotated to aquaporins, 1 belonged to a cysteine protease, and 2 were unknown, were significantly positively correlated with L-histidinol content. HSPs are members of a highly conserved family of proteins that present in all biological kingdoms that aid in cell protection, protein homeostasis and cell survival against a variety of environmental and metabolic stresses [41–43] and help to degrade irreparable proteins and toxins to limit their accumulation [44, 45]. HSPs play important roles in genome control and ultimately lead to distinct characteristics by mediating signalling mechanisms, host defence mechanisms, translation, carbohydrate metabolism, and amino acid metabolism [46]. Overexpression of HSPs prior to injury has a protective effect [47]. Christou et al. showed that NaHS treatment of strawberry roots resulted in increased expression levels of HSP (HSP70, HSP80, HSP90) and aquaporin genes [48]. HSP18.5, HSP17.1 and HSP17.3 as well as several other HSP genes were downregulated in apple replant disease samples [49]. However, in the present study, HSPs were upregulated under both the 0.1 mmol·L^− 1^ and the 10 mmol·L^− 1^ phthalic acid treatments in the roots of *P. cornutum* (L.) Gaertn.

Aquaporins are involved in the regulation of osmotic pressure and maintenance of cell expansion during the volume expansion phase of rapidly growing cells and mediate the rapid entry or export of water when osmotic pressure is abrupt. By transporting water and other small molecules, they play a crucial role in alleviate abiotic stress to maintain cellular homeostasis, and regulation of AQP activity and gene expression has also been considered part of an adaptive mechanism to stressful conditions [50, 51]. Previous studies have shown that a low abundance of AQP proteins decreases water permeability, while a high abundance (overexpression) increases hydraulic conductivity in biofilms [52]. Although the DEGs TRINITY_DN15779_c0_g3_i1_3 and TRINITY_DN22187_c0_g2_i4_2, which are related to aquaporins, were upregulated in this study and hydraulic conductivity increased, the growth of *P. cornutum* (L.) Gaertn. decreased. These results were similar to the findings of Refael et al., who found that overexpression of the AQP gene AtPIP1b in *Arabidopsis* decreased the growth in *Nicotiana tabacum* under drought stress conditions [53]. This result indicated that aquaporins played a certain role in resisting the autotoxicity of *P. cornutum* (L.) Gaertn. seedlings, albeit not a decisive role.

### Analysis of the formation mechanism of the autotoxicity of *P. cornutum* (L.) Gaertn

Elevated reactive oxygen species (ROS) levels are important components of stress caused by allelochemicals [54]. Our previous study showed that the H_2_O_2_ and O_2_^-^ production rates and malondialdehyde (MDA) content in *P. cornutum* (L.) Gaertn. seedlings treated with 10 mmol·L^− 1^ phthalic acid increased, while the enzymatic activities of the antioxidant enzyme system decreased [26]. Chi et al. showed that with increasing ferulic acid concentration in the roots of *Oryza sativa*, the root growth rate decreased, and reactive oxygen species increased [38]. The reasons why the high -concentration-phthalic acid treatment inhibited the growth of *P. cornutum* (L.) Gaertn. seedlings may have occurred for several reasons. First, the roots of *P. cornutum* (L.) Gaertn. seedlings absorbed phthalic acid from soil and accumulated a large amount of phthalic acid; at the same time, the contents of 4-hydroxy-butyric acid, ethyl β-D-glucopyranoside, dambonitol, and homocitrulline were also high. Second, high-concentration phthalic acid treatment reduced the amount of irrigation water and affected the uptake and transport of water, carbon and nitrogen by *P. cornutum* (L.) Gaertn. seedlings. The response to water deprivation was enhanced. Finally, phthalic acid is a strong acid, and high-concentration treatments reduced the number of bacteria, fungi, and actinomycetes and disrupted the community structure in the soil, potentially disrupting the balance between beneficial and harmful microorganisms and resulting in fewer beneficial microorganisms that promote seedling growth. Previous studies have confirmed that continuous cropping can decrease number of the bacterial species and decrease bacterial community structure, increasing the number of harmful microorganisms and decreasing the number of beneficial microorganisms [55, 56]. However, how microbes change after treatment with phthalic acid specifically requires further research. In addition, abscisate is a phytohormone critical for plant growth, development and adaptation to various stress conditions [57]. β-D-gluranosyl abscisate β-glucosidase hydrolyses the biologically inactive β-D-glucopyranosyl ester of abscisic acid to produce active abscisate. Downregulation of the gene encoding it may result in no or less synthesis of abscisate, reducing the ability of plants to resist biotic or abiotic stress. The product of the defensin gene (BSD1) showed antifungal activity against several phytopathogenic fungi [58], and transgenic tobacco plants expressing the defensin gene (RS-AFP1) showed enhanced resistance to fungal pathogens [59]. Glycine-rich proteins accumulate in vascular tissues, and their synthesis is part of the plant defence mechanisms [60]. The downregulated genes encoding defensin-like proteins and glycine-rich proteins and those involved in the response to biotic stimuli led to the decline in the defence mechanism and reduction in the ability to resist phytopathogenic fungi, which is not conducive to the growth of *P. cornutum* (L.) Gaertn. seedlings.

## Conclusions

The growth of *P. cornutum* (L.) Gaertn. seedlings is suppressed by high concentrations of phthalic acid, which accumulates in the roots of *P. cornutum* (L.) Gaertn. seedlings and affects the absorption and transport of water, carbon and nitrogen. DEGs encoding HSPs and those involved in ubiquitin-mediated proteolysis, response to water deprivation, glutathione S-transferase, and glyceraldehyde-3-phosphate dehydrogenase significantly associated with phthalic acid were upregulated. These findings have increased our understanding of the molecular mechanism of replant disease and provide a theoretical basis for solving problems associated with medicinal plants in China.

## Supplementary Information

**Additional file 1: Fig. S1.** RNA-Seq experiment process. **Fig. S2.** Sample preparation process for LC-MS. **Fig. S3.** Validation of Illumina sequencing data by real-time PCR. **Fig. S4.** PCA score. **Fig. S5.** Cluster analysis. **Fig. S6.** Heatmap of DAMs. **Fig. S7.** The correlations of the top 20 transcript and metabolite. **Fig. S8.** Correlation network diagram of DEGs and DAMs. **Fig. S9.** Correlation analysis of DEGs and DAMs.

**Additional file 2: Table S1.** Primer sequences. **Table S2.** RNA-seq data statistics. **Table S3.** The KEGG Enrichment analysis of DEGs between CK and T1. **Table S4.** The KEGG Enrichment analysis of DEGs between CK and T2. **Table S5.** The classes of metabolites. **Table S6.** The positive ion modes of metabolites between T1 and T2. **Table S7.** The negative ion modes of metabolites between T1 and T2.

## Data Availability

All sequence data for the samples have been deposited in the Short Read Archive (SRA) of the NCBI database under the following accession numbers: PRJNA828972 (https://www.ncbi.nlm.nih.gov/Traces/study/?acc=PRJNA828972).

## Abbreviations

*P. cornutum* (L.) Gaertn.: *Pugionium cornutum* (L.) Gaertn.
CK: Control
T1: 0.1 mmol·L^− 1^ phthalic acid treatment
T2: 10 mmol·L^− 1^ phthalic acid treatment
DAMs: Differentially accumulated metabolites
DEGs: Differentially expressed genes
HSPs: Heat shock proteins
ROS: Reactive oxygen species
H_2_O_2_: Hydrogen peroxide
O^2-^: Superoxide anion
MDA: Malondialdehyde
