# Supplementary material for: Responses of transcriptome and metabolome in the roots of Pugionium cornutum (L.) Gaertn to exogenously applied phthalic acid
Source: BMC Plant Biol. 2022 Nov 17;22:535. doi: 10.1186/s12870-022-03927-9 (PMC9670373; doi:10.1186/s12870-022-03927-9)
Supplement: Supplementary file 1 — Additional file 1: Fig. S1. RNA-Seq experiment process. Fig. S2. Sample preparation process for LC-MS. Fig. S3. Validation of Illumina sequencing data by real-time PCR. Fig. S4. PCA score. Fig. S5. Cluster analysis. Fig. S6. Heatmap of DAMs. Fig. S7. The correlations of the top 20 transcript and metabolite. Fig. S8. Correlation network diagram of DEGs and DAMs. Fig. S9. Correlation analysis of DEGs and DAMs. [file 12870_2022_3927_MOESM1_ESM.doc]

**
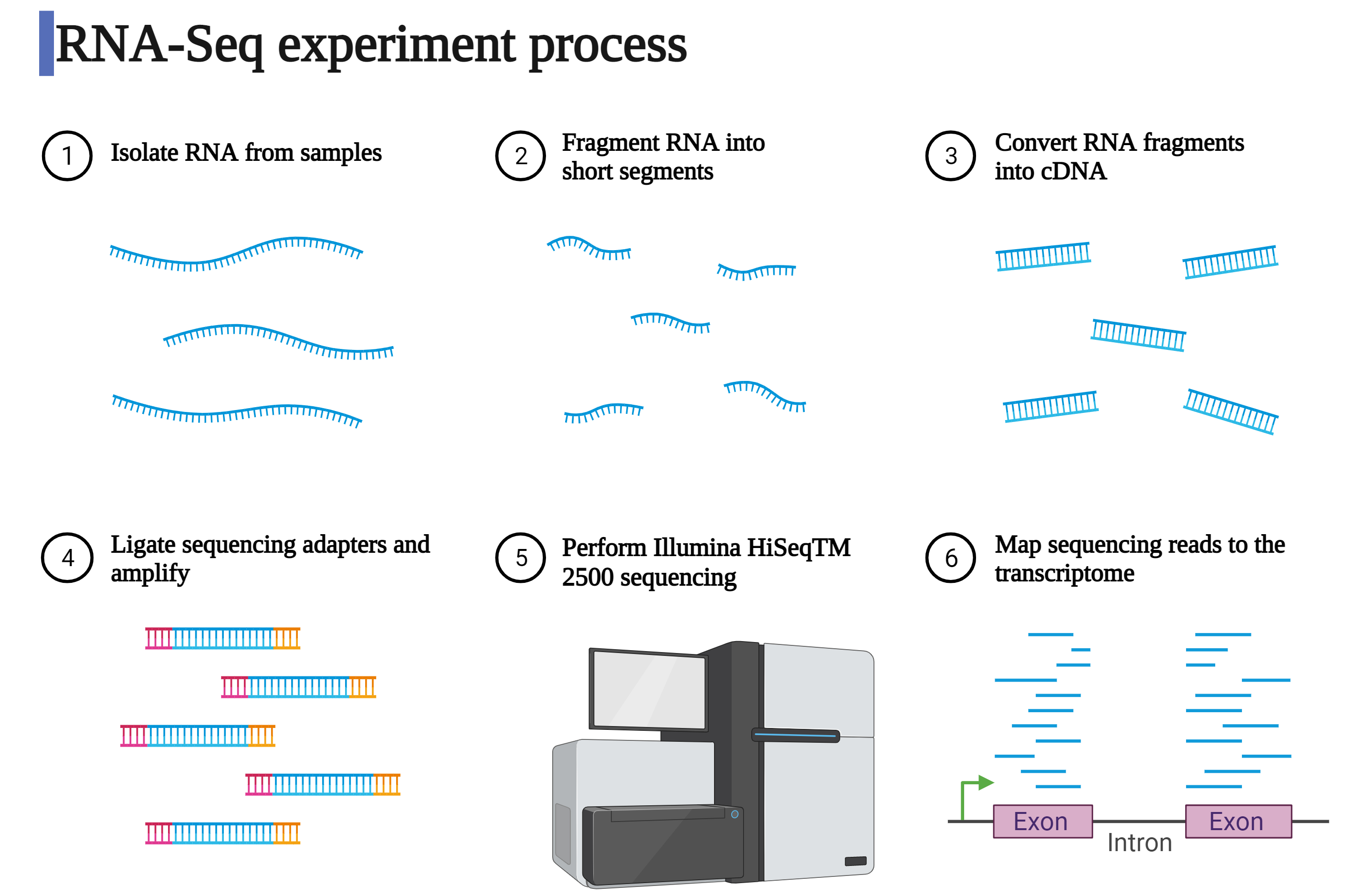
**

Fig. S1 RNA-Seq experiment process


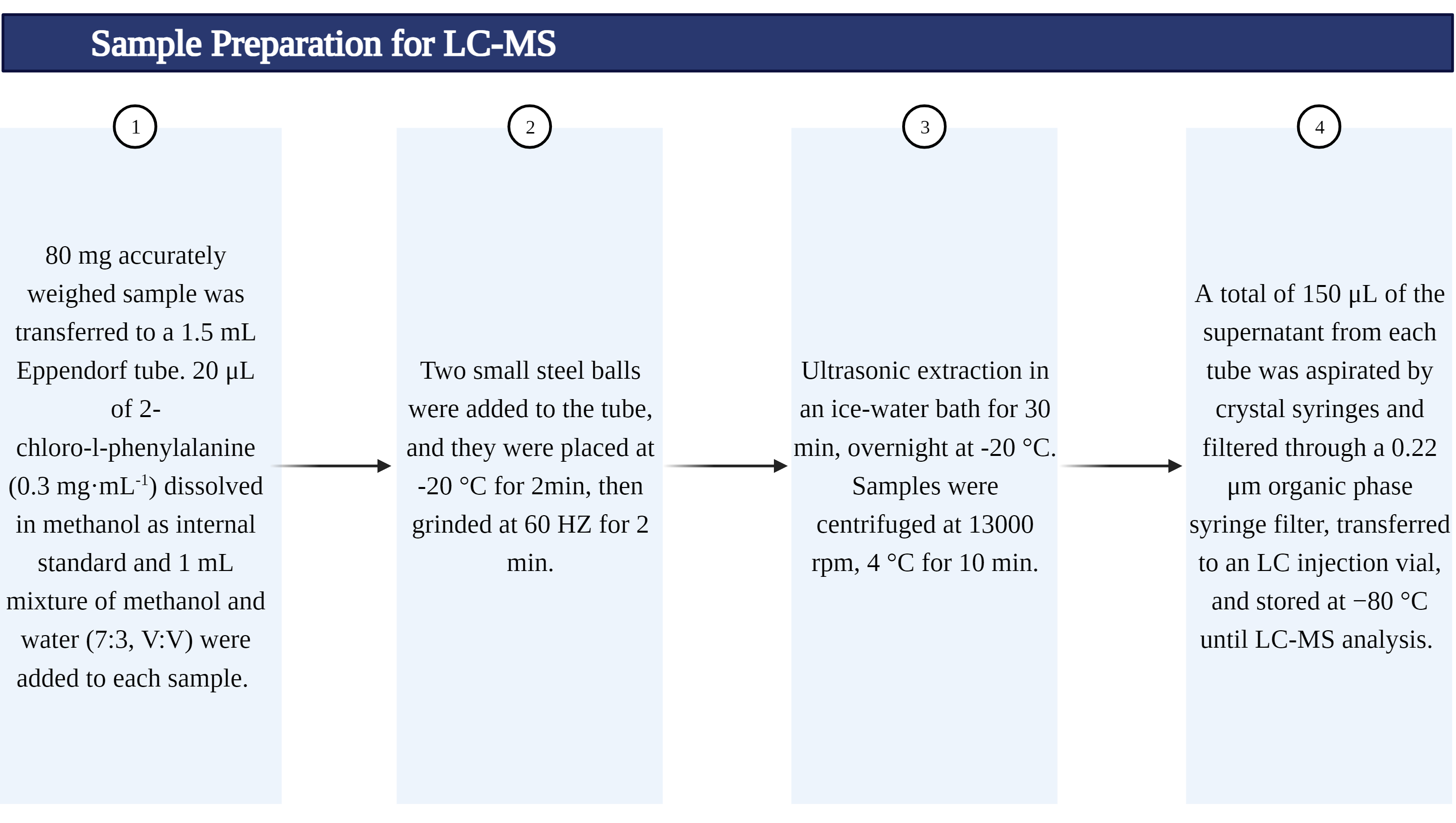


Fig. S2 Sample preparation process for LC-MS


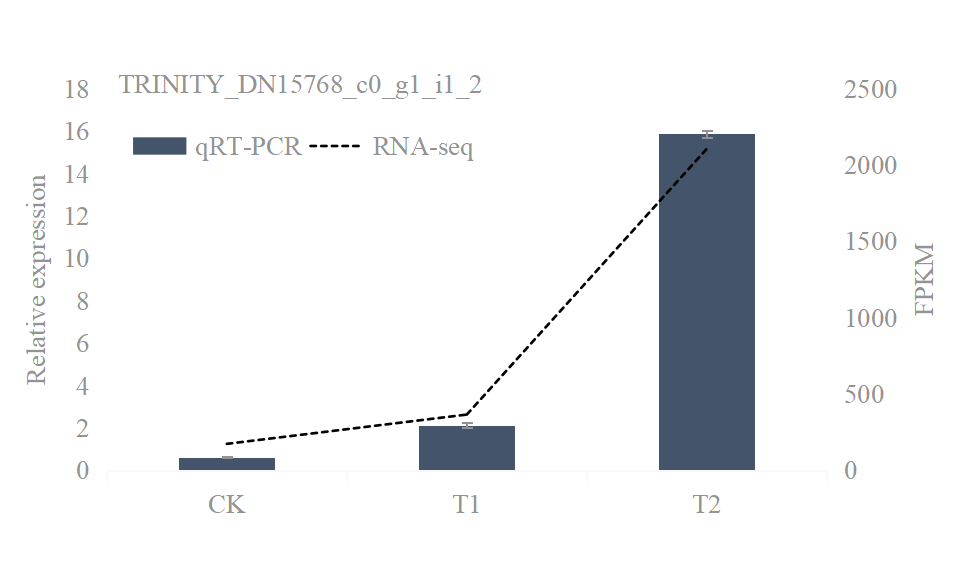


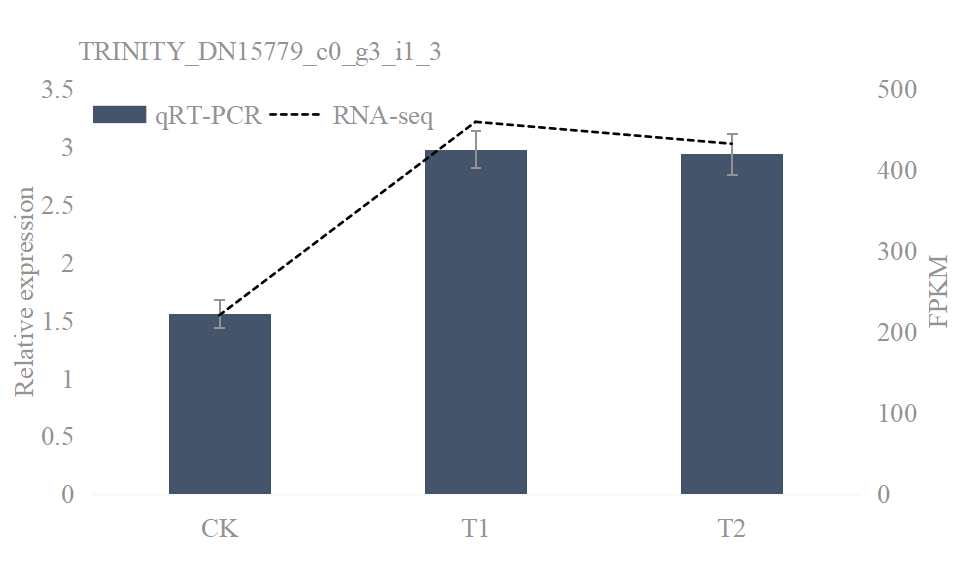


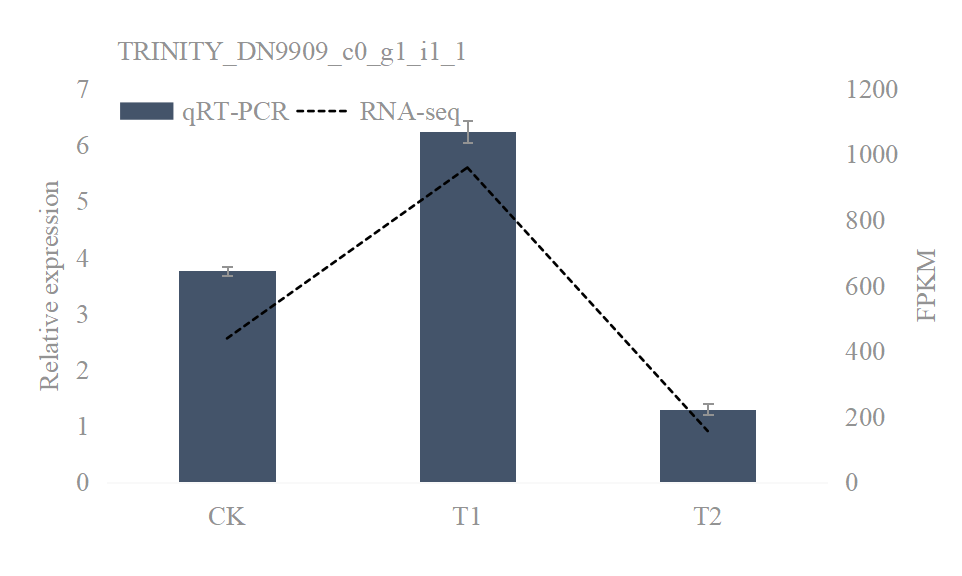


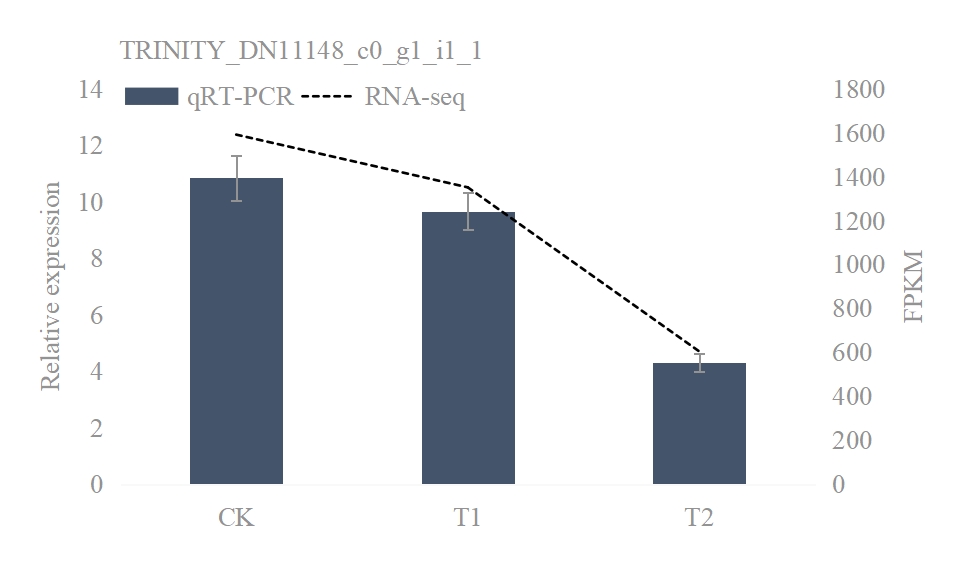


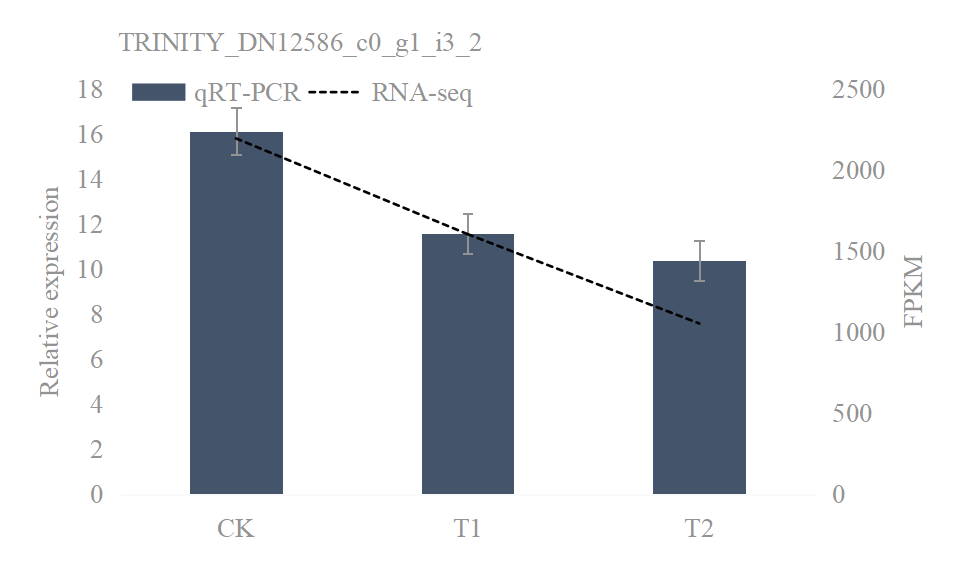


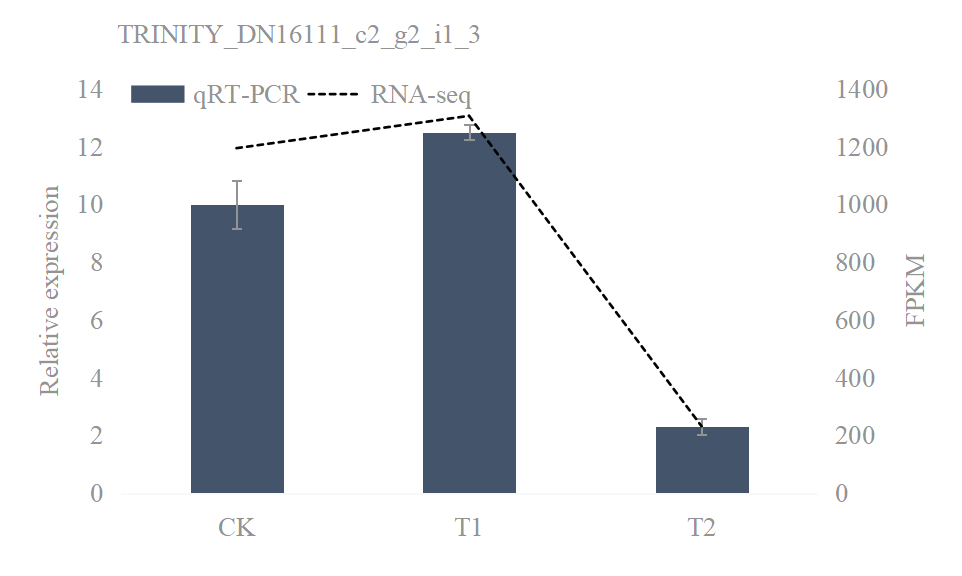


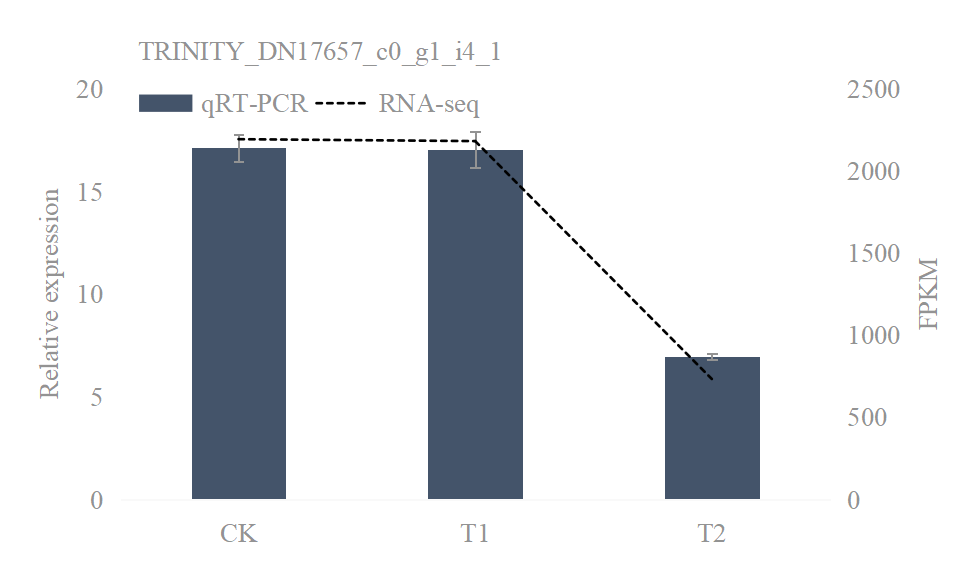


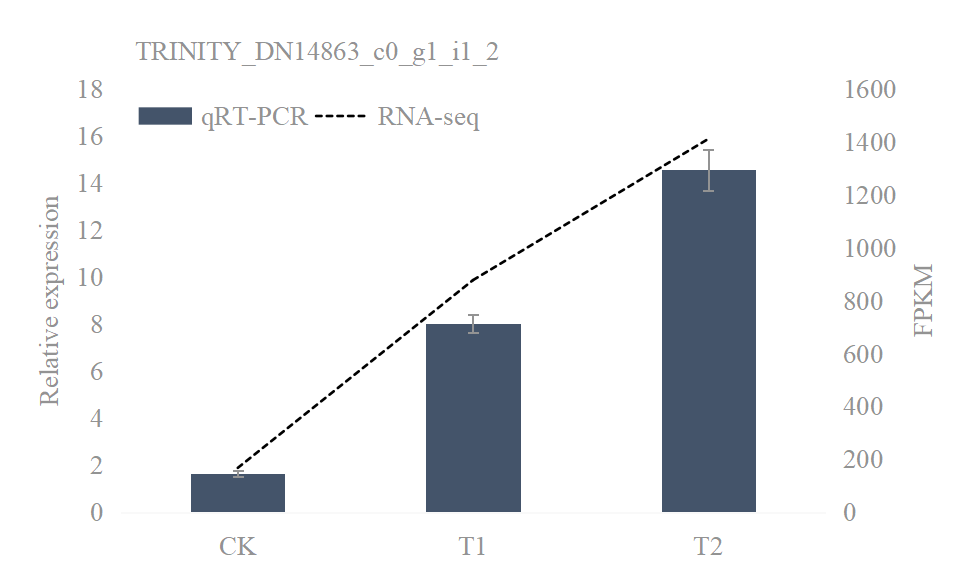


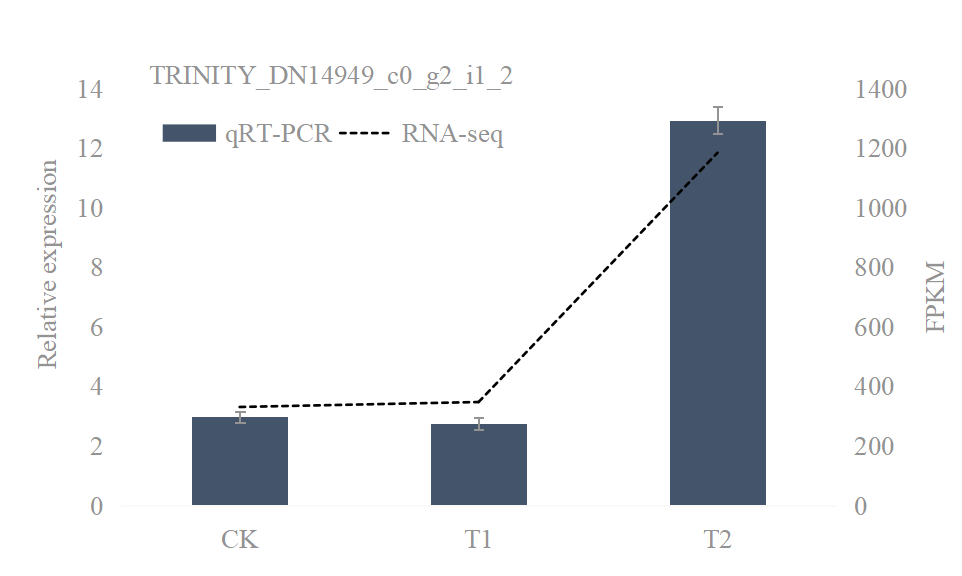


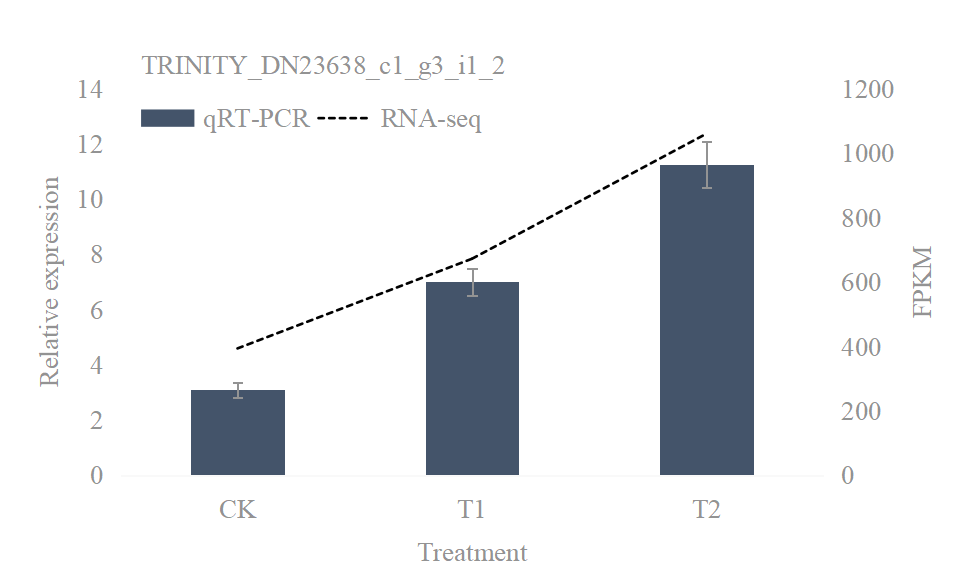


Fig. S3 Validation of Illumina sequencing data by real-time PCR

Note: The main axis represents the relative expression, and the secondary axis represents FPKM.


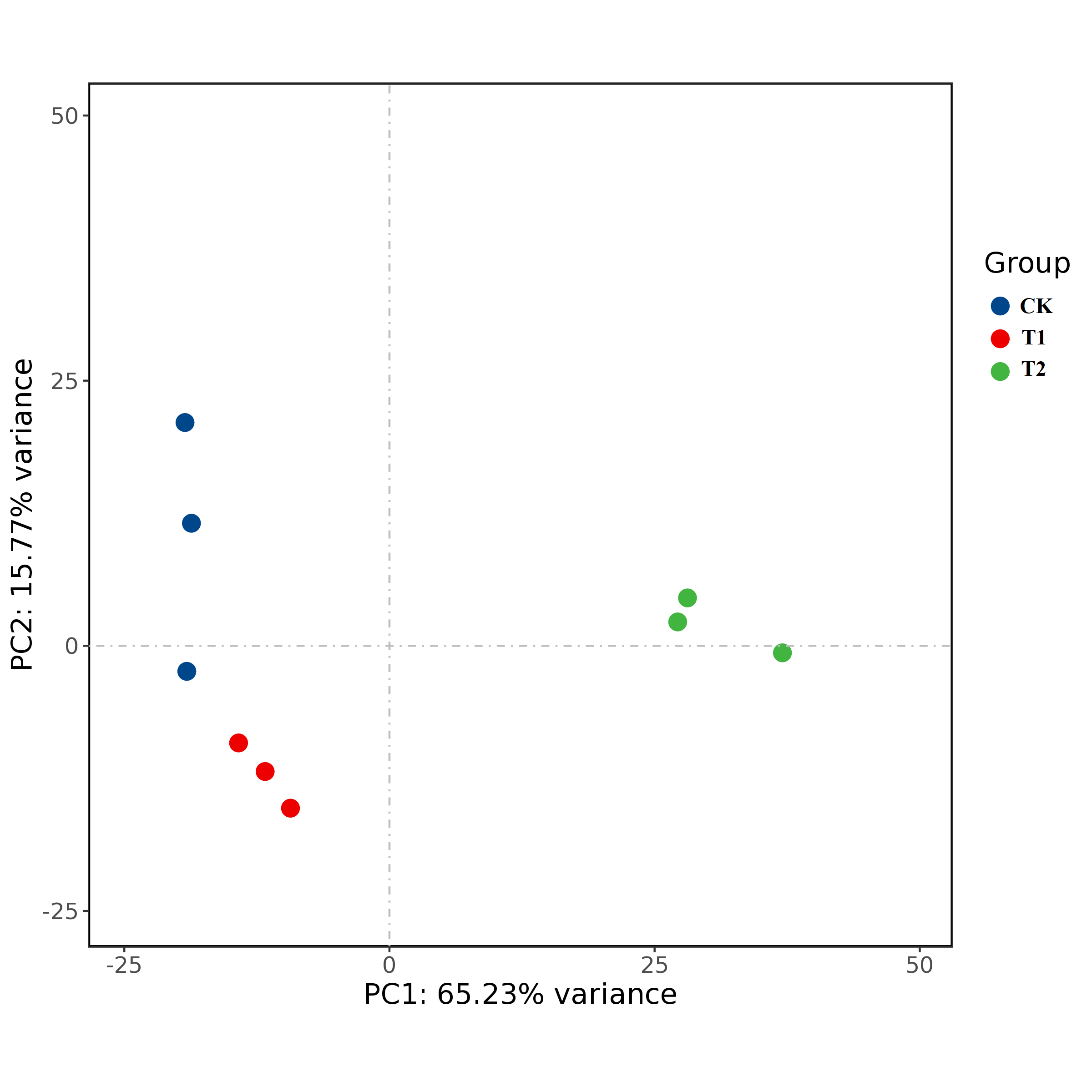


Fig. S4 PCA score


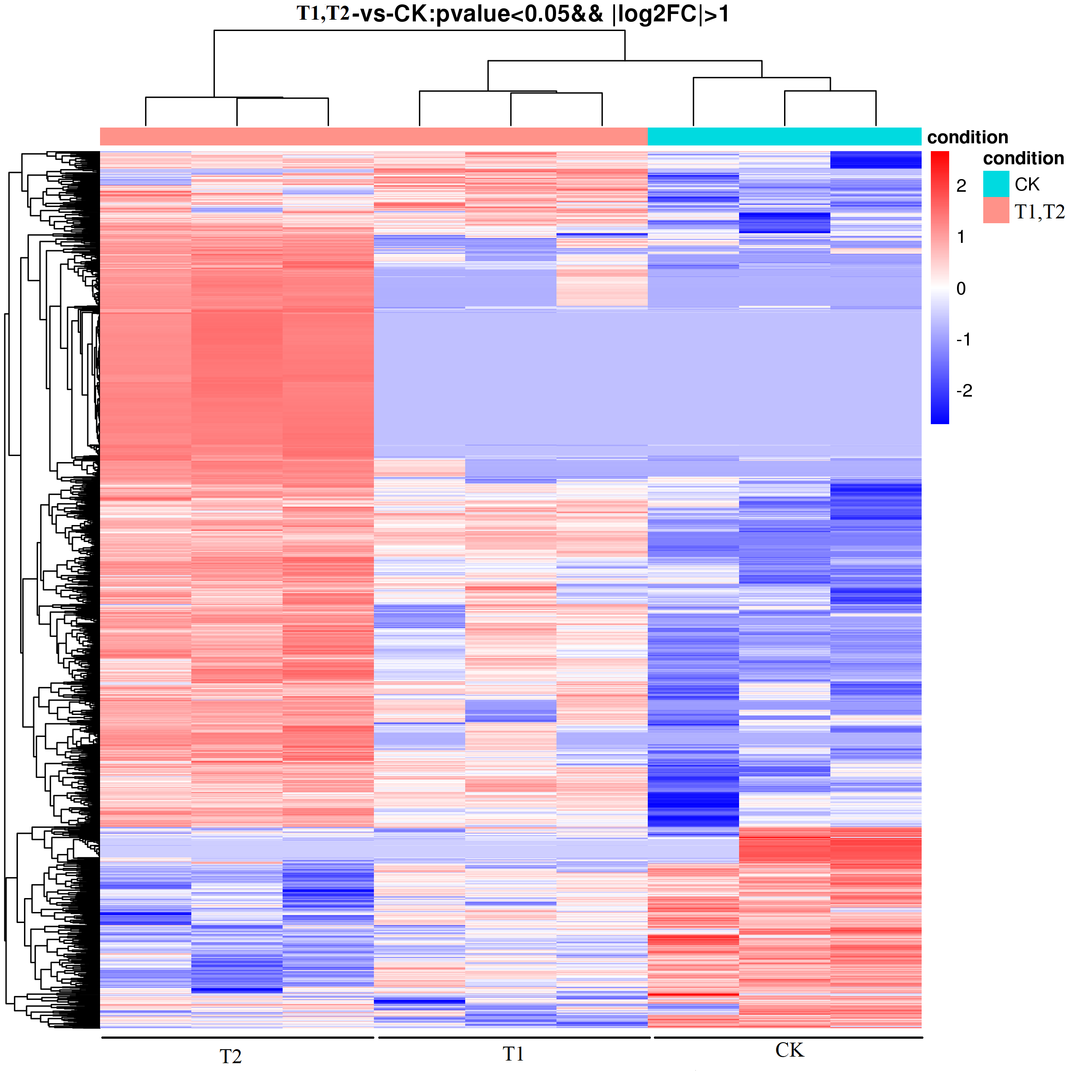


Fig. S5 Cluster analysis

Note: red indicated high expression of unigene, and blue indicated low expression of unigene.


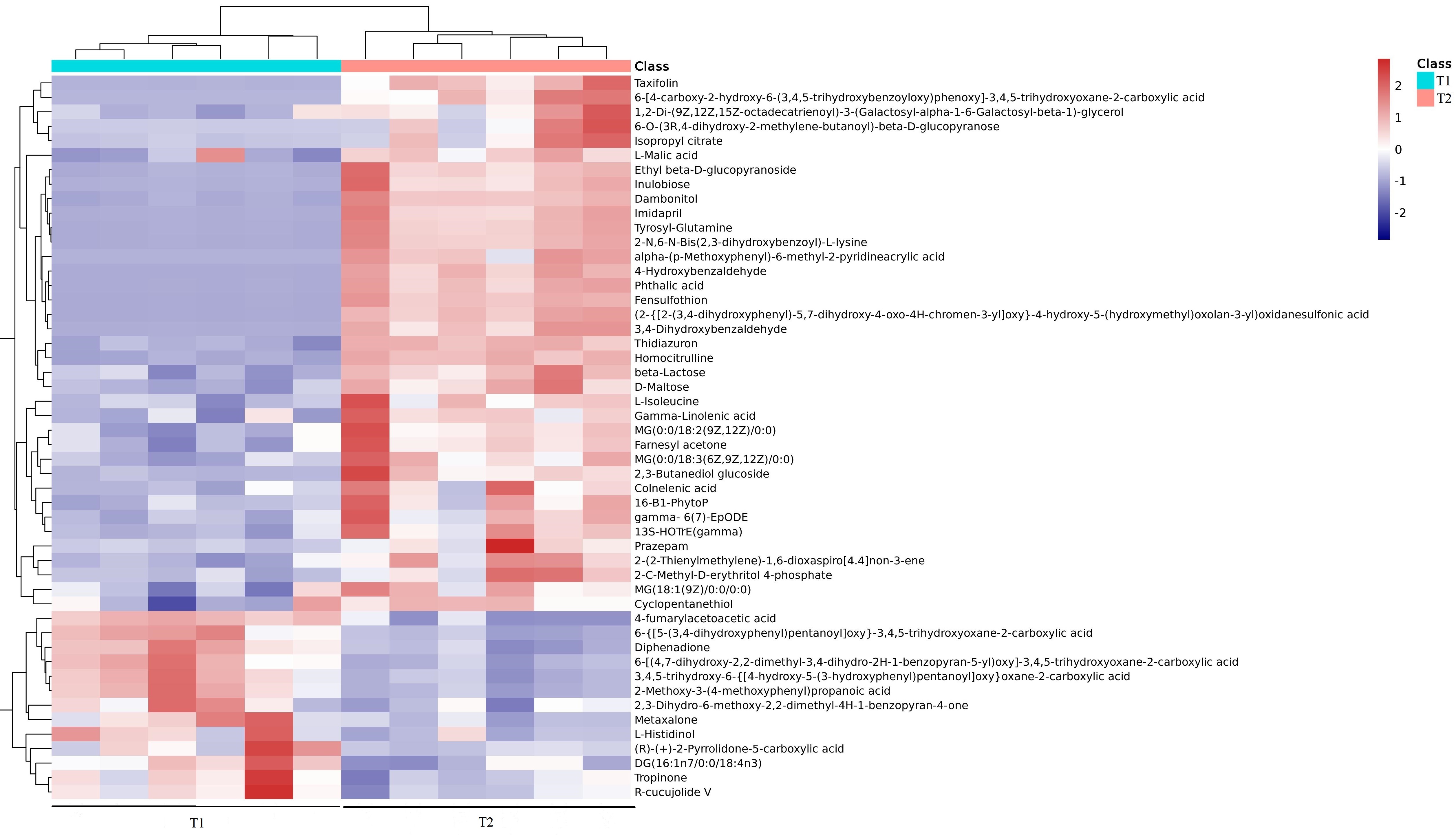


Fig. S6 Heatmap of DAMs


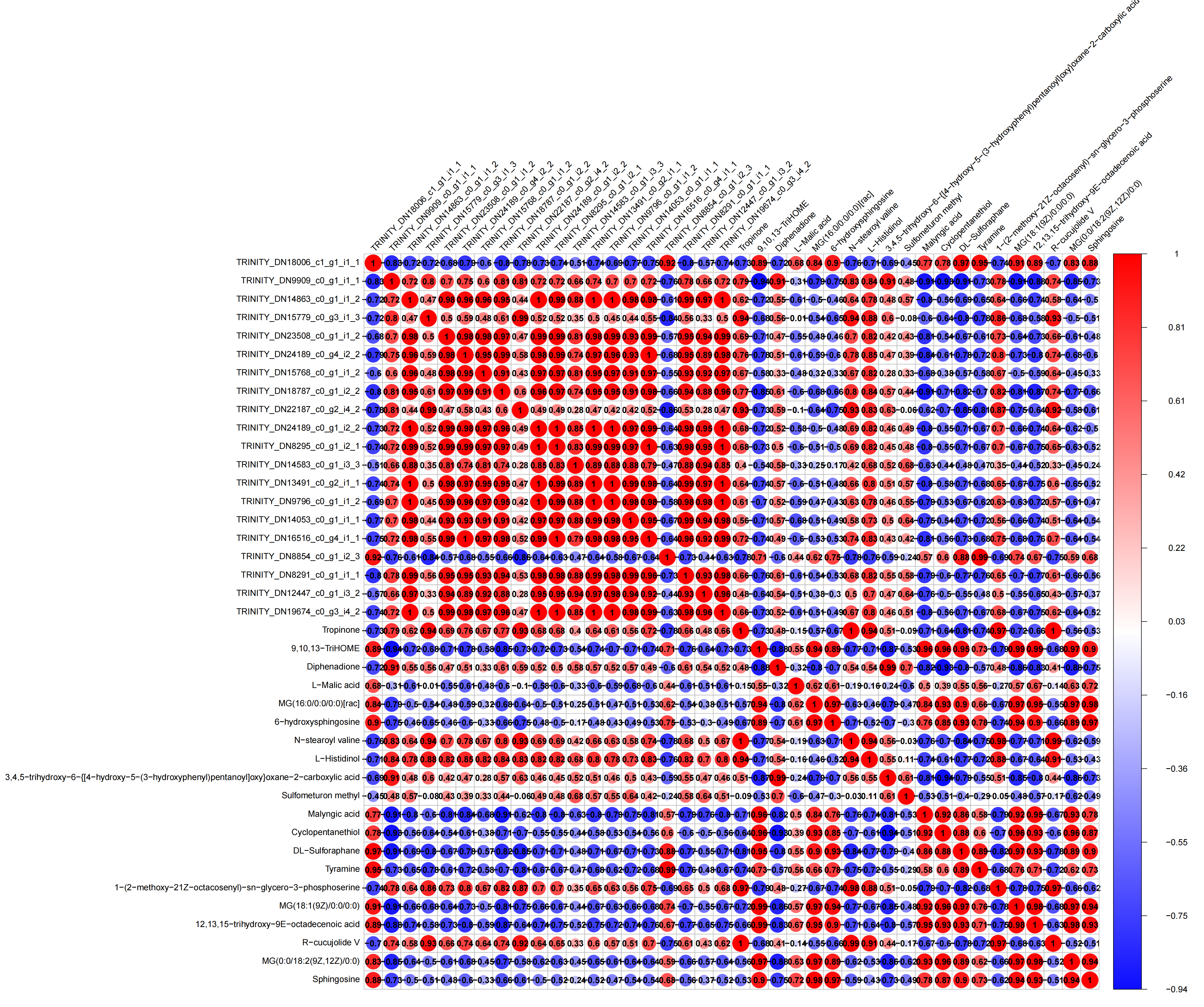


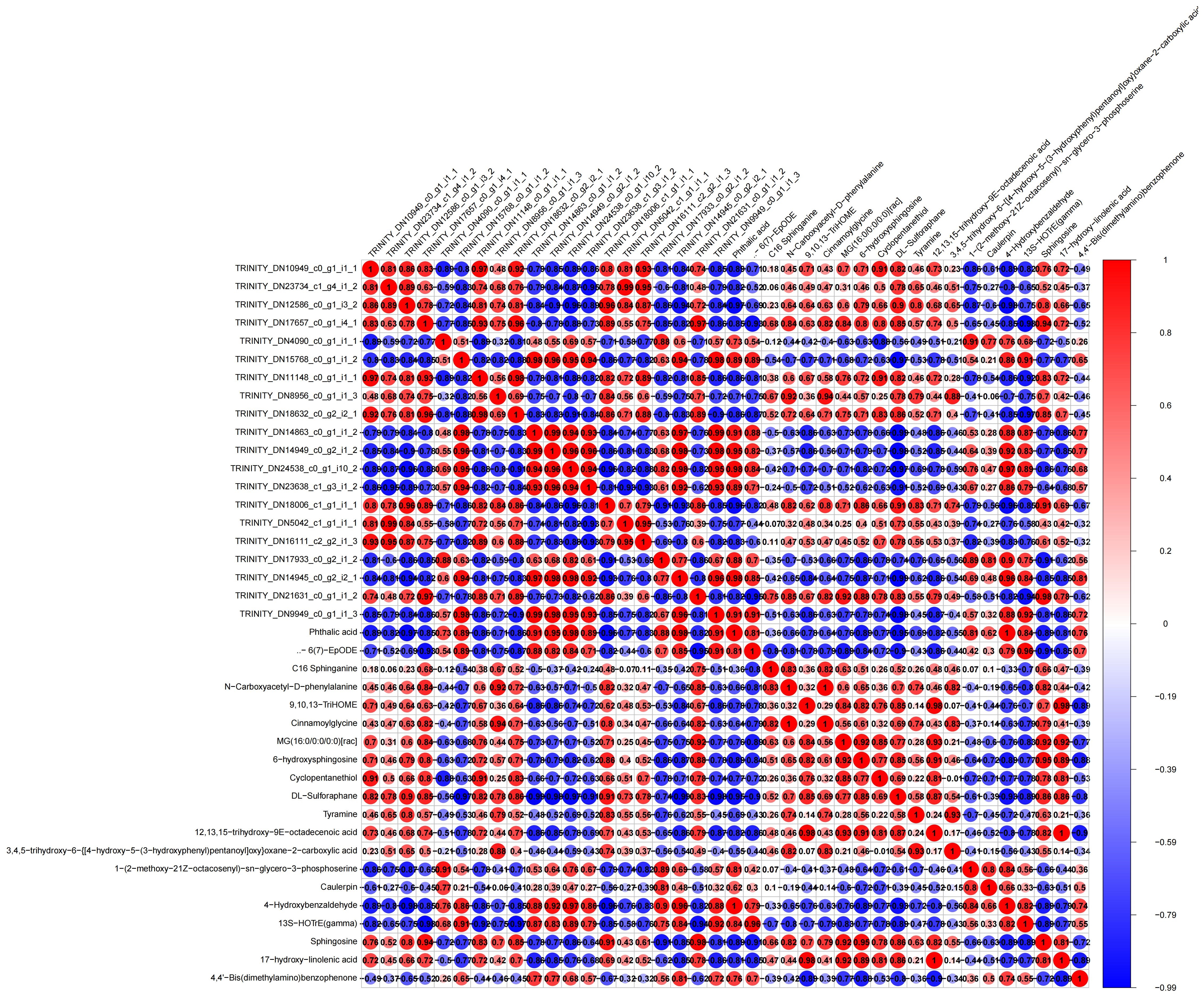
Fig. S7 The correlations of the top 20 transcript and metabolite

Note:(a) T1 vs CK; (b) T2 vs CK. The orange-red in the figure was a positive correlation, and the blue was a negative correlation. The darker the color, the greater the correlation. The size of the circle represented the size of the correlation, and the larger the circle, the greater the correlation.


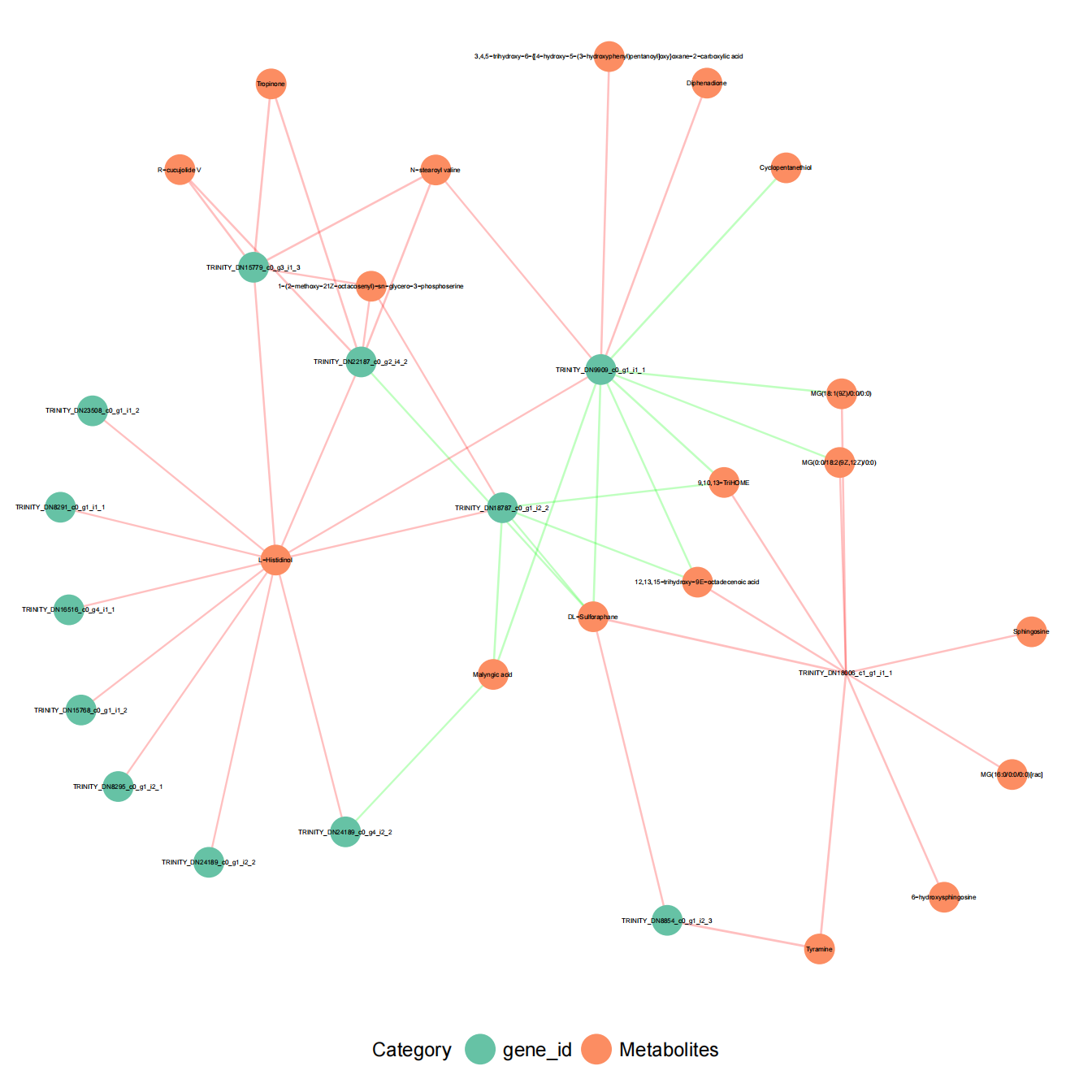


Fig. S8 Correlation network diagram of DEGs and DAMs

Note: The correlations between genes and metabolites were calculated based on the Pearson correlation analysis, and the network diagram was constructed for the relationship pairs for which the p value was <= 0.05. The red line represents a positive correlation, and the green line represents a negative correlation. The thickness of the line represents the strength of the correlation coefficient.


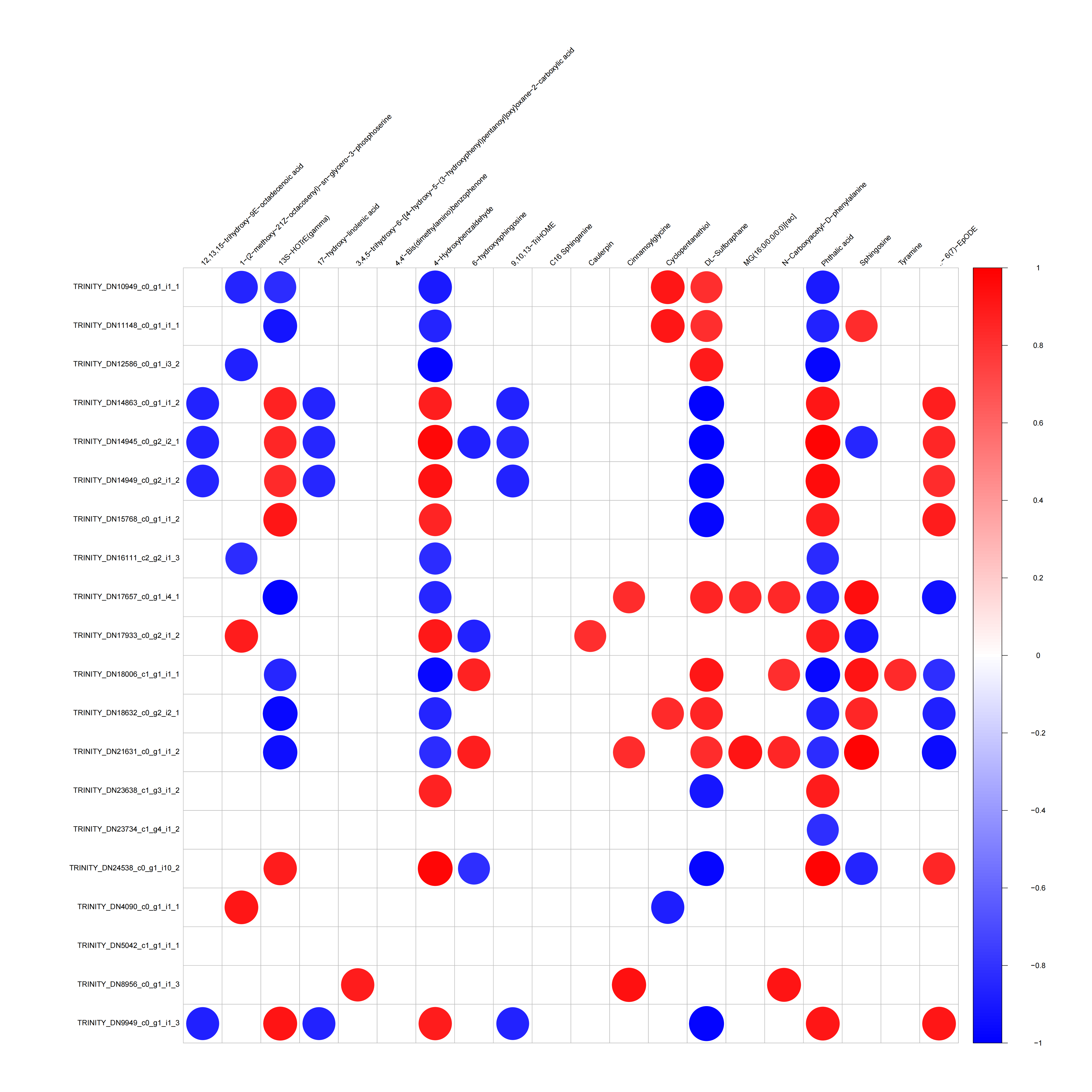


Fig. S9 Correlation analysis of DEGs and DAMs

Note: Each row corresponds to a DEG, and each column corresponds to a DAM. The orange‒red colour indicates a positive correlation, and the blue colour indicates a negative correlation. The darker the colour is, the greater the correlation. The size of the circle represents the size of the correlation, and the larger the circle is, the greater the correlation.
