# Supplementary material for: Responses of transcriptome and metabolome in the roots of Pugionium cornutum (L.) Gaertn to exogenously applied phthalic acid
Source: BMC Plant Biol. 2022 Nov 17;22:535. doi: 10.1186/s12870-022-03927-9 (PMC9670373; doi:10.1186/s12870-022-03927-9)
Supplement: Supplementary file 2 — Additional file 2: Table S1. Primer sequences. Table S2. RNA-seq data statistics. Table S3. The KEGG Enrichment analysis of DEGs between CK and T1. Table S4. The KEGG Enrichment analysis of DEGs between CK and T2. Table S5. The classes of metabolites. Table S6. The positive ion modes of metabolites between T1 and T2. Table S7. The negative ion modes of metabolites between T1 and T2. [file 12870_2022_3927_MOESM2_ESM.doc]

Table S1 Primer sequences

| Primer name | Sequences (5’→3’) | |
| --- | --- | --- |
|  | Left primer | Right primer |
| *ß-Actin* | CATCCCTTGGAGGATCAAGTATT | CCTCTTGTTGCTTTCGGTTTC |
| TRINITY_DN15768_c0_g1_i1_2 | TGGAGAGAAGAATGGGGAAA | AAGCAAAACGAACCACAACC |
| TRINITY_DN15779_c0_g3_i1_3 | GCGGAGGAGTTGAAGAAGTG | GCGGTGCAGTAGACAAGGAT |
| TRINITY_DN9909_c0_g1_i1_1 | CCACCACTCCCCAGCTATAA | TCCCATTTTTCACCATCCAT |
| TRINITY_DN11148_c0_g1_i1_1 | TGCGTTTGATCTCAGCTGTT | AGTAGCAACGACGACGGAAT |
| TRINITY_DN12586_c0_g1_i3_2 | AATGGCGGAGACTGGTACTG | GCCTCCCCTCCAAACTAGAC |
| TRINITY_DN16111_c2_g2_i1_3 | GTTGACGTTGGTGGTGGTTT | CCACCTCCATAACCAGATCC |
| TRINITY_DN17657_c0_g1_i4_1 | CCGTCACGGCTCTTATCTTC | CTCACACCGTCATGAACACC |
| TRINITY_DN14863_c0_g1_i1_2 | ACGTCTTTGATCCGTTTTCG | TTTTCAGTCCTGGCAAGTCC |
| TRINITY_DN14949_c0_g2_i1_2 | TGTTCGATTTCTTGGGGAAG | TCTCACCATCTTCGCCTTCT |
| TRINITY_DN23638_c1_g3_i1_2 | TCTTCACCACATCCCAGTGA | TTAAGAAATTGGGCCGTGAG |

Table S2 RNA-seq data statistics

| Sample | Raw Reads  (M) | Raw Bases  (G) | Clean Reads  (M) | Clean Bases  (G) | Valid Bases  (%) | Q30  (%) | GC  (%) |
| --- | --- | --- | --- | --- | --- | --- | --- |
| CK_1 | 47.49 | 7.12 | 46.94 | 6.79 | 95.35 | 95.28 | 45.37 |
| CK_2 | 50.63 | 7.59 | 50.19 | 7.31 | 96.24 | 96.76 | 45.17 |
| CK_3 | 40.38 | 6.06 | 39.87 | 5.78 | 95.38 | 96.48 | 45.38 |
| T1_1 | 44.46 | 6.67 | 44.08 | 6.40 | 95.91 | 96.15 | 45.28 |
| T1_2 | 48.50 | 7.28 | 48.07 | 6.94 | 95.44 | 96.73 | 45.41 |
| T1_3 | 47.93 | 7.19 | 47.66 | 6.89 | 95.83 | 97.25 | 45.53 |
| T2_1 | 51.61 | 7.74 | 51.17 | 7.42 | 95.82 | 96.47 | 45.51 |
| T2_2 | 40.02 | 6.00 | 39.52 | 5.73 | 95.46 | 96.48 | 45.65 |
| T2_3 | 50.07 | 7.51 | 49.63 | 7.13 | 94.94 | 96.01 | 45.76 |
| Total | 421.09 | 63.16 | 417.13 | 60.39 |  |  |  |

Table S3 The KEGG Enrichment analysis of DEGs between CK and T1

Table S4 The KEGG Enrichment analysis of DEGs between CK and T2

Table S5 The classes of metabolites

**T1 VS T2**

| **Metabolites** | **Compound ID** | **Super Class** | **Class** | **Sub Class** |
| --- | --- | --- | --- | --- |
| Phthalic acid | HMDB0002107 | Benzenoids | Benzene and substituted derivatives | Benzoic acids and derivatives |
| γ- 6(7)-EpODE | 36042 | Unclassified | Unclassified | Unclassified |
| 4-Hydroxybenzaldehyde | HMDB0011718 | Organic oxygen compounds | Organooxygen compounds | Carbonyl compounds |
| Dambonitol | HMDB0033942 | Organic oxygen compounds | Organooxygen compounds | Alcohols and polyols |
| Diphenadione | 72476 | Unclassified | Unclassified | Unclassified |
| Tropinone | 64425 | Unclassified | Unclassified | Unclassified |
| (2-{[2-(3,4-dihydroxyphenyl)-5,7-dihydroxy-4-oxo-4H-chromen-3-yl]oxy}-4-hydroxy-5-(hydroxymethyl)oxolan-3-yl)oxidanesulfonic acid | HMDB0132731 | Phenylpropanoids and polyketides | Flavonoids | Flavonoid glycosides |
| 3,4,5-trihydroxy-6-{[4-hydroxy-5-(3-hydroxyphenyl)pentanoyl]oxy}oxane-2-carboxylic acid | HMDB0127755 | Organic oxygen compounds | Organooxygen compounds | Carbohydrates and carbohydrate conjugates |
| 6-O-(3R,4-dihydroxy-2-methylene-butanoyl)-beta-D-glucopyranose | LMSL05000003 | Unclassified | Unclassified | Unclassified |
| 3,4-Dihydroxybenzaldehyde | HMDB0059965 | Organic oxygen compounds | Organooxygen compounds | Carbonyl compounds |
| 13S-HOTrE(gamma) | 35514 | Lipids and lipid-like molecules | Fatty Acyls | Octadecanoids |
| L-Malic acid | 45931 | Organic acids and derivatives | Hydroxy acids and derivatives | Beta hydroxy acids and derivatives |
| Tyrosyl-Glutamine | HMDB0029103 | Organic acids and derivatives | Carboxylic acids and derivatives | Amino acids, peptides, and analogues |
| Ethyl beta-D-glucopyranoside | HMDB0029968 | Organic oxygen compounds | Organooxygen compounds | Carbohydrates and carbohydrate conjugates |
| MG(18:1(9Z)/0:0/0:0) | HMDB0011567 | Lipids and lipid-like molecules | Glycerolipids | Monoradylglycerols |
| MG(0:0/18:3(6Z,9Z,12Z)/0:0) | 62323 | Lipids and lipid-like molecules | Fatty Acyls | Lineolic acids and derivatives |
| Prazepam | HMDB0015527 | Organoheterocyclic compounds | Benzodiazepines | 1,4-benzodiazepines |
| Fensulfothion | 70114 | Unclassified | Unclassified | Unclassified |
| Thidiazuron | 72579 | Unclassified | Unclassified | Unclassified |
| Cyclopentanethiol | HMDB0039771 | Organosulfur compounds | Thiols | Alkylthiols |
| Imidapril | HMDB0041907 | Organic acids and derivatives | Carboxylic acids and derivatives | Amino acids, peptides, and analogues |
| 2,3-Butanediol glucoside | HMDB0040822 | Organic oxygen compounds | Organooxygen compounds | Carbohydrates and carbohydrate conjugates |
| alpha-(p-Methoxyphenyl)-6-methyl-2-pyridineacrylic acid | 70613 | Unclassified | Unclassified | Unclassified |
| MG(0:0/18:2(9Z,12Z)/0:0) | HMDB0011538 | Lipids and lipid-like molecules | Fatty Acyls | Lineolic acids and derivatives |
| 2-(2-Thienylmethylene)-1,6-dioxaspiro[4.4]non-3-ene | HMDB0034857 | Organic oxygen compounds | Organooxygen compounds | Ethers |
| L-Histidinol | HMDB0003431 | Organic nitrogen compounds | Organonitrogen compounds | Amines |
| L-Isoleucine | HMDB0000172 | Organic acids and derivatives | Carboxylic acids and derivatives | Amino acids, peptides, and analogues |
| Inulobiose | HMDB0029898 | Organic oxygen compounds | Organooxygen compounds | Carbohydrates and carbohydrate conjugates |
| Homocitrulline | HMDB0000679 | Organic acids and derivatives | Carboxylic acids and derivatives | Amino acids, peptides, and analogues |
| 2-C-Methyl-D-erythritol 4-phosphate | 64013 | Unclassified | Unclassified | Unclassified |
| 6-{[5-(3,4-dihydroxyphenyl)pentanoyl]oxy}-3,4,5-trihydroxyoxane-2-carboxylic acid | HMDB0127759 | Organic oxygen compounds | Organooxygen compounds | Carbohydrates and carbohydrate conjugates |
| Gamma-Linolenic acid | HMDB0003073 | Lipids and lipid-like molecules | Fatty Acyls | Lineolic acids and derivatives |
| 1,2-Di-(9Z,12Z,15Z-octadecatrienoyl)-3-(Galactosyl-alpha-1-6-Galactosyl-beta-1)-glycerol | HMDB0011127 | Lipids and lipid-like molecules | Glycerolipids | Glycosylglycerols |
| Taxifolin | HMDB0125371 | Phenylpropanoids and polyketides | Flavonoids | Flavans |
| beta-Lactose | HMDB0041627 | Organic oxygen compounds | Organooxygen compounds | Carbohydrates and carbohydrate conjugates |
| Isopropyl citrate | 88388 | Unclassified | Unclassified | Unclassified |
| 2,3-Dihydro-6-methoxy-2,2-dimethyl-4H-1-benzopyran-4-one | HMDB0041410 | Organoheterocyclic compounds | Benzopyrans | 1-benzopyrans |
| D-Maltose | HMDB0000163 | Organic oxygen compounds | Organooxygen compounds | Carbohydrates and carbohydrate conjugates |
| DG(16:1n7/0:0/18:4n3) | HMDB0056172 | Lipids and lipid-like molecules | Fatty Acyls | Lineolic acids and derivatives |
| 6-[(4,7-dihydroxy-2,2-dimethyl-3,4-dihydro-2H-1-benzopyran-5-yl)oxy]-3,4,5-trihydroxyoxane-2-carboxylic acid | HMDB0125821 | Organic oxygen compounds | Organooxygen compounds | Carbohydrates and carbohydrate conjugates |
| 2-N,6-N-Bis(2,3-dihydroxybenzoyl)-L-lysine | 66403 | Unclassified | Unclassified | Unclassified |
| 4-fumarylacetoacetic acid | LMFA01170066 | Organic acids and derivatives | Keto acids and derivatives | Medium-chain keto acids and derivatives |
| 2-Methoxy-3-(4-methoxyphenyl)propanoic acid | HMDB0039428 | Phenylpropanoids and polyketides | Phenylpropanoic acids | Unclassified |
| Farnesyl acetone | 265036 | Unclassified | Unclassified | Unclassified |
| Colnelenic acid | LMFA10000002 | Lipids and lipid-like molecules | Fatty Acyls | Fatty acids and conjugates |
| 6-[4-carboxy-2-hydroxy-6-(3,4,5-trihydroxybenzoyloxy)phenoxy]-3,4,5-trihydroxyoxane-2-carboxylic acid | HMDB0128328 | Phenylpropanoids and polyketides | Tannins | Hydrolyzable tannins |
| Metaxalone | HMDB0014798 | Benzenoids | Phenol ethers | Unclassified |
| (R)-(+)-2-Pyrrolidone-5-carboxylic acid | 63632 | Unclassified | Unclassified | Unclassified |
| R-cucujolide V | LMFA07040048 | Lipids and lipid-like molecules | Fatty Acyls | Fatty esters |
| 16-B1-PhytoP | LMFA02030004 | Lipids and lipid-like molecules | Fatty Acyls | Octadecanoids |
| 4-hydroxy-butyric acid | LMFA01050006 | Lipids and lipid-like molecules | Fatty Acyls | Fatty acids and conjugates |
| Alpha-Linolenic acid | HMDB0001388 | Lipids and lipid-like molecules | Fatty Acyls | Lineolic acids and derivatives |
| Cellulose, microcrystalline | HMDB0032197 | Organic oxygen compounds | Organooxygen compounds | Carbohydrates and carbohydrate conjugates |
| Benzyl b-L-arabinopyranoside | 96293 | Unclassified | Unclassified | Unclassified |
| {3,8,15-trihydroxy-16,17-dimethoxy-9-oxotricyclo[12.3.1.1²,⁶]nonadeca-1(17),2,4,6(19),14(18),15-hexaen-7-yl}oxidanesulfonic acid | HMDB0133716 | Phenylpropanoids and polyketides | Diarylheptanoids | Cyclic diarylheptanoids |
| 6-[5-({[3,5-dihydroxy-2-(hydroxymethyl)-6-(3,4,5-trihydroxybenzoyloxy)oxan-4-yl]oxy}carbonyl)-2,3-dihydroxyphenoxy]-3,4,5-trihydroxyoxane-2-carboxylic acid | HMDB0133127 | Phenylpropanoids and polyketides | Tannins | Hydrolyzable tannins |
| S-(1,2-DICARBOXYETHYL)GLUTATHIONE | 44314 | Unclassified | Unclassified | Unclassified |
| 3-(3,4-dihydroxy-5-methoxyphenyl)oxirane-2-carboxylic acid | HMDB0125587 | Benzenoids | Phenols | Methoxyphenols |
| 3-(2H-1,3-benzodioxol-5-yl)-3-oxopropanoic acid | HMDB0129376 | Organoheterocyclic compounds | Benzodioxoles | Unclassified |
| Starch acetate | 86570 | Organic acids and derivatives | Carboxylic acids and derivatives | Tricarboxylic acids and derivatives |
| Phenylalanylproline | HMDB0011177 | Organic acids and derivatives | Carboxylic acids and derivatives | Amino acids, peptides, and analogues |
| Garcinia lactone dibutyl ester | HMDB0040462 | Organic acids and derivatives | Carboxylic acids and derivatives | Tricarboxylic acids and derivatives |
| 5a,6a-Epoxy-7E-megastigmene-3a,9e-diol 3-glucoside | HMDB0031676 | Organic oxygen compounds | Organooxygen compounds | Carbohydrates and carbohydrate conjugates |
| beta-Glucogallin | HMDB0038728 | Phenylpropanoids and polyketides | Tannins | Unclassified |
| 7-O-Methyltectorigenin 4'-O-gentiobioside | LMPK12050377 | Lipids and lipid-like molecules | Polyketides | Flavonoids |
| Ascorbalamic acid | HMDB0029944 | Organic acids and derivatives | Carboxylic acids and derivatives | Amino acids, peptides, and analogues |
| Alpha-D-Glucopyranoside | HMDB0061922 | Organic oxygen compounds | Organooxygen compounds | Carbohydrates and carbohydrate conjugates |
| 10-Acetoxyligustroside | HMDB0035217 | Lipids and lipid-like molecules | Prenol lipids | Terpene glycosides |
| Behenic acid | HMDB0000944 | Lipids and lipid-like molecules | Fatty Acyls | Fatty acids and conjugates |
| 3,11,12-Trihydroxy-1(10)-spirovetiven-2-one | HMDB0038154 | Lipids and lipid-like molecules | Prenol lipids | Sesquiterpenoids |
| Threonic acid | HMDB0000943 | Organic oxygen compounds | Organooxygen compounds | Carbohydrates and carbohydrate conjugates |
| Araliacerebroside | HMDB0033621 | Lipids and lipid-like molecules | Sphingolipids | Glycosphingolipids |
| Guanine | HMDB0000132 | Organoheterocyclic compounds | Imidazopyrimidines | Purines and purine derivatives |
| 15-hydroxy stearic acid | LMFA02000133 | Lipids and lipid-like molecules | Fatty Acyls | Octadecanoids |
| 9,12,15-Octadecatrien-1-ol | LMFA05000216 | Lipids and lipid-like molecules | Fatty Acyls | Fatty alcohols |
| Convicine | 66969 | Unclassified | Unclassified | Unclassified |
| 11-Hydroxy-9-tridecenoic acid | HMDB0035881 | Lipids and lipid-like molecules | Fatty Acyls | Fatty acids and conjugates |
| (-)-Wikstromol | HMDB0030598 | Lignans, neolignans and related compounds | Furanoid lignans | Tetrahydrofuran lignans |
| Uridine | HMDB0000296 | Nucleosides, nucleotides, and analogues | Pyrimidine nucleosides | Unclassified |
| Sodium folinate | 71887 | Unclassified | Unclassified | Unclassified |
| L-Valine | HMDB0000883 | Organic acids and derivatives | Carboxylic acids and derivatives | Amino acids, peptides, and analogues |
| Bilastine | HMDB0240232 | Organoheterocyclic compounds | Benzimidazoles | Unclassified |
| 2,5-Dioxopentanoate | HMDB0060365 | Organic acids and derivatives | Keto acids and derivatives | Short-chain keto acids and derivatives |
| Aspartame | HMDB0001894 | Organic acids and derivatives | Carboxylic acids and derivatives | Amino acids, peptides, and analogues |
| 6-{[3-(2H-1,3-benzodioxol-5-yl)-3-oxopropanoyl]oxy}-3,4,5-trihydroxyoxane-2-carboxylic acid | HMDB0129411 | Organic oxygen compounds | Organooxygen compounds | Carbohydrates and carbohydrate conjugates |
| Umbelliferone | HMDB0029865 | Phenylpropanoids and polyketides | Coumarins and derivatives | Hydroxycoumarins |
| 5-Hydroxyflavone | HMDB0040556 | Phenylpropanoids and polyketides | Flavonoids | Flavonoid glycosides |
| cis-Resveratrol 4'-sulfate | HMDB0041714 | Phenylpropanoids and polyketides | Stilbenes | Unclassified |
| 1,2,3,4-Tetramethoxy-5-(2-propenyl)benzene | 89678 | Benzenoids | Phenol ethers | Anisoles |
| 9,12,15-octadecatrienal | LMFA06000102 | Lipids and lipid-like molecules | Fatty Acyls | Fatty aldehydes |
| 11-dehydro-TXB2-d4 | 45968 | Unclassified | Unclassified | Unclassified |
| Galabiose | 86577 | Organic oxygen compounds | Organooxygen compounds | Carbohydrates and carbohydrate conjugates |
| Monoethylhexyl phthalic acid | HMDB0013248 | Benzenoids | Benzene and substituted derivatives | Benzoic acids and derivatives |
| Azidocillin | 85583 | Organoheterocyclic compounds | Lactams | Beta lactams |
| 2-(2-Phenylacetoxy)propionylglycine | HMDB0059732 | Organic acids and derivatives | Carboxylic acids and derivatives | Amino acids, peptides, and analogues |
| Xanthosine | HMDB0000299 | Nucleosides, nucleotides, and analogues | Purine nucleosides | Unclassified |
| O-Desmethyltramadol glucuronide | HMDB0060856 | Organic oxygen compounds | Organooxygen compounds | Carbohydrates and carbohydrate conjugates |
| 5-(2'-Carboxyethyl)-4,6-Dihydroxypicolinate | HMDB0006794 | Organoheterocyclic compounds | Pyridines and derivatives | Hydropyridines |
| (±)-(Z)-2-(5-Tetradecenyl)cyclobutanone | HMDB0037543 | Organic oxygen compounds | Organooxygen compounds | Carbonyl compounds |
| Lusitanicoside | HMDB0034120 | Organic oxygen compounds | Organooxygen compounds | Carbohydrates and carbohydrate conjugates |
| (3S,5R,6S,7E,9x)-7-Megastigmene-3,6,9-triol 9-glucoside | LMFA13010057 | Lipids and lipid-like molecules | Fatty Acyls | Fatty acyl glycosides |
| (2-Chlorophenyl)diphenylmethane | 1898 | Unclassified | Unclassified | Unclassified |
| N-(16,16-dimethy-5Z,8Z,11Z,14Z-docosatetraenoyl)-ethanolamine | LMFA08040018 | Lipids and lipid-like molecules | Fatty Acyls | Fatty amides |
| Uracil | HMDB0000300 | Organoheterocyclic compounds | Diazines | Pyrimidines and pyrimidine derivatives |
| 17-hydroxy stearic acid | LMFA02000135 | Lipids and lipid-like molecules | Fatty Acyls | Octadecanoids |
| N-Phenylacetylglutamic acid | HMDB0059772 | Organic acids and derivatives | Carboxylic acids and derivatives | Amino acids, peptides, and analogues |
| Polidocanol | 69582 | Unclassified | Unclassified | Unclassified |
| 9S-HpOTrE | LMFA02000018 | Lipids and lipid-like molecules | Fatty Acyls | Octadecanoids |
| Pipermethystine | HMDB0033486 | Organoheterocyclic compounds | Pyridines and derivatives | Hydropyridines |
| Methyl salicylate O-[rhamnosyl-(1->6)-glucoside] | HMDB0033138 | Organic oxygen compounds | Organooxygen compounds | Carbohydrates and carbohydrate conjugates |
| 8Z-decen-4,6-diynoic acid | LMFA01050421 | Lipids and lipid-like molecules | Fatty Acyls | Fatty Acids and Conjugates |
| 1-O-(3R,4-dihydroxy-2-methylene-butanoyl)-beta-D-glucopyranose | LMFA13010038 | Lipids and lipid-like molecules | Fatty Acyls | Fatty acyl glycosides |
| 1,6-Digalloyl-beta-D-glucopyranose | HMDB0039179 | Phenylpropanoids and polyketides | Tannins | Unclassified |
| Maltotriose | HMDB0001262 | Organic oxygen compounds | Organooxygen compounds | Carbohydrates and carbohydrate conjugates |
| Alhagidin | 52865 | Lipids and lipid-like molecules | Polyketides | Flavonoids |
| 9-hydroxy-7E-Nonene-3,5-diynoic acid | LMFA01030721 | Lipids and lipid-like molecules | Fatty Acyls | Fatty Acids and Conjugates |
| 15(S)-15-methyl PGF2α isopropyl ester | 64688 | Unclassified | Unclassified | Unclassified |
| 3-Hydroxybutyric acid | HMDB0000357 | Organic acids and derivatives | Hydroxy acids and derivatives | Beta hydroxy acids and derivatives |
| L-Lyxonate | HMDB0060255 | Organic oxygen compounds | Organooxygen compounds | Carbohydrates and carbohydrate conjugates |
| 1-NBD-decanoyl-2-decanoyl-sn-Glycerol | 62997 | Unclassified | Unclassified | Unclassified |
| beta-D-Xylopyranosyl-(1->5)-alpha-L-arabinofuranosyl-(1->5)-L-arabinose | HMDB0038860 | Organic oxygen compounds | Organooxygen compounds | Carbohydrates and carbohydrate conjugates |
| 6-Thioinosinic acid | HMDB0060791 | Nucleosides, nucleotides, and analogues | Purine nucleosides | Unclassified |
| Cyclodopa glucoside | HMDB0029833 | Organic oxygen compounds | Organooxygen compounds | Carbohydrates and carbohydrate conjugates |
| Isoleucyl-Glutamine | HMDB0028905 | Organic acids and derivatives | Carboxylic acids and derivatives | Amino acids, peptides, and analogues |
| 17-HOME(9Z) | LMFA02000180 | Lipids and lipid-like molecules | Fatty Acyls | Octadecanoids |
| Nonadecandioic acid | LMFA01170135 | Lipids and lipid-like molecules | Fatty Acyls | Fatty Acids and Conjugates |
| 2-benzyl-2-hydroxybutanedioic acid | HMDB0142172 | Phenylpropanoids and polyketides | Phenylpropanoic acids | Unclassified |
| 3,4,5-trihydroxy-6-(2-hydroxyethoxy)oxane-2-carboxylic acid | HMDB0131294 | Organic oxygen compounds | Organooxygen compounds | Carbohydrates and carbohydrate conjugates |
| gamma-Carboxyglutamic acid | HMDB0041900 | Organic acids and derivatives | Carboxylic acids and derivatives | Amino acids, peptides, and analogues |
| 8-Hydroxypinoresinol 8-glucoside | HMDB0033281 | Lignans, neolignans and related compounds | Lignan glycosides | Unclassified |
| Biflorin | HMDB0002336 | Organic oxygen compounds | Organooxygen compounds | Carbohydrates and carbohydrate conjugates |
| Linoleic acid | HMDB0000673 | Lipids and lipid-like molecules | Fatty Acyls | Lineolic acids and derivatives |
| 6-[1]-ladderane hexanol | LMFA01140088 | Lipids and lipid-like molecules | Fatty Acyls | Fatty Acids and Conjugates |
| Cilazprilat | 1749 | Unclassified | Unclassified | Unclassified |
| S-(2,5-Dimethyl-3-furanyl) 2-furancarbothioate | HMDB0039585 | Organoheterocyclic compounds | Furans | Furoic acid and derivatives |
| Glutamylvaline | HMDB0028832 | Organic acids and derivatives | Carboxylic acids and derivatives | Amino acids, peptides, and analogues |
| 5-(3-methoxyphenyl)-4-(sulfooxy)pentanoic acid | HMDB0127785 | Lipids and lipid-like molecules | Fatty Acyls | Fatty acids and conjugates |
| 1-Guanidino-1-deoxy-scyllo-inositol 4-phosphate | 64554 | Unclassified | Unclassified | Unclassified |
| N-(4-aminobutyl)-3-(4-hydroxyphenyl)propanimidic acid | HMDB0139525 | Benzenoids | Phenols | 1-hydroxy-2-unsubstituted benzenoids |
| 16-epi-16-D1t-PhytoP | LMFA02030036 | Lipids and lipid-like molecules | Fatty Acyls | Octadecanoids |
| 4-(N-Maleimido)benzyltrimethylammonium iodide | 69630 | Unclassified | Unclassified | Unclassified |
| (±)9-HODE | 35487 | Unclassified | Unclassified | Unclassified |
| 6-[(4,5-dihydroxy-2,2-dimethyl-3,4-dihydro-2H-1-benzopyran-7-yl)oxy]-3,4,5-trihydroxyoxane-2-carboxylic acid | HMDB0125822 | Organic oxygen compounds | Organooxygen compounds | Carbohydrates and carbohydrate conjugates |
| 2,3,4-Trihydroxybenzylhydrazide | 1291 | Unclassified | Unclassified | Unclassified |
| Bifenazate | 72390 | Unclassified | Unclassified | Unclassified |
| Morphine 3,6-diglucuronide | 1360 | Unclassified | Unclassified | Unclassified |
| Olanzapine | HMDB0005012 | Organoheterocyclic compounds | Benzodiazepines | Unclassified |
| Melibiose | HMDB0000048 | Organic oxygen compounds | Organooxygen compounds | Carbohydrates and carbohydrate conjugates |
| 12-Hydroxy-8,10-octadecadienoic acid | HMDB0029998 | Lipids and lipid-like molecules | Fatty Acyls | Lineolic acids and derivatives |
| Latrunculin B | 45288 | Unclassified | Unclassified | Unclassified |
| 4-({2-[(3-{2-[2-(acetyloxy)propan-2-yl]-7-oxo-2H,3H,7H-furo[3,2-g]chromen-6-yl}-1-hydroxy-3-methylbutan-2-yl)sulfanyl]-1-[(carboxymethyl)-C-hydroxycarbonimidoyl]ethyl}-C-hydroxycarbonimidoyl)-2-aminobutanoic acid | HMDB0130306 | Organic acids and derivatives | Carboxylic acids and derivatives | Amino acids, peptides, and analogues |
| (±)-threo-1-(p-Hydroxyphenyl)propylene glycol 4'-glucoside | HMDB0033068 | Organic oxygen compounds | Organooxygen compounds | Carbohydrates and carbohydrate conjugates |
| (±)-Glycerol 1,2-diacetate | HMDB0031712 | Lipids and lipid-like molecules | Glycerolipids | Diradylglycerols |
| Apholate | 73037 | Unclassified | Unclassified | Unclassified |
| 4-Oxo-1-(3-pyridyl)-1-butanone | HMDB0062406 | Organic oxygen compounds | Organooxygen compounds | Carbonyl compounds |
| 3,4,5-trihydroxy-6-({7-oxo-7H-furo[3,2-g]chromen-4-yl}oxy)oxane-2-carboxylic acid | HMDB0129424 | Organic oxygen compounds | Organooxygen compounds | Carbohydrates and carbohydrate conjugates |
| famprofazone | 96167 | Organoheterocyclic compounds | Azoles | Pyrazoles |
| 3-Hydroxychavicol 1-glucoside | 90251 | Organic oxygen compounds | Organooxygen compounds | Carbohydrates and carbohydrate conjugates |
| Arginyl-Proline | HMDB0028717 | Organic acids and derivatives | Carboxylic acids and derivatives | Amino acids, peptides, and analogues |
| 5-Hydroxymethyl-2-furancarboxaldehyde | HMDB0034355 | Organic oxygen compounds | Organooxygen compounds | Carbonyl compounds |
| (3,4,5,6-tetrahydroxyoxan-2-yl)methyl 3-(4-hydroxy-3-methoxyphenyl)prop-2-enoate | HMDB0125101 | Phenylpropanoids and polyketides | Cinnamic acids and derivatives | Hydroxycinnamic acids and derivatives |
| Pirimiphos-methyl | 72211 | Unclassified | Unclassified | Unclassified |
| Trigonelline | HMDB0000875 | Alkaloids and derivatives | Unclassified | Unclassified |
| SB 221284 | 69190 | Unclassified | Unclassified | Unclassified |
| Gibberellin A36 | LMPR0104170019 | Lipids and lipid-like molecules | Prenol lipids | Isoprenoids |
| L-Threonine | HMDB0000167 | Organic acids and derivatives | Carboxylic acids and derivatives | Amino acids, peptides, and analogues |
| Ketophenylbutazone | HMDB0041914 | Benzenoids | Benzene and substituted derivatives | Unclassified |
| 5a,11a-Dehydrooxytetracycline | 72132 | Unclassified | Unclassified | Unclassified |
| Rhamnetin 3-rhamnosyl-(1->3)(4'''-acetylrhamnosyl)(1->6)-galactoside | LMPK12112605 | Lipids and lipid-like molecules | Polyketides | Flavonoids |
| Crotoxyphos | 72730 | Unclassified | Unclassified | Unclassified |
| 10-hydroxy-8E-Decene-2,4,6-triynoic acid | LMFA01030714 | Lipids and lipid-like molecules | Fatty Acyls | Fatty Acids and Conjugates |
| (3,4,5,6-tetrahydroxyoxan-2-yl)methyl 4-hydroxybenzoate | HMDB0125107 | Benzenoids | Benzene and substituted derivatives | Benzoic acids and derivatives |
| S-NEPC | 64656 | Unclassified | Unclassified | Unclassified |
| Flumetover | 68705 | Unclassified | Unclassified | Unclassified |
| Coriandrone C | HMDB0041525 | Phenylpropanoids and polyketides | Isocoumarins and derivatives | Unclassified |
| 2-Hydroxy-6-ketononatrienedioate | 63525 | Unclassified | Unclassified | Unclassified |
| Asymmetric dimethylarginine | HMDB0001539 | Organic acids and derivatives | Carboxylic acids and derivatives | Amino acids, peptides, and analogues |
| 12Z,15Z-heneicosadienoic acid | LMFA01030399 | Lipids and lipid-like molecules | Fatty Acyls | Fatty Acids and Conjugates |
| Levan | HMDB0003539 | Organic oxygen compounds | Organooxygen compounds | Carbohydrates and carbohydrate conjugates |
| 4-Deoxy-beta-D-gluc-4-enuronosyl-(1,3)-N-acetyl-D-galactosamine 4-sulfate | 66269 | Unclassified | Unclassified | Unclassified |
| [1-(4-methoxyphenyl)-3-phenylpropoxy]sulfonic acid | HMDB0135641 | Phenylpropanoids and polyketides | Linear 1,3-diarylpropanoids | Unclassified |
| Propofol glucuronide | HMDB0060933 | Organic oxygen compounds | Organooxygen compounds | Carbohydrates and carbohydrate conjugates |
| Agomelatine | HMDB0015636 | Organic acids and derivatives | Carboxylic acids and derivatives | Carboxylic acid derivatives |
| DIHYDROCELASTROL | 44189 | Unclassified | Unclassified | Unclassified |
| Methyl helianthenoate F glucoside | HMDB0040797 | Lipids and lipid-like molecules | Fatty Acyls | Fatty acyl glycosides |
| 5-Methylchrysene | 72979 | Unclassified | Unclassified | Unclassified |
| N-HFG | 64631 | Unclassified | Unclassified | Unclassified |
| 4-hydroxy-5-[4-hydroxy-3-(sulfooxy)phenyl]pentanoic acid | HMDB0127746 | Lipids and lipid-like molecules | Fatty Acyls | Fatty acids and conjugates |
| Citrusin C | HMDB0038708 | Organic oxygen compounds | Organooxygen compounds | Carbohydrates and carbohydrate conjugates |
| 7,8-Dihydroneopterin | HMDB0002275 | Organoheterocyclic compounds | Pteridines and derivatives | Pterins and derivatives |
| Ritalinic acid | HMDB0042008 | Organic nitrogen compounds | Organonitrogen compounds | Amines |
| Taraxacolide 1-O-b-D-glucopyranoside | HMDB0035610 | Lipids and lipid-like molecules | Prenol lipids | Terpene lactones |
| Adrenoyl ethanolamide | HMDB0013626 | Organic nitrogen compounds | Organonitrogen compounds | Amines |
| 19-Hydroxy-8-O-methyltetrangulol | 63758 | Unclassified | Unclassified | Unclassified |
| S-Phenylmercapturic acid | HMDB0042011 | Organic acids and derivatives | Carboxylic acids and derivatives | Amino acids, peptides, and analogues |
| 5-ethyl-5-methyl-2,4-oxazolidinedione | HMDB0061082 | Organoheterocyclic compounds | Azolidines | Oxazolidines |
| Pibutidine | HMDB0041986 | Organic nitrogen compounds | Organonitrogen compounds | Amines |
| Tanacetol A | HMDB0035722 | Lipids and lipid-like molecules | Prenol lipids | Sesquiterpenoids |
| {6-hydroxy-3-[3-(3-hydroxyphenyl)-3-oxopropyl]-2-methoxyphenyl}oxidanesulfonic acid | HMDB0126077 | Phenylpropanoids and polyketides | Linear 1,3-diarylpropanoids | Chalcones and dihydrochalcones |
| 5alpha-Androstan-3alpha,17beta-diol disulfate | HMDB0094682 | Lipids and lipid-like molecules | Steroids and steroid derivatives | Sulfated steroids |
| 5-Carboxy-2'-deoxyuridine | HMDB0060774 | Nucleosides, nucleotides, and analogues | Pyrimidine nucleosides | Pyrimidine 2'-deoxyribonucleosides |
| Ikarisoside D | LMPK12111724 | Lipids and lipid-like molecules | Polyketides | Flavonoids |
| Valyl-Glutamine | HMDB0029125 | Organic acids and derivatives | Carboxylic acids and derivatives | Amino acids, peptides, and analogues |
| Adrenochrome | 64954 | Organoheterocyclic compounds | Indoles and derivatives | Unclassified |
| Pentostatin | HMDB0014692 | Organoheterocyclic compounds | Imidazodiazepines | Unclassified |
| MEFEXAMIDE | 43468 | Unclassified | Unclassified | Unclassified |
| Elaidic acid | HMDB0000573 | Lipids and lipid-like molecules | Fatty Acyls | Fatty acids and conjugates |
| Aprepitant | HMDB0014811 | Organoheterocyclic compounds | Oxazinanes | Morpholines |
| Vicine | 66974 | Organic oxygen compounds | Organooxygen compounds | Carbohydrates and carbohydrate conjugates |
| Magnesium Sulfate | 85410 | Unclassified | Unclassified | Unclassified |
| N-Succinyl-L-citrulline | 63487 | Unclassified | Unclassified | Unclassified |
| Niazinin A | HMDB0031942 | Organic oxygen compounds | Organooxygen compounds | Carbohydrates and carbohydrate conjugates |
| (1S,2S)-3-oxo-2-pentyl-cyclopentanebutanoic acid | LMFA02010016 | Lipids and lipid-like molecules | Fatty Acyls | Octadecanoids |
| 3-hydroxy-3-(3-hydroxyphenyl)propanoic acid-O-sulphate | HMDB0059967 | Organic acids and derivatives | Hydroxy acids and derivatives | Beta hydroxy acids and derivatives |
| 3,4,5-trihydroxy-6-[(4-hydroxy-3-methylbut-2-enoyl)oxy]oxane-2-carboxylic acid | HMDB0128929 | Organic oxygen compounds | Organooxygen compounds | Carbohydrates and carbohydrate conjugates |
| Apigenin 4'-[feruloyl-(->2)-glucuronyl-(1->2)-glucuronide] | HMDB0038295 | Phenylpropanoids and polyketides | Flavonoids | Flavonoid glycosides |
| phorbol 13-acetate | LMPR0104330003 | Lipids and lipid-like molecules | Prenol lipids | Isoprenoids |
| Ricinine | HMDB0042006 | Organoheterocyclic compounds | Pyridines and derivatives | 3-pyridinecarbonitriles |
| (±)14(15)-EET methyl ester | 62934 | Unclassified | Unclassified | Unclassified |
| AVOCADYNE ACETATE | 43513 | Unclassified | Unclassified | Unclassified |
| 2,4,6-Trithiaheptane | 88519 | Organosulfur compounds | Thioacetals | Dithioacetals |
| 1-Naphthalenesulfonic acid | 71143 | Unclassified | Unclassified | Unclassified |
| 3-Deoxyarabinohexonic acid | HMDB0000346 | Organic acids and derivatives | Hydroxy acids and derivatives | Medium-chain hydroxy acids and derivatives |
| Nelumboside | HMDB0038464 | Phenylpropanoids and polyketides | Flavonoids | Flavonoid glycosides |
| 2-Octenoic acid | HMDB0000392 | Lipids and lipid-like molecules | Fatty Acyls | Fatty acids and conjugates |
| Hydrocinchonine | HMDB0030283 | Alkaloids and derivatives | Cinchona alkaloids | Unclassified |
| 6-{4-[(1E)-3-{4,5-dihydroxy-3-[hydroxy(3,4,5-trihydroxyoxan-2-yl)methyl]-2,6-dioxo-5-[3,4,5-trihydroxy-6-(hydroxymethyl)oxan-2-yl]cyclohex-3-en-1-yl}-3-oxoprop-1-en-1-yl]phenoxy}-3,4,5-trihydroxyoxane-2-carboxylic acid | HMDB0128285 | Organic oxygen compounds | Organooxygen compounds | Carbohydrates and carbohydrate conjugates |
| Diphemanil Methylsulfate | 85422 | Unclassified | Unclassified | Unclassified |
| Fludarabine phosphate | 675 | Nucleosides, nucleotides, and analogues | Purine nucleotides | Purine ribonucleotides |
| (1S,2R,4R)-p-Menth-8-ene-2,10-diol 2-glucoside | HMDB0039055 | Lipids and lipid-like molecules | Prenol lipids | Terpene glycosides |
| Methysergide | HMDB0014392 | Alkaloids and derivatives | Ergoline and derivatives | Lysergic acids and derivatives |
| 6-({3,7-dihydroxy-2-[3-hydroxy-10-(4-hydroxy-3-methoxyphenyl)-2-oxo-4-oxatricyclo[4.3.1.0³,⁷]decan-8-yl]-4-oxo-3,4-dihydro-2H-1-benzopyran-5-yl}oxy)-3,4,5-trihydroxyoxane-2-carboxylic acid | HMDB0128741 | Phenylpropanoids and polyketides | Flavonoids | Flavans |
| N-dodecanoyl-L-Homoserine lactone-3-hydrazone-fluorescein | 64901 | Unclassified | Unclassified | Unclassified |
| desmethylastemizole | HMDB0061013 | Organoheterocyclic compounds | Benzimidazoles | Unclassified |
| Acrinathrin | 72216 | Unclassified | Unclassified | Unclassified |
| Sophoraflavanone D | LMPK12140473 | Lipids and lipid-like molecules | Polyketides | Flavonoids |
| 2-Hydroxy-2-ethylsuccinic acid | HMDB0059758 | Lipids and lipid-like molecules | Fatty Acyls | Fatty acids and conjugates |
| Leucyl-Isoleucine | HMDB0028932 | Organic acids and derivatives | Carboxylic acids and derivatives | Amino acids, peptides, and analogues |
| Lysyl-Serine | 85862 | Organic acids and derivatives | Carboxylic acids and derivatives | Amino acids, peptides, and analogues |
| L-Leucine | HMDB0000687 | Organic acids and derivatives | Carboxylic acids and derivatives | Amino acids, peptides, and analogues |
| Pseudomonine | HMDB0041438 | Organic acids and derivatives | Carboxylic acids and derivatives | Amino acids, peptides, and analogues |
| ent-7-F2t-dihomo-IsoP | LMFA03110284 | Lipids and lipid-like molecules | Fatty Acyls | Eicosanoids |
| Schizonepetoside B | 71832 | Unclassified | Unclassified | Unclassified |
| 1,2,3,4-Tetrahydroisoquinoline | HMDB0012489 | Organoheterocyclic compounds | Tetrahydroisoquinolines | Unclassified |
| 5alpha-Cholestane | HMDB0041632 | Lipids and lipid-like molecules | Steroids and steroid derivatives | Cholestane steroids |
| Thiotepa | 43348 | Organic acids and derivatives | Organic thiophosphoric acids and derivatives | Unclassified |
| L-Arginine | HMDB0000517 | Organic acids and derivatives | Carboxylic acids and derivatives | Amino acids, peptides, and analogues |
| [6]-Gingerdiol 5-O-beta-D-glucopyranoside | HMDB0036123 | Lipids and lipid-like molecules | Fatty Acyls | Fatty acyl glycosides |
| Boc-Asn-OPhNO2 | 65720 | Unclassified | Unclassified | Unclassified |
| Pentafluorobenzenesulfonyl fluorescein | 64639 | Unclassified | Unclassified | Unclassified |
| IC202A | LMFA08020182 | Lipids and lipid-like molecules | Fatty Acyls | Fatty amides |
| 3-Hydroxy-carbofuran | HMDB0032775 | Organoheterocyclic compounds | Coumarans | Unclassified |
| 1-(2-Thienyl)-1,2-propanedione | HMDB0040002 | Organic oxygen compounds | Organooxygen compounds | Carbonyl compounds |
| 5-Phenyl-1,3-oxazinane-2,4-dione | HMDB0060400 | Benzenoids | Benzene and substituted derivatives | Unclassified |
| Cyanidin 3-O-[2''-O-(xylosyl) glucoside] 5-O-(6'''-O-malonyl) glucoside | 46961 | Unclassified | Unclassified | Unclassified |
| N6'-Acetylkanamycin-B | 65918 | Unclassified | Unclassified | Unclassified |
| Nicorandil-N-oxide | 1523 | Unclassified | Unclassified | Unclassified |
| 6-{[6-(3,4-dihydroxy-6-methyl-5-oxooxan-2-yl)-5-hydroxy-2-(4-hydroxy-3-methoxyphenyl)-4-oxo-4H-chromen-7-yl]oxy}-3,4,5-trihydroxyoxane-2-carboxylic acid | HMDB0127331 | Phenylpropanoids and polyketides | Flavonoids | Flavonoid glycosides |
| 1-Deoxy-D-xylulose | HMDB0001292 | Organic oxygen compounds | Organooxygen compounds | Carbohydrates and carbohydrate conjugates |
| Hydroxyhexanoycarnitine | LMFA07070072 | Lipids and lipid-like molecules | Fatty Acyls | Fatty acid esters |
| 2-Naphthalenesulfonic acid | 71144 | Unclassified | Unclassified | Unclassified |
| 9(S)-HOTrE | 36030 | Unclassified | Unclassified | Unclassified |
| 6-(5-{2-[4,6-dihydroxy-2-methoxy-3-(3-methylbut-2-en-1-yl)phenyl]acetyl}-2,4-dihydroxyphenoxy)-3,4,5-trihydroxyoxane-2-carboxylic acid | HMDB0125482 | Phenylpropanoids and polyketides | Stilbenes | Stilbene glycosides |
| Dioctyl hexanedioate | HMDB0041619 | Lipids and lipid-like molecules | Fatty Acyls | Fatty alcohol esters |
| 13(S)-HpOTrE | 36052 | Unclassified | Unclassified | Unclassified |
| 9(10)-EpOME | LMFA02000037 | Lipids and lipid-like molecules | Fatty Acyls | Fatty acids and conjugates |
| Tartronate semialdehyde | HMDB0006938 | Organic oxygen compounds | Organooxygen compounds | Carbohydrates and carbohydrate conjugates |
| methyl 15-hydroperoxy-9Z,12Z,16E-octadecatrienoate | 74464 | Lipids and lipid-like molecules | Fatty Acyls | Fatty Acids and Conjugates |
| Brosimacutin G | LMPK12120035 | Lipids and lipid-like molecules | Polyketides | Flavonoids |
| Cer(d18:1/2:0) | LMSP02010014 | Lipids and lipid-like molecules | Sphingolipids | Ceramides |
| Indicaxanthin | HMDB0029386 | Organic acids and derivatives | Carboxylic acids and derivatives | Amino acids, peptides, and analogues |
| Isosorbide-2-glucuronide | 864 | Unclassified | Unclassified | Unclassified |
| 1H-Indole-3-carboxaldehyde | HMDB0029737 | Organoheterocyclic compounds | Indoles and derivatives | Indoles |
| trans-Ferulic acid | HMDB0000954 | Phenylpropanoids and polyketides | Cinnamic acids and derivatives | Hydroxycinnamic acids and derivatives |
| Melledonal A | HMDB0035865 | Lipids and lipid-like molecules | Prenol lipids | Sesquiterpenoids |
| CAY10512 | 45185 | Unclassified | Unclassified | Unclassified |
| 2,3-Dihydroxy-3-methylpentanoic acid | LMFA01050469 | Lipids and lipid-like molecules | Fatty Acyls | Fatty acids and conjugates |
| 3,3'-Thiobispropanoic acid | HMDB0031162 | Organic acids and derivatives | Carboxylic acids and derivatives | Dicarboxylic acids and derivatives |
| Neriantogenin | HMDB0030044 | Lipids and lipid-like molecules | Steroids and steroid derivatives | Steroid lactones |
| Acarbose (component 1) | 769 | Unclassified | Unclassified | Unclassified |

**T1 VS CK**

| **Metabolites** | **Compound ID** | **Super Class** | **Class** | **Sub Class** |
| --- | --- | --- | --- | --- |
| 9,10,13-TriHOME | HMDB0004710 | Lipids and lipid-like molecules | Fatty Acyls | Fatty acids and conjugates |
| Tropinone | 64425 | Unclassified | Unclassified | Unclassified |
| Cyclopentanethiol | HMDB0039771 | Organosulfur compounds | Thiols | Alkylthiols |
| DL-Sulforaphane | 58318 | Unclassified | Unclassified | Unclassified |
| Malyngic acid | LMFA01050531 | Lipids and lipid-like molecules | Fatty Acyls | Fatty Acids and Conjugates |
| MG(16:0/0:0/0:0)[rac] | 75555 | Lipids and lipid-like molecules | Glycerolipids | Monoradylglycerols |
| 12,13,15-trihydroxy-9E-octadecenoic acid | LMFA01050555 | Lipids and lipid-like molecules | Fatty Acyls | Fatty Acids and Conjugates |
| 6-hydroxysphingosine | 53903 | Lipids and lipid-like molecules | Sphingolipids | Sphingoid bases |
| Diphenadione | 72476 | Unclassified | Unclassified | Unclassified |
| MG(18:1(9Z)/0:0/0:0) | HMDB0011567 | Lipids and lipid-like molecules | Glycerolipids | Monoradylglycerols |
| 17-hydroxy-linolenic acid | LMFA02000239 | Lipids and lipid-like molecules | Fatty Acyls | Octadecanoids |
| 3,4,5-trihydroxy-6-{[4-hydroxy-5-(3-hydroxyphenyl)pentanoyl]oxy}oxane-2-carboxylic acid | HMDB0127755 | Organic oxygen compounds | Organooxygen compounds | Carbohydrates and carbohydrate conjugates |
| Sulfometuron methyl | 68719 | Unclassified | Unclassified | Unclassified |
| L-Malic acid | 45931 | Organic acids and derivatives | Hydroxy acids and derivatives | Beta hydroxy acids and derivatives |
| 4-Isothiocyanato-1-butene | HMDB0033867 | Organosulfur compounds | Isothiocyanates | Unclassified |
| Prazepam | HMDB0015527 | Organoheterocyclic compounds | Benzodiazepines | 1,4-benzodiazepines |
| MG(0:0/18:3(6Z,9Z,12Z)/0:0) | 62323 | Lipids and lipid-like molecules | Fatty Acyls | Lineolic acids and derivatives |
| Sphingosine | HMDB0000252 | Organic nitrogen compounds | Organonitrogen compounds | Amines |
| 16-F1-PhytoP | LMFA02030001 | Lipids and lipid-like molecules | Fatty Acyls | Octadecanoids |
| methyl 12,13-epoxy-9,15-octadecadienoate | 74810 | Lipids and lipid-like molecules | Fatty Acyls | Fatty Acids and Conjugates |
| epothilone D | LMPK04000001 | Lipids and lipid-like molecules | Polyketides | Macrolides and lactone polyketides |
| Thidiazuron | 72579 | Unclassified | Unclassified | Unclassified |
| 8-Hydroxy-4,8-dimethyl-4E,9-decadienoic acid | LMFA01050373 | Lipids and lipid-like molecules | Fatty Acyls | Fatty Acids and Conjugates |
| Tyramine | HMDB0000306 | Benzenoids | Benzene and substituted derivatives | Phenethylamines |
| O-Oxalylhomoserine | 66891 | Unclassified | Unclassified | Unclassified |
| MG(0:0/18:2(9Z,12Z)/0:0) | HMDB0011538 | Lipids and lipid-like molecules | Fatty Acyls | Lineolic acids and derivatives |
| 16-L1-PhytoP | LMFA02030027 | Lipids and lipid-like molecules | Fatty Acyls | Octadecanoids |
| 1-(2-methoxy-21Z-octacosenyl)-sn-glycero-3-phosphoserine | LMGP03060029 | Lipids and lipid-like molecules | Glycerophospholipids | Glycerophosphoserines |
| 6-Formylindolo [3,2-B] carbazole | 43424 | Unclassified | Unclassified | Unclassified |
| 5Z,8Z,11Z,14Z,17Z-octadecapentaenoic acid | LMFA02000349 | Lipids and lipid-like molecules | Fatty Acyls | Octadecanoids |
| Adenine | HMDB0000034 | Organoheterocyclic compounds | Imidazopyrimidines | Purines and purine derivatives |
| L-Histidinol | HMDB0003431 | Organic nitrogen compounds | Organonitrogen compounds | Amines |
| N-stearoyl valine | LMFA08020122 | Lipids and lipid-like molecules | Fatty Acyls | Fatty amides |
| DG(16:1n7/0:0/18:4n3) | HMDB0056172 | Lipids and lipid-like molecules | Fatty Acyls | Lineolic acids and derivatives |
| LysoPA(0:0/18:2(9Z,12Z)) | HMDB0007852 | Lipids and lipid-like molecules | Glycerophospholipids | Glycerophosphates |
| 2,3-Dihydro-6-methoxy-2,2-dimethyl-4H-1-benzopyran-4-one | HMDB0041410 | Organoheterocyclic compounds | Benzopyrans | 1-benzopyrans |
| 5-Methoxyindoleacetate | HMDB0004096 | Organoheterocyclic compounds | Indoles and derivatives | Indolyl carboxylic acids and derivatives |
| 2-(2-Thienylmethylene)-1,6-dioxaspiro[4.4]non-3-ene | HMDB0034857 | Organic oxygen compounds | Organooxygen compounds | Ethers |
| PC(18:1(9E)/0:0)[U] | 46689 | Unclassified | Unclassified | Unclassified |
| 3,4-dihydroxy-2-(5-hydroxy-3,7-dimethylocta-2,6-dien-1-yl)benzoic acid | HMDB0134979 | Benzenoids | Benzene and substituted derivatives | Benzoic acids and derivatives |
| PAC-1 | 64671 | Unclassified | Unclassified | Unclassified |
| 5a,6a-Epoxy-7E-megastigmene-3a,9e-diol 3-glucoside | HMDB0031676 | Organic oxygen compounds | Organooxygen compounds | Carbohydrates and carbohydrate conjugates |
| Allopurinol | HMDB0014581 | Organoheterocyclic compounds | Pyrazolopyrimidines | Pyrazolo[3,4-d]pyrimidines |
| 2-Methylhippuric acid | HMDB0011723 | Benzenoids | Benzene and substituted derivatives | Benzoic acids and derivatives |
| Erythrono-1,4-lactone | HMDB0000349 | Organoheterocyclic compounds | Lactones | Gamma butyrolactones |
| Gluconic acid | HMDB0000625 | Organic oxygen compounds | Organooxygen compounds | Carbohydrates and carbohydrate conjugates |
| Farnesyl acetone | 265036 | Unclassified | Unclassified | Unclassified |
| 9-oxo-13-hydroxy-11-octadecenoic acid | LMFA02000248 | Lipids and lipid-like molecules | Fatty Acyls | Octadecanoids |
| D-Maltose | HMDB0000163 | Organic oxygen compounds | Organooxygen compounds | Carbohydrates and carbohydrate conjugates |
| 2-C-Methyl-D-erythritol 4-phosphate | 64013 | Unclassified | Unclassified | Unclassified |
| Ligustilide | HMDB0034277 | Organoheterocyclic compounds | Isobenzofurans | Unclassified |
| gamma-Glutamylleucine | HMDB0011171 | Organic acids and derivatives | Carboxylic acids and derivatives | Amino acids, peptides, and analogues |
| (R)-(+)-2-Pyrrolidone-5-carboxylic acid | 63632 | Unclassified | Unclassified | Unclassified |
| Phthalic acid | HMDB0002107 | Benzenoids | Benzene and substituted derivatives | Benzoic acids and derivatives |
| beta-Lactose | HMDB0041627 | Organic oxygen compounds | Organooxygen compounds | Carbohydrates and carbohydrate conjugates |
| PA(0:0/18:2(9Z,12Z)) | LMGP10050044 | Lipids and lipid-like molecules | Glycerophospholipids | Glycerophosphates |
| 9,12,15-Octadecatrien-1-ol | LMFA05000216 | Lipids and lipid-like molecules | Fatty Acyls | Fatty alcohols |
| Galactaric acid | LMFA01170107 | Organic oxygen compounds | Organooxygen compounds | Carbohydrates and carbohydrate conjugates |
| R-cucujolide V | LMFA07040048 | Lipids and lipid-like molecules | Fatty Acyls | Fatty esters |
| 10-HODTA methyl ester | 65404 | Unclassified | Unclassified | Unclassified |
| Camptothecin | 3491 | Unclassified | Unclassified | Unclassified |
| PHODA-PI | LMGP20050001 | Lipids and lipid-like molecules | Glycerophospholipids | Oxidized glycerophospholipids |
| 6-[(4,7-dihydroxy-2,2-dimethyl-3,4-dihydro-2H-1-benzopyran-5-yl)oxy]-3,4,5-trihydroxyoxane-2-carboxylic acid | HMDB0125821 | Organic oxygen compounds | Organooxygen compounds | Carbohydrates and carbohydrate conjugates |
| 6-{[5-(3,4-dihydroxyphenyl)pentanoyl]oxy}-3,4,5-trihydroxyoxane-2-carboxylic acid | HMDB0127759 | Organic oxygen compounds | Organooxygen compounds | Carbohydrates and carbohydrate conjugates |
| Cytochalasin Npho | HMDB0035367 | Alkaloids and derivatives | Cytochalasans | Unclassified |
| 1-(3,4-Dihydroxyphenyl)-1-decene-3,5-dione | 64193 | Unclassified | Unclassified | Unclassified |
| 2-Methyl-1-methylthio-2-butene | HMDB0032411 | Organosulfur compounds | Thioethers | Dialkylthioethers |
| Alpha-D-Glucopyranoside | HMDB0061922 | Organic oxygen compounds | Organooxygen compounds | Carbohydrates and carbohydrate conjugates |
| 11-Hydroxy-9-tridecenoic acid | HMDB0035881 | Lipids and lipid-like molecules | Fatty Acyls | Fatty acids and conjugates |
| 5'-Carboxy-gamma-chromanol | HMDB0012799 | Organoheterocyclic compounds | Benzopyrans | 1-benzopyrans |
| PA(18:1(11Z)/15:0) | HMDB0114898 | Lipids and lipid-like molecules | Glycerophospholipids | Glycerophosphates |
| Asymmetric dimethylarginine | HMDB0001539 | Organic acids and derivatives | Carboxylic acids and derivatives | Amino acids, peptides, and analogues |
| Threonic acid | HMDB0000943 | Organic oxygen compounds | Organooxygen compounds | Carbohydrates and carbohydrate conjugates |
| 2-(4-Hydroxyphenyl)naphthalic anhydride | HMDB0041121 | Benzenoids | Naphthalenes | Phenylnaphthalenes |
| PC(19:1(9Z)/0:0) | LMGP01050130 | Lipids and lipid-like molecules | Glycerophospholipids | Glycerophosphocholines |
| 10-Acetoxyligustroside | HMDB0035217 | Lipids and lipid-like molecules | Prenol lipids | Terpene glycosides |
| Cucurbic acid | HMDB0029388 | Lipids and lipid-like molecules | Fatty Acyls | Lineolic acids and derivatives |
| 2-hydroxyhexadecanoic acid | 34495 | Lipids and lipid-like molecules | Fatty Acyls | Fatty acids and conjugates |
| (2-Chlorophenyl)diphenylmethane | 1898 | Unclassified | Unclassified | Unclassified |
| 5alpha,6beta-dihydroxy-24-methylenecholestan-3-one | LMST01031062 | Unclassified | Unclassified | Unclassified |
| Cortisol | HMDB0000063 | Lipids and lipid-like molecules | Steroids and steroid derivatives | Hydroxysteroids |
| 2-(4-Methyl-5-thiazolyl)ethyl butanoate | HMDB0032418 | Organoheterocyclic compounds | Azoles | Thiazoles |
| Nonadecandioic acid | LMFA01170135 | Lipids and lipid-like molecules | Fatty Acyls | Fatty Acids and Conjugates |
| 17-hydroxy stearic acid | LMFA02000135 | Lipids and lipid-like molecules | Fatty Acyls | Octadecanoids |
| 2-Methoxy-3-(4-methoxyphenyl)propanoic acid | HMDB0039428 | Phenylpropanoids and polyketides | Phenylpropanoic acids | Unclassified |
| 7-Methylinosine | HMDB0003950 | Nucleosides, nucleotides, and analogues | Purine nucleosides | Unclassified |
| L-Valine | HMDB0000883 | Organic acids and derivatives | Carboxylic acids and derivatives | Amino acids, peptides, and analogues |
| PA(18:1(9Z)/0:0) | LMGP10050008 | Lipids and lipid-like molecules | Glycerophospholipids | Glycerophosphates |
| Leucyl-Aspartate | 85829 | Organic acids and derivatives | Carboxylic acids and derivatives | Amino acids, peptides, and analogues |
| Dambonitol | HMDB0033942 | Organic oxygen compounds | Organooxygen compounds | Alcohols and polyols |
| 12,13-dihydroxy-11-methoxy-9-octadecenoic acid | LMFA01080006 | Lipids and lipid-like molecules | Fatty Acyls | Fatty Acids and Conjugates |
| (1R,2R)-3-[(1,2-Dihydro-2-hydroxy-1-naphthalenyl)thio]-2-oxopropanoic acid | 70331 | Unclassified | Unclassified | Unclassified |
| PA(16:0/0:0) | 40944 | Lipids and lipid-like molecules | Glycerophospholipids | Glycerophosphates |
| 9R-hydroxy-10E,12E-octadecadienoic acid, methyl ester | LMFA01050549 | Lipids and lipid-like molecules | Fatty Acyls | Fatty Acids and Conjugates |
| Avermectin B1b monosaccharide | 63689 | Unclassified | Unclassified | Unclassified |
| Polidocanol | 69582 | Unclassified | Unclassified | Unclassified |
| Uracil | HMDB0000300 | Organoheterocyclic compounds | Diazines | Pyrimidines and pyrimidine derivatives |
| (-)-Wikstromol | HMDB0030598 | Lignans, neolignans and related compounds | Furanoid lignans | Tetrahydrofuran lignans |
| 2-Dodecylbenzenesulfonic acid | HMDB0031031 | Benzenoids | Benzene and substituted derivatives | Benzenesulfonic acids and derivatives |
| Behenic acid | HMDB0000944 | Lipids and lipid-like molecules | Fatty Acyls | Fatty acids and conjugates |
| Phenylalanylproline | HMDB0011177 | Organic acids and derivatives | Carboxylic acids and derivatives | Amino acids, peptides, and analogues |
| PON-PA | LMGP20070013 | Lipids and lipid-like molecules | Glycerophospholipids | Oxidized glycerophospholipids |
| 1-Oleoyl Lysophosphatidic Acid (sodium salt) | 5432 | Unclassified | Unclassified | Unclassified |
| L-Isoleucine | HMDB0000172 | Organic acids and derivatives | Carboxylic acids and derivatives | Amino acids, peptides, and analogues |
| D-Xylono-1,5-lactone | HMDB0011676 | Organoheterocyclic compounds | Lactones | Delta valerolactones |
| Panaxydol | LMFA05000028 | Lipids and lipid-like molecules | Fatty Acyls | Fatty alcohols |
| (±)9-HpODE | 64785 | Unclassified | Unclassified | Unclassified |
| (9R,13R)-10-oxo-11-phytoenoic acid | LMFA02010002 | Lipids and lipid-like molecules | Fatty Acyls | Octadecanoids |
| Descladinose roxithromycin | HMDB0060814 | Organic oxygen compounds | Organooxygen compounds | Carbohydrates and carbohydrate conjugates |
| LysoPC(0:0/16:0) | HMDB0240262 | Lipids and lipid-like molecules | Glycerophospholipids | Glycerophosphocholines |
| 9-hydroxy-7E-Nonene-3,5-diynoic acid | LMFA01030721 | Lipids and lipid-like molecules | Fatty Acyls | Fatty Acids and Conjugates |
| Biflorin | HMDB0002336 | Organic oxygen compounds | Organooxygen compounds | Carbohydrates and carbohydrate conjugates |
| (S)-Nerolidol 3-O-[a-L-rhamnopyranosyl-(1->4)-a-L-rhamnopyranosyl-(1->6)-b-D-glucopyranoside] | HMDB0040846 | Organic oxygen compounds | Organooxygen compounds | Carbohydrates and carbohydrate conjugates |
| 4-Dodecylbenzenesulfonic Acid | HMDB0059915 | Benzenoids | Benzene and substituted derivatives | Benzenesulfonic acids and derivatives |
| LysoPA(0:0/16:0) | HMDB0007849 | Lipids and lipid-like molecules | Glycerophospholipids | Glycerophosphates |
| Oleoyl Ethanolamide | 24103 | Organic nitrogen compounds | Organonitrogen compounds | Amines |
| Gamma-glutamyl-Isoleucine | 86044 | Unclassified | Unclassified | Unclassified |
| lysophosphatidic acid | LMGP10050045 | Lipids and lipid-like molecules | Glycerophospholipids | Glycerophosphates |
| LysoPE(0:0/16:1(9Z)) | HMDB0011474 | Lipids and lipid-like molecules | Glycerophospholipids | Glycerophosphoethanolamines |
| 1-O-(2-methoxy-hexadecyl)-sn-glycerol | LMGL01020037 | Lipids and lipid-like molecules | Glycerolipids | Monoradylglycerols |
| Galabiose | 86577 | Organic oxygen compounds | Organooxygen compounds | Carbohydrates and carbohydrate conjugates |
| PA(18:3(6Z,9Z,12Z)/0:0) | LMGP10050023 | Lipids and lipid-like molecules | Glycerophospholipids | Glycerophosphates |
| (±)9-HODE | 35487 | Unclassified | Unclassified | Unclassified |
| Selina-6-en-4-ol | 265102 | Unclassified | Unclassified | Unclassified |
| Araliacerebroside | HMDB0033621 | Lipids and lipid-like molecules | Sphingolipids | Glycosphingolipids |
| 2-Oxo-6-methylthiohexanoic acid | 64495 | Unclassified | Unclassified | Unclassified |
| Ticarcillin | HMDB0015545 | Organic acids and derivatives | Carboxylic acids and derivatives | Amino acids, peptides, and analogues |
| Glucose 6-phosphate | HMDB0001401 | Organic oxygen compounds | Organooxygen compounds | Carbohydrates and carbohydrate conjugates |
| 5-Hydroxyflavone | HMDB0040556 | Phenylpropanoids and polyketides | Flavonoids | Flavonoid glycosides |
| 1-Nitro-5,6-dihydroxy-dihydronaphthalene | HMDB0060328 | Benzenoids | Naphthalenes | Nitronaphthalenes |
| PE(17:1(9Z)/0:0) | 46714 | Lipids and lipid-like molecules | Glycerophospholipids | Glycerophosphoethanolamines |
| Ketophenylbutazone | HMDB0041914 | Benzenoids | Benzene and substituted derivatives | Unclassified |
| ent-9-L1-PhytoP | LMFA02030003 | Lipids and lipid-like molecules | Fatty Acyls | Octadecanoids |
| 3'-Glucosyl-2',4',6'-trihydroxyacetophenone | HMDB0040621 | Organic oxygen compounds | Organooxygen compounds | Carbohydrates and carbohydrate conjugates |
| (3S,5R,6S,7E,9x)-7-Megastigmene-3,6,9-triol 9-glucoside | LMFA13010057 | Lipids and lipid-like molecules | Fatty Acyls | Fatty acyl glycosides |
| Aspoxicillin | 69555 | Unclassified | Unclassified | Unclassified |
| 4-Methoxyglucobrassicin | 66963 | Unclassified | Unclassified | Unclassified |
| Citrusin A | HMDB0039230 | Lignans, neolignans and related compounds | Lignan glycosides | Unclassified |
| 8-Hydroxypinoresinol 8-glucoside | HMDB0033281 | Lignans, neolignans and related compounds | Lignan glycosides | Unclassified |
| PE-Cer(d14:2(4E,6E)/24:1(15Z)) | LMSP03020037 | Lipids and lipid-like molecules | Sphingolipids | Phosphosphingolipids |
| Melibiose | HMDB0000048 | Organic oxygen compounds | Organooxygen compounds | Carbohydrates and carbohydrate conjugates |
| gamma-Carboxyglutamic acid | HMDB0041900 | Organic acids and derivatives | Carboxylic acids and derivatives | Amino acids, peptides, and analogues |
| Adenosine | HMDB0000050 | Nucleosides, nucleotides, and analogues | Purine nucleosides | Unclassified |
| D-glycero-L-galacto-Octulose | HMDB0029954 | Organic oxygen compounds | Organooxygen compounds | Carbohydrates and carbohydrate conjugates |
| Metaproterenol 3-O-sulfate | 1178 | Unclassified | Unclassified | Unclassified |
| N-Acetylhistidine | HMDB0032055 | Organic acids and derivatives | Carboxylic acids and derivatives | Amino acids, peptides, and analogues |
| 9(10)-EpODE | HMDB0010220 | Lipids and lipid-like molecules | Fatty Acyls | Fatty acids and conjugates |
| Pyrroline hydroxycarboxylic acid | HMDB0001369 | Organic acids and derivatives | Carboxylic acids and derivatives | Amino acids, peptides, and analogues |
| Maltotriose | HMDB0001262 | Organic oxygen compounds | Organooxygen compounds | Carbohydrates and carbohydrate conjugates |
| PA(0:0/18:1(9Z)) | LMGP10050014 | Lipids and lipid-like molecules | Glycerophospholipids | Glycerophosphates |
| 2-(2-Phenylacetoxy)propionylglycine | HMDB0059732 | Organic acids and derivatives | Carboxylic acids and derivatives | Amino acids, peptides, and analogues |
| Harmalan | HMDB0029834 | Alkaloids and derivatives | Harmala alkaloids | Unclassified |
| DG(8:0/8:0/0:0) | HMDB0116368 | Lipids and lipid-like molecules | Glycerolipids | Diradylglycerols |
| Tricosanoylglycine | HMDB0013314 | Organic acids and derivatives | Carboxylic acids and derivatives | Amino acids, peptides, and analogues |
| L-Phenylalanine | HMDB0000159 | Organic acids and derivatives | Carboxylic acids and derivatives | Amino acids, peptides, and analogues |
| Pyroglutamylvaline | HMDB0094651 | Organic acids and derivatives | Carboxylic acids and derivatives | Amino acids, peptides, and analogues |
| NVP-BEZ235 | 64764 | Unclassified | Unclassified | Unclassified |
| Gluconasturtiin | HMDB0038423 | Organic oxygen compounds | Organooxygen compounds | Carbohydrates and carbohydrate conjugates |
| Ethyl beta-D-glucopyranoside | HMDB0029968 | Organic oxygen compounds | Organooxygen compounds | Carbohydrates and carbohydrate conjugates |
| OON-PA | LMGP20070023 | Lipids and lipid-like molecules | Glycerophospholipids | Oxidized glycerophospholipids |
| N-Deschlorobenzoyl indomethacin | HMDB0013988 | Organoheterocyclic compounds | Indoles and derivatives | Indolyl carboxylic acids and derivatives |
| 6-Keto-decanoylcarnitine | LMFA07070047 | Lipids and lipid-like molecules | Fatty Acyls | Fatty acid esters |
| {3,8,15-trihydroxy-16,17-dimethoxy-9-oxotricyclo[12.3.1.1²,⁶]nonadeca-1(17),2,4,6(19),14(18),15-hexaen-7-yl}oxidanesulfonic acid | HMDB0133716 | Phenylpropanoids and polyketides | Diarylheptanoids | Cyclic diarylheptanoids |
| 3-Deoxyarabinohexonic acid | HMDB0000346 | Organic acids and derivatives | Hydroxy acids and derivatives | Medium-chain hydroxy acids and derivatives |
| (R)-Mevalonic acid-5-pyrophosphate | HMDB0001981 | Organic oxygen compounds | Organic oxoanionic compounds | Organic pyrophosphates |
| Methyl (3x,10R)-dihydroxy-11-dodecene-6,8-diynoate 10-glucoside | HMDB0040896 | Lipids and lipid-like molecules | Fatty Acyls | Fatty acyl glycosides |
| Xestoaminol C | 53933 | Lipids and lipid-like molecules | Sphingolipids | Sphingoid bases |
| 5-Hydroxymethyl-2-furancarboxaldehyde | HMDB0034355 | Organic oxygen compounds | Organooxygen compounds | Carbonyl compounds |
| N-Undecylbenzenesulfonic acid | HMDB0032549 | Benzenoids | Benzene and substituted derivatives | Benzenesulfonic acids and derivatives |
| 7-Methylxanthine | HMDB0001991 | Organoheterocyclic compounds | Imidazopyrimidines | Purines and purine derivatives |
| (R)C(R)S-S-Propylcysteine sulfoxide | HMDB0029442 | Organic acids and derivatives | Carboxylic acids and derivatives | Amino acids, peptides, and analogues |
| MG(18:0/0:0/0:0) | 61993 | Lipids and lipid-like molecules | Glycerolipids | Monoradylglycerols |
| Di-n-octyl phthalate | 69894 | Unclassified | Unclassified | Unclassified |
| 6-[1]-ladderane hexanol | LMFA01140088 | Lipids and lipid-like molecules | Fatty Acyls | Fatty Acids and Conjugates |
| SB 221284 | 69190 | Unclassified | Unclassified | Unclassified |
| 4-Butyl-5-propylthiazole | 94608 | Organoheterocyclic compounds | Azoles | Thiazoles |
| PE(14:0/24:0) | HMDB0008848 | Lipids and lipid-like molecules | Glycerophospholipids | Glycerophosphoethanolamines |
| N-Phenylacetylglutamic acid | HMDB0059772 | Organic acids and derivatives | Carboxylic acids and derivatives | Amino acids, peptides, and analogues |
| 14-keto pentadecanoic acid | LMFA01060049 | Lipids and lipid-like molecules | Fatty Acyls | Fatty Acids and Conjugates |
| Cyproheptadine | 1919 | Benzenoids | Dibenzocycloheptenes | Unclassified |
| Diethylpropion(metabolite VIII-glucuronide) | 2023 | Unclassified | Unclassified | Unclassified |
| 16-B1-PhytoP | LMFA02030004 | Lipids and lipid-like molecules | Fatty Acyls | Octadecanoids |
| 27-Norcholestanehexol | LMST04020029 | Lipids and lipid-like molecules | Steroids and steroid derivatives | Bile acids, alcohols and derivatives |
| LysoPC(18:1(9Z)) | HMDB0002815 | Lipids and lipid-like molecules | Glycerophospholipids | Glycerophosphocholines |
| Diethylhexyl adipate | HMDB0040270 | Lipids and lipid-like molecules | Fatty Acyls | Fatty acid esters |
| L-Fucose | HMDB0000174 | Organic oxygen compounds | Organooxygen compounds | Carbohydrates and carbohydrate conjugates |
| OHDdiA-PG | LMGP20060036 | Lipids and lipid-like molecules | Glycerophospholipids | Oxidized glycerophospholipids |
| 2-methyl-dodecanedioic acid | LMFA01170010 | Lipids and lipid-like molecules | Fatty Acyls | Fatty Acids and Conjugates |
| 12Z,15Z-heneicosadienoic acid | LMFA01030399 | Lipids and lipid-like molecules | Fatty Acyls | Fatty Acids and Conjugates |
| 12-Dehydroporson | 95815 | Lipids and lipid-like molecules | Fatty Acyls | Fatty acyl glycosides |
| Kanzonol J | HMDB0040605 | Phenylpropanoids and polyketides | Isoflavonoids | Pyranoisoflavonoids |
| Epicatechin | HMDB0001871 | Phenylpropanoids and polyketides | Flavonoids | Flavans |
| Dihomomethionine | 64496 | Unclassified | Unclassified | Unclassified |
| Icariside B8 | HMDB0036846 | Lipids and lipid-like molecules | Prenol lipids | Terpene glycosides |
| 12S-hydroxy-16-heptadecynoic acid | LMFA01050146 | Lipids and lipid-like molecules | Fatty Acyls | Fatty Acids and Conjugates |
| 2,4,6-Trithiaheptane | 88519 | Organosulfur compounds | Thioacetals | Dithioacetals |
| (3beta,5alpha,6beta,22E,24R)-23-Methylergosta-7,22-diene-3,5,6-triol | 89433 | Lipids and lipid-like molecules | Steroids and steroid derivatives | Ergostane steroids |
| (3alphaOH,20S,24S)-3,19:20,24-Diepoxydammarane-3,25-diol | HMDB0034683 | Lipids and lipid-like molecules | Prenol lipids | Terpene glycosides |
| L-Tryptophan | HMDB0000929 | Organoheterocyclic compounds | Indoles and derivatives | Indolyl carboxylic acids and derivatives |
| Crosatoside B | HMDB0039253 | Organic oxygen compounds | Organooxygen compounds | Carbohydrates and carbohydrate conjugates |
| 13-Hydroxy-9-methoxy-10-oxo-11-octadecenoic acid | HMDB0040901 | Lipids and lipid-like molecules | Fatty Acyls | Lineolic acids and derivatives |
| Loxoprofen Metabolite trans-glucuronide | 1043 | Unclassified | Unclassified | Unclassified |
| 10-amino-decanoic acid | LMFA01100002 | Lipids and lipid-like molecules | Fatty Acyls | Fatty Acids and Conjugates |
| 3',4'-Methylenedioxyfurano[2'',3'':6,7]aurone | LMPK12130007 | Unclassified | Unclassified | Unclassified |
| methyl 15-hydroperoxy-9Z,12Z,16E-octadecatrienoate | 74464 | Lipids and lipid-like molecules | Fatty Acyls | Fatty Acids and Conjugates |
| Calpeptin | 68942 | Unclassified | Unclassified | Unclassified |
| Lupiwighteone hydrate 7-glucoside | HMDB0034813 | Phenylpropanoids and polyketides | Isoflavonoids | Isoflavonoid O-glycosides |
| trans-3-Hydroxycotinine glucuronide | 6080 | Organic oxygen compounds | Organooxygen compounds | Carbohydrates and carbohydrate conjugates |
| Alendronic acid | 818 | Organic acids and derivatives | Organic phosphonic acids and derivatives | Bisphosphonates |
| [6]-Gingerdiol 3-acetate | 95051 | Lipids and lipid-like molecules | Fatty Acyls | Fatty alcohol esters |
| Brosimacutin G | LMPK12120035 | Lipids and lipid-like molecules | Polyketides | Flavonoids |
| Alhagidin | 52865 | Lipids and lipid-like molecules | Polyketides | Flavonoids |
| L-Oxalylalbizziine | HMDB0039164 | Organic acids and derivatives | Carboxylic acids and derivatives | Amino acids, peptides, and analogues |
| 1-(9Z,12Z,15Z-octadecatrienoyl)-glycero-3-phosphate | HMDB0062320 | Lipids and lipid-like molecules | Glycerophospholipids | Glycerophosphates |
| PE(16:1(9Z)/0:0) | LMGP02050010 | Lipids and lipid-like molecules | Glycerophospholipids | Glycerophosphoethanolamines |
| 1-(5-Phosphoribosyl)-4-(N-succinocarboxamide)-5-aminoimidazole | 66260 | Unclassified | Unclassified | Unclassified |
| Phenethyl decanoate | HMDB0032464 | Lipids and lipid-like molecules | Fatty Acyls | Fatty acid esters |
| PE(P-16:0/0:0) | 46719 | Lipids and lipid-like molecules | Glycerophospholipids | Glycerophosphoethanolamines |
| DG(18:2(9Z,12Z)/22:4(7Z,10Z,13Z,16Z)/0:0) | HMDB0007263 | Lipids and lipid-like molecules | Glycerolipids | Diradylglycerols |
| AVOCADYNONE ACETATE | 43724 | Unclassified | Unclassified | Unclassified |
| Butyl salicylate | HMDB0040730 | Benzenoids | Benzene and substituted derivatives | Benzoic acids and derivatives |
| 6-Hydroxy-2-bornanone glucoside | HMDB0034558 | Lipids and lipid-like molecules | Prenol lipids | Terpene glycosides |
| (±)14(15)-EET methyl ester | 62934 | Unclassified | Unclassified | Unclassified |
| 3-O-(6-O-alpha-D-Xylosylphospho-alpha-D-mannopyranosyl)-alpha-D-mannopyranose | 73349 | Unclassified | Unclassified | Unclassified |
| D-Galactose | HMDB0000143 | Organic oxygen compounds | Organooxygen compounds | Carbohydrates and carbohydrate conjugates |
| Pipecuronium | HMDB0015431 | Lipids and lipid-like molecules | Steroids and steroid derivatives | Steroid esters |
| 3,4,5-trihydroxy-6-({7-oxo-7H-furo[3,2-g]chromen-4-yl}oxy)oxane-2-carboxylic acid | HMDB0129424 | Organic oxygen compounds | Organooxygen compounds | Carbohydrates and carbohydrate conjugates |
| (R)-(Homo)3-citrate | 71255 | Unclassified | Unclassified | Unclassified |
| {[6-(5,7-dihydroxy-4-oxo-3,4-dihydro-2H-1-benzopyran-2-yl)-7-hydroxy-2-(4-methylpent-3-en-1-yl)-2H-chromen-2-yl]methoxy}sulfonic acid | HMDB0132814 | Phenylpropanoids and polyketides | Flavonoids | Pyranoflavonoids |
| 15-hydroxy stearic acid | LMFA02000133 | Lipids and lipid-like molecules | Fatty Acyls | Octadecanoids |
| PS(P-20:0/0:0) | LMGP03070001 | Lipids and lipid-like molecules | Glycerophospholipids | Glycerophosphoserines |
| Serylisoleucine | HMDB0029042 | Organic acids and derivatives | Carboxylic acids and derivatives | Amino acids, peptides, and analogues |
| Endothion | 72741 | Unclassified | Unclassified | Unclassified |
| Sterebin E | HMDB0035379 | Lipids and lipid-like molecules | Prenol lipids | Diterpenoids |
| PA(0:0/18:0) | LMGP10050043 | Lipids and lipid-like molecules | Glycerophospholipids | Glycerophosphates |
| 5alpha-Cholestane | HMDB0041632 | Lipids and lipid-like molecules | Steroids and steroid derivatives | Cholestane steroids |
| (x)-2-Heptanol glucoside | HMDB0035028 | Lipids and lipid-like molecules | Fatty Acyls | Fatty acyl glycosides |
| 12-Hydroxy-7-oxo-8,11,13-abietatrien-18-al | HMDB0040746 | Lipids and lipid-like molecules | Prenol lipids | Diterpenoids |
| Arginyl-Histidine | HMDB0028711 | Organic acids and derivatives | Carboxylic acids and derivatives | Amino acids, peptides, and analogues |
| Riesling acetal | HMDB0037562 | Organoheterocyclic compounds | Tetrahydrofurans | Unclassified |
| UNC0224 | 64826 | Unclassified | Unclassified | Unclassified |
| [1-(4-methoxyphenyl)-3-phenylpropoxy]sulfonic acid | HMDB0135641 | Phenylpropanoids and polyketides | Linear 1,3-diarylpropanoids | Unclassified |
| Methyl n-acetylanthranilate | HMDB0032388 | Benzenoids | Benzene and substituted derivatives | Benzoic acids and derivatives |
| Isopropyl tetradecanoate | LMFA07010677 | Lipids and lipid-like molecules | Fatty Acyls | Fatty acid esters |
| 2-Phenylaminoadenosine | HMDB0001069 | Nucleosides, nucleotides, and analogues | Purine nucleosides | Unclassified |
| Spironolactone | HMDB0014565 | Lipids and lipid-like molecules | Steroids and steroid derivatives | Steroid lactones |
| MAB3 | 48375 | Phenylpropanoids and polyketides | Neoflavonoids | Prenylated neoflavonoids |
| LysoPC(16:1(9Z)/0:0) | HMDB0010383 | Lipids and lipid-like molecules | Glycerophospholipids | Glycerophosphocholines |
| N-(1-Deoxy-1-fructosyl)tyrosine | HMDB0037845 | Organic acids and derivatives | Carboxylic acids and derivatives | Amino acids, peptides, and analogues |
| 11-methoxy-12,13-epoxy-9-octadecenoic acid | LMFA01080007 | Lipids and lipid-like molecules | Fatty Acyls | Fatty Acids and Conjugates |
| Lyso-PAF C-16 | 24070 | Unclassified | Unclassified | Unclassified |
| 6-[(4,5-dihydroxy-2,2-dimethyl-3,4-dihydro-2H-1-benzopyran-7-yl)oxy]-3,4,5-trihydroxyoxane-2-carboxylic acid | HMDB0125822 | Organic oxygen compounds | Organooxygen compounds | Carbohydrates and carbohydrate conjugates |
| Linoleic acid | HMDB0000673 | Lipids and lipid-like molecules | Fatty Acyls | Lineolic acids and derivatives |
| Tyrosyl-Leucine | HMDB0029109 | Organic acids and derivatives | Carboxylic acids and derivatives | Amino acids, peptides, and analogues |
| Azaserine | 72922 | Unclassified | Unclassified | Unclassified |
| Dimethyl 3-methoxy-4-oxo-5-(8,11,14-pentadecatrienyl)-2-hexenedioate | HMDB0032099 | Organic acids and derivatives | Keto acids and derivatives | Beta-keto acids and derivatives |
| 3-O-Mycarosylerythronolide B | 63709 | Unclassified | Unclassified | Unclassified |
| Cerulenin | HMDB0015168 | Organoheterocyclic compounds | Epoxides | Oxirane carboxylic acids and derivatives |
| (E)-4-(6-methyl-1,2-dithiin-3-yl)but-1-en-3-yn-1-ol | LMFA12000367 | Lipids and lipid-like molecules | Fatty Acyls | Oxygenated hydrocarbons |
| (±)-3-(4-Hydroxyphenyl)-1,2-propanediol 4'-O-glucoside | 88974 | Organic oxygen compounds | Organooxygen compounds | Carbohydrates and carbohydrate conjugates |
| 5-Androstene-3b,16b,17a-triol | 5508 | Lipids and lipid-like molecules | Steroids and steroid derivatives | Androstane steroids |
| MEFEXAMIDE | 43468 | Unclassified | Unclassified | Unclassified |
| {6-hydroxy-3-[3-(3-hydroxyphenyl)-3-oxopropyl]-2-methoxyphenyl}oxidanesulfonic acid | HMDB0126077 | Phenylpropanoids and polyketides | Linear 1,3-diarylpropanoids | Chalcones and dihydrochalcones |
| Pectachol | HMDB0039064 | Phenylpropanoids and polyketides | Coumarins and derivatives | Unclassified |
| Methyl-(10R)-hydroxy-(11S,12S)-epoxy-(5Z,8Z,14Z)-eicosatrienoate | LMFA03090014 | Lipids and lipid-like molecules | Fatty Acyls | Eicosanoids |
| Prosulfocarb | 72532 | Unclassified | Unclassified | Unclassified |
| CAY10512 | 45185 | Unclassified | Unclassified | Unclassified |
| PA(P-16:0/0:0) | LMGP10070003 | Lipids and lipid-like molecules | Glycerophospholipids | Glycerophosphates |
| TOFA | 34518 | Unclassified | Unclassified | Unclassified |
| PC(O-12:0/0:0)[U] | 40380 | Unclassified | Unclassified | Unclassified |
| Yucalexin P15 | HMDB0036753 | Lipids and lipid-like molecules | Prenol lipids | Diterpenoids |
| 9-hydroxy-16-oxo-hexadecanoic acid | LMFA01170059 | Lipids and lipid-like molecules | Fatty Acyls | Fatty Acids and Conjugates |
| 13S-HpODE | LMFA02000034 | Lipids and lipid-like molecules | Fatty Acyls | Octadecanoids |
| 5,9,17-hexacosatrienoic acid | LMFA01030426 | Lipids and lipid-like molecules | Fatty Acyls | Fatty Acids and Conjugates |
| PE(19:1(9Z)/0:0) | 77685 | Lipids and lipid-like molecules | Glycerophospholipids | Glycerophosphoethanolamines |
| Nandrolone | HMDB0002725 | Lipids and lipid-like molecules | Steroids and steroid derivatives | Estrane steroids |
| Licoagrodin | HMDB0035372 | Phenylpropanoids and polyketides | Flavonoids | Flavans |
| IC202A | LMFA08020182 | Lipids and lipid-like molecules | Fatty Acyls | Fatty amides |
| L-cis-Cyclo(aspartylphenylalanyl) | HMDB0031360 | Organic acids and derivatives | Carboxylic acids and derivatives | Amino acids, peptides, and analogues |
| 11-oxo-undeca-5,8-dienoic acid | LMFA01060220 | Lipids and lipid-like molecules | Fatty Acyls | Fatty Acids and Conjugates |
| PA(20:1(11Z)/0:0) | LMGP10050026 | Lipids and lipid-like molecules | Glycerophospholipids | Glycerophosphates |
| MG(15:0/0:0/0:0) | 62346 | Lipids and lipid-like molecules | Glycerolipids | Monoradylglycerols |
| Hex-Honaucin A | 65488 | Unclassified | Unclassified | Unclassified |
| Ketotifen-N-glucuronide | HMDB0060596 | Organic oxygen compounds | Organooxygen compounds | Carbohydrates and carbohydrate conjugates |
| Butyl (S)-3-hydroxybutyrate [arabinosyl-(1->6)-glucoside] | HMDB0039214 | Lipids and lipid-like molecules | Fatty Acyls | Fatty acyl glycosides |
| [10]-Shogaol | 87698 | Benzenoids | Phenols | Methoxyphenols |
| 11-Oxo-androsterone glucuronide | LMST05010032 | Lipids and lipid-like molecules | Glycerolipids | Diradylglycerols |
| Stearyl monoglyceridyl citrate | 88504 | Unclassified | Unclassified | Unclassified |
| 3,6-octadecadiynoic acid | LMFA01030515 | Lipids and lipid-like molecules | Fatty Acyls | Fatty Acids and Conjugates |
| (+/-)N-(1-methyl-2-hydroxy-2-phenyl-ethyl) arachidonyl amine | LMFA08020030 | Lipids and lipid-like molecules | Fatty Acyls | Fatty amides |
| O-Ureidohomoserine | HMDB0012271 | Organic acids and derivatives | Carboxylic acids and derivatives | Amino acids, peptides, and analogues |
| PtdIns-(1,2-dihexanoyl) | 64898 | Unclassified | Unclassified | Unclassified |
| Asparaginyl-Isoleucine | HMDB0028734 | Organic acids and derivatives | Carboxylic acids and derivatives | Amino acids, peptides, and analogues |
| Binapacryl | 72773 | Unclassified | Unclassified | Unclassified |
| Cyclobrassinin | HMDB0033352 | Organoheterocyclic compounds | Indoles and derivatives | Indoles |
| Melledonal A | HMDB0035865 | Lipids and lipid-like molecules | Prenol lipids | Sesquiterpenoids |
| Promazine N-oxide sulfoxide | 2134 | Unclassified | Unclassified | Unclassified |
| 11-trans-LTE4 | LMFA03020022 | Lipids and lipid-like molecules | Fatty Acyls | Eicosanoids |
| Aucubin | LMPR0102070006 | Lipids and lipid-like molecules | Prenol lipids | Terpene glycosides |
| 3',4'-Methylenedioxy-[2'',3'':7,8]furanoflavanone | LMPK12140068 | Unclassified | Unclassified | Unclassified |
| 6-Oxopiperidine-2-carboxylic acid | HMDB0061705 | Organic acids and derivatives | Carboxylic acids and derivatives | Amino acids, peptides, and analogues |
| Pergolide | HMDB0015317 | Organoheterocyclic compounds | Quinolines and derivatives | Indoloquinolines |
| PS(22:4(7Z,10Z,13Z,16Z)/22:5(4Z,7Z,10Z,13Z,16Z)) | HMDB0112811 | Lipids and lipid-like molecules | Glycerophospholipids | Glycerophosphoserines |
| (±)-(Z)-2-(5-Tetradecenyl)cyclobutanone | HMDB0037543 | Organic oxygen compounds | Organooxygen compounds | Carbonyl compounds |
| (1R,2R)-3-oxo-2-pentyl-cyclopentanebutanoic acid | LMFA02010017 | Lipids and lipid-like molecules | Fatty Acyls | Octadecanoids |
| N-Acetyldehydroanonaine | HMDB0041537 | Alkaloids and derivatives | Aporphines | Unclassified |
| 6beta-acetoxy-24-methylcholestan-3beta,5alpha,22R,24-tetrol | 84015 | Unclassified | Unclassified | Unclassified |
| N-dodecanoyl-L-Homoserine lactone-3-hydrazone-fluorescein | 64901 | Unclassified | Unclassified | Unclassified |
| N2'-Acetylgentamicin C1a | 65995 | Unclassified | Unclassified | Unclassified |
| PE-Cer(d16:2(4E,6E)/24:1(15Z)) | LMSP03020055 | Lipids and lipid-like molecules | Sphingolipids | Phosphosphingolipids |
| (+)-Myristinin A | LMPK12020229 | Lipids and lipid-like molecules | Polyketides | Flavonoids |
| 9Z-Octadecene-12,14-diynoic acid | LMFA01030739 | Lipids and lipid-like molecules | Fatty Acyls | Fatty Acids and Conjugates |
| {4-[5,7-dihydroxy-8-(3-methylbut-2-en-1-yl)-4-oxo-3,4-dihydro-2H-1-benzopyran-3-yl]-2-hydroxy-6-(3-methylbut-2-en-1-yl)phenyl}oxidanesulfonic acid | HMDB0134748 | Phenylpropanoids and polyketides | Isoflavonoids | Isoflavans |
| Marimastat | HMDB0014924 | Organic oxygen compounds | Organooxygen compounds | Alcohols and polyols |
| DG(18:4(6Z,9Z,12Z,15Z)/18:3(6Z,9Z,12Z)/0:0) | 58875 | Lipids and lipid-like molecules | Fatty Acyls | Lineolic acids and derivatives |
| 4-hydroxy-butyric acid | LMFA01050006 | Lipids and lipid-like molecules | Fatty Acyls | Fatty acids and conjugates |
| 19-Hydroxy-8-O-methyltetrangulol | 63758 | Unclassified | Unclassified | Unclassified |
| Molybdopterin | 6546 | Unclassified | Unclassified | Unclassified |
| Phenylalanyl-Threonine | HMDB0029005 | Organic acids and derivatives | Carboxylic acids and derivatives | Amino acids, peptides, and analogues |
| Bis(1-aziridinyl)morpholinophosphine sulfide | 73052 | Unclassified | Unclassified | Unclassified |
| PC(14:2(Z)l8,8/0:0)[U] | 40281 | Unclassified | Unclassified | Unclassified |
| Pseudouridine | HMDB0000767 | Nucleosides, nucleotides, and analogues | Nucleoside and nucleotide analogues | Unclassified |
| Gymnodimine | 73510 | Organoheterocyclic compounds | Pyridines and derivatives | Hydropyridines |
| Decylubiquinol | 70946 | Unclassified | Unclassified | Unclassified |
| Nitrendipine | HMDB0015187 | Organoheterocyclic compounds | Pyridines and derivatives | Hydropyridines |
| Tranylcypromine glucuronide | 2897 | Unclassified | Unclassified | Unclassified |
| 2-Amino-3-carboxymuconic acid semialdehyde | HMDB0001330 | Organic acids and derivatives | Carboxylic acids and derivatives | Amino acids, peptides, and analogues |
| (-)-Blebbistatin | 45431 | Unclassified | Unclassified | Unclassified |
| Stachyose | HMDB0003553 | Organic oxygen compounds | Organooxygen compounds | Carbohydrates and carbohydrate conjugates |
| Coniferaldehyde | HMDB0141782 | Benzenoids | Phenols | Methoxyphenols |
| AVOCADYNE ACETATE | 43513 | Unclassified | Unclassified | Unclassified |
| Demethyloleuropein | HMDB0036121 | Phenylpropanoids and polyketides | Flavonoids | Flavonoid glycosides |
| 3-Hydroxychavicol 1-glucoside | 90251 | Organic oxygen compounds | Organooxygen compounds | Carbohydrates and carbohydrate conjugates |
| Zalcitabine diphosphate | 3064 | Unclassified | Unclassified | Unclassified |
| gamma-Glutamylglutamic acid | HMDB0011737 | Organic acids and derivatives | Carboxylic acids and derivatives | Amino acids, peptides, and analogues |
| PA(14:0/0:0)[U] | 40947 | Unclassified | Unclassified | Unclassified |
| Valyl-Lysine | HMDB0029132 | Organic acids and derivatives | Carboxylic acids and derivatives | Amino acids, peptides, and analogues |
| 9,12,15-octadecatrienal | LMFA06000102 | Lipids and lipid-like molecules | Fatty Acyls | Fatty aldehydes |
| (25S)-5alpha-cholestan-3beta,4beta,6alpha,7alpha,8beta,15beta,16beta,26-octol | LMST01010322 | Unclassified | Unclassified | Unclassified |
| phorbol 13-acetate | LMPR0104330003 | Lipids and lipid-like molecules | Prenol lipids | Isoprenoids |
| L-Threonine | HMDB0000167 | Organic acids and derivatives | Carboxylic acids and derivatives | Amino acids, peptides, and analogues |
| Compactin diol lactone | 69858 | Unclassified | Unclassified | Unclassified |
| 8-Hydroxycarteolol | HMDB0060990 | Organoheterocyclic compounds | Quinolines and derivatives | Quinolones and derivatives |
| 6-Caffeoylsucrose | HMDB0035486 | Lipids and lipid-like molecules | Steroids and steroid derivatives | Steroidal glycosides |
| 9(R)-HODE | 45660 | Unclassified | Unclassified | Unclassified |
| LysoPE(22:1(13Z)/0:0) | HMDB0011521 | Lipids and lipid-like molecules | Glycerophospholipids | Glycerophosphoethanolamines |
| TGX-221 | 45059 | Unclassified | Unclassified | Unclassified |
| Troglitazone glucuronide | 2973 | Unclassified | Unclassified | Unclassified |
| Abscisic alcohol 11-glucoside | HMDB0039636 | Lipids and lipid-like molecules | Prenol lipids | Terpene glycosides |
| Rhamnetin 3-rhamnosyl-(1->3)(4'''-acetylrhamnosyl)(1->6)-galactoside | LMPK12112605 | Lipids and lipid-like molecules | Polyketides | Flavonoids |
| (1S,2S)-3-oxo-2-pentyl-cyclopentanebutanoic acid | LMFA02010016 | Lipids and lipid-like molecules | Fatty Acyls | Octadecanoids |
| L-N-(1H-Indol-3-ylacetyl)glutamic acid | HMDB0038665 | Organic acids and derivatives | Carboxylic acids and derivatives | Amino acids, peptides, and analogues |
| Indicaxanthin | HMDB0029386 | Organic acids and derivatives | Carboxylic acids and derivatives | Amino acids, peptides, and analogues |
| gamma-Glutamylasparagine | HMDB0029144 | Organic acids and derivatives | Carboxylic acids and derivatives | Amino acids, peptides, and analogues |
| Palmitoyl Ethanolamide | 43210 | Organic acids and derivatives | Carboximidic acids and derivatives | Carboximidic acids |
| 2-Keto-3-deoxyoctonate (KDO) | 4143 | Unclassified | Unclassified | Unclassified |
| Practolol | HMDB0015411 | Benzenoids | Benzene and substituted derivatives | Anilides |
| Flumetover | 68705 | Unclassified | Unclassified | Unclassified |
| 3Z,6Z,9Z,12Z,15Z,19Z,22Z,25Z,28Z-Hentriacontanonaene | LMFA11000016 | Lipids and lipid-like molecules | Fatty Acyls | Hydrocarbons |
| Apigenin 4'-[feruloyl-(->2)-glucuronyl-(1->2)-glucuronide] | HMDB0038295 | Phenylpropanoids and polyketides | Flavonoids | Flavonoid glycosides |
| 1-O-(2R-hydroxy-4Z-tetradecenyl)-sn-glycerol | LMGL01020053 | Lipids and lipid-like molecules | Glycerolipids | Monoradylglycerols |
| Nonoxynol-9 | HMDB0015680 | Organic oxygen compounds | Organooxygen compounds | Ethers |
| 3-Hydroxysintaxanthin | 90909 | Lipids and lipid-like molecules | Prenol lipids | Triterpenoids |
| Petasalbin | HMDB0035639 | Lipids and lipid-like molecules | Prenol lipids | Sesquiterpenoids |
| DG(18:1(11Z)/18:4(6Z,9Z,12Z,15Z)/0:0) | HMDB0007193 | Lipids and lipid-like molecules | Fatty Acyls | Lineolic acids and derivatives |
| alpha-hydroxysalmeterol | HMDB0061045 | Benzenoids | Benzene and substituted derivatives | Benzyl alcohols |
| Wasalexin A | HMDB0034765 | Organoheterocyclic compounds | Indoles and derivatives | Unclassified |
| Chlorantraniliprole | 72260 | Unclassified | Unclassified | Unclassified |
| Goshonoside F6 | HMDB0038542 | Lipids and lipid-like molecules | Prenol lipids | Terpene glycosides |
| (22E)-3alpha,12alpha-Dihydroxy-5beta-chol-22-en-24-oic Acid | LMST04010230 | Unclassified | Unclassified | Unclassified |
| 24a,24b-Dihomo-9,10-secocholesta-5,7,10(19),24a-tetraen-1α,3,25-triol | 42549 | Unclassified | Unclassified | Unclassified |
| Linamarin | HMDB0033699 | Organic oxygen compounds | Organooxygen compounds | Carbohydrates and carbohydrate conjugates |
| METHOPRENE (S) | 44012 | Unclassified | Unclassified | Unclassified |
| Acetylagmatine | 65718 | Unclassified | Unclassified | Unclassified |
| N-(5,8,11,14-eicosatetraynoyl)-ethanolamine | LMFA08040014 | Lipids and lipid-like molecules | Fatty Acyls | Fatty amides |
| POV-PA | LMGP20070015 | Lipids and lipid-like molecules | Glycerophospholipids | Oxidized glycerophospholipids |
| PE-Cer(d14:1(4E)/20:0(2OH)) | LMSP03020060 | Lipids and lipid-like molecules | Sphingolipids | Phosphosphingolipids |
| 6-{3,5-dihydroxy-2-[3,4,5-trihydroxy-6-(hydroxymethyl)oxan-2-yl]phenoxy}-3,4,5-trihydroxyoxane-2-carboxylic acid | HMDB0132676 | Organic oxygen compounds | Organooxygen compounds | Carbohydrates and carbohydrate conjugates |
| PS(P-16:0/0:0) | LMGP03070003 | Lipids and lipid-like molecules | Glycerophospholipids | Glycerophosphoserines |
| N-Jasmonoylisoleucine | HMDB0029391 | Organic acids and derivatives | Carboxylic acids and derivatives | Amino acids, peptides, and analogues |
| Kabiramide C | 65484 | Unclassified | Unclassified | Unclassified |
| Glutinic acid | LMFA01170138 | Lipids and lipid-like molecules | Fatty Acyls | Fatty Acids and Conjugates |
| Gluten exorphin A5 | HMDB0059793 | Organic acids and derivatives | Carboxylic acids and derivatives | Amino acids, peptides, and analogues |
| 4'-Methoxychalcone | LMPK12120188 | Phenylpropanoids and polyketides | Linear 1,3-diarylpropanoids | Chalcones and dihydrochalcones |
| 9-hydroperoxy-10E,12,15Z-octadecatrienoic acid | 35356 | Unclassified | Unclassified | Unclassified |
| 5,6,7-trihydroxy-3-phenyl-4H-chromen-4-one | HMDB0129963 | Phenylpropanoids and polyketides | Isoflavonoids | Isoflav-2-enes |
| Citroside A | HMDB0030370 | Lipids and lipid-like molecules | Prenol lipids | Terpene glycosides |
| Captopril disulfide | 1479 | Unclassified | Unclassified | Unclassified |
| 3-O-Benzyl-4,5-O-(1-methylethyldiene)-b-D-fructopyranose | 85019 | Unclassified | Unclassified | Unclassified |
| 10-Hydroxyloganin | LMPR0102070028 | Lipids and lipid-like molecules | Prenol lipids | Isoprenoids |
| methyl 9,10-epoxy-12,15-octadecadienoate | LMFA01070012 | Lipids and lipid-like molecules | Fatty Acyls | Fatty Acids and Conjugates |
| Dide-O-methyl-4-O-alpha-D-glucopyranosylsimmondsin | HMDB0038329 | Organic oxygen compounds | Organooxygen compounds | Carbohydrates and carbohydrate conjugates |
| Cabbage identification factor 2 | 89422 | Organic acids and derivatives | Carboxylic acids and derivatives | Amino acids, peptides, and analogues |
| 15(S)-15-methyl PGF2α ethyl amide | 64689 | Unclassified | Unclassified | Unclassified |
| Isopropyl citrate | 88388 | Unclassified | Unclassified | Unclassified |
| PC(O-5:0/0:0)[R] | LMGP01060020 | Lipids and lipid-like molecules | Glycerophospholipids | Glycerophosphocholines |
| Vitamin D2 3-glucuronide | LMST03010072 | Lipids and lipid-like molecules | Steroids and steroid derivatives | Vitamin D and derivatives |
| Euchrenone a17 | LMPK12140059 | Lipids and lipid-like molecules | Polyketides | Flavonoids |
| JWH 307 | 85204 | Unclassified | Unclassified | Unclassified |
| Methyl 5-(hydroxymethyl)pyrrolidine-3-carboxylate | HMDB0061678 | Organoheterocyclic compounds | Pyrrolidines | Pyrrolidine carboxylic acids and derivatives |
| Glycylleucine | HMDB0000759 | Organic acids and derivatives | Carboxylic acids and derivatives | Amino acids, peptides, and analogues |
| Ixocarpanolide | HMDB0033899 | Lipids and lipid-like molecules | Steroids and steroid derivatives | Steroid lactones |
| 2,3,4,5-Tetrahydro-6-(5-methyl-2-furanyl)pyridine | HMDB0040015 | Organoheterocyclic compounds | Pyridines and derivatives | Hydropyridines |
| Tolrestat | 2878 | Unclassified | Unclassified | Unclassified |
| Kynurenic acid | HMDB0000715 | Organoheterocyclic compounds | Quinolines and derivatives | Quinoline carboxylic acids |
| Isonocardicin A | 65542 | Unclassified | Unclassified | Unclassified |
| 27-Hydroxyisomangiferolic acid | HMDB0036064 | Lipids and lipid-like molecules | Prenol lipids | Quinone and hydroquinone lipids |
| (+)-Tephrorin A | LMPK12140008 | Lipids and lipid-like molecules | Polyketides | Flavonoids |
| PE(14:0/0:0) | 40777 | Lipids and lipid-like molecules | Glycerophospholipids | Glycerophosphoethanolamines |
| CETRIMONIUM | 43833 | Unclassified | Unclassified | Unclassified |
| 15-Hydroxy-9Z,15Z-octadecadienoic acid | LMFA02000315 | Lipids and lipid-like molecules | Fatty Acyls | Octadecanoids |
| 6-(5-{2-[4,6-dihydroxy-2-methoxy-3-(3-methylbut-2-en-1-yl)phenyl]acetyl}-2,4-dihydroxyphenoxy)-3,4,5-trihydroxyoxane-2-carboxylic acid | HMDB0125482 | Phenylpropanoids and polyketides | Stilbenes | Stilbene glycosides |
| [6]-Gingerdiol 3,5-diacetate | 95053 | Lipids and lipid-like molecules | Fatty Acyls | Fatty alcohol esters |
| Blepharin | HMDB0029344 | Organic oxygen compounds | Organooxygen compounds | Carbohydrates and carbohydrate conjugates |
| Melizame | HMDB0029684 | Organic oxygen compounds | Organooxygen compounds | Ethers |
| Methyldymron | 72561 | Unclassified | Unclassified | Unclassified |
| 6-Hydroxyprotopine | 64365 | Unclassified | Unclassified | Unclassified |
| 3-Methyl-5-propyl-2-furanundecanoic acid | HMDB0061646 | Lipids and lipid-like molecules | Fatty Acyls | Fatty acids and conjugates |
| Homocitrulline | HMDB0000679 | Organic acids and derivatives | Carboxylic acids and derivatives | Amino acids, peptides, and analogues |
| 3-oxo-heptadecanoic acid | LMFA01060109 | Lipids and lipid-like molecules | Fatty Acyls | Fatty Acids and Conjugates |
| 1-Monopalmitin | 24076 | Lipids and lipid-like molecules | Glycerolipids | Monoradylglycerols |
| 2-Methylbutyroylcarnitine | HMDB0000378 | Lipids and lipid-like molecules | Fatty Acyls | Fatty acid esters |
| 3-[4-hydroxy-3-(3-methylbut-2-en-1-yl)phenyl]-2-(sulfooxy)propanoic acid | HMDB0133209 | Phenylpropanoids and polyketides | Phenylpropanoic acids | Unclassified |
| Leukotriene A4-d5 methyl ester | 96383 | Unclassified | Unclassified | Unclassified |
| 5-Azacytidine 5'-triphosphate | 1179 | Unclassified | Unclassified | Unclassified |
| 1-Pentanesulfenothioic acid | HMDB0031160 | Organosulfur compounds | Sulfenyl compounds | Unclassified |
| S-NEPC | 64656 | Unclassified | Unclassified | Unclassified |
| BAY-u9773 | 63071 | Unclassified | Unclassified | Unclassified |
| N-cis-hexadec-9Z-enoyl-L-Homoserine lactone | 64726 | Unclassified | Unclassified | Unclassified |
| Petunidin 3-(6''-acetylglucoside) | LMPK12010356 | Unclassified | Unclassified | Unclassified |
| Caryoptosidic acid | HMDB0034249 | Lipids and lipid-like molecules | Prenol lipids | Terpene glycosides |
| Aminoadipic acid | HMDB0000510 | Organic acids and derivatives | Carboxylic acids and derivatives | Amino acids, peptides, and analogues |
| PROMETRYN | 44589 | Unclassified | Unclassified | Unclassified |
| Desmethyltrimeprazine | 2944 | Unclassified | Unclassified | Unclassified |
| Aminoparathion | HMDB0001504 | Organic acids and derivatives | Organic thiophosphoric acids and derivatives | Thiophosphoric acid esters |
| 4-Hydroxy-3-nitrosobenzamide | 69313 | Unclassified | Unclassified | Unclassified |
| 8-Isoprostane | HMDB0004659 | Hydrocarbons | Saturated hydrocarbons | Cycloalkanes |

**T2 VS CK**

| **Metabolites** | **Compound ID** | **Super Class** | **Class** | **Sub Class** |
| --- | --- | --- | --- | --- |
| Phthalic acid | HMDB0002107 | Benzenoids | Benzene and substituted derivatives | Benzoic acids and derivatives |
| γ- 6(7)-EpODE | 36042 | Unclassified | Unclassified | Unclassified |
| DL-Sulforaphane | 58318 | Unclassified | Unclassified | Unclassified |
| 9,10,13-TriHOME | HMDB0004710 | Lipids and lipid-like molecules | Fatty Acyls | Fatty acids and conjugates |
| 4-Hydroxybenzaldehyde | HMDB0011718 | Organic oxygen compounds | Organooxygen compounds | Carbonyl compounds |
| 6-hydroxysphingosine | 53903 | Lipids and lipid-like molecules | Sphingolipids | Sphingoid bases |
| Dambonitol | HMDB0033942 | Organic oxygen compounds | Organooxygen compounds | Alcohols and polyols |
| C16 Sphinganine | LMSP01040001 | Lipids and lipid-like molecules | Sphingolipids | Sphingoid bases |
| N-Carboxyacetyl-D-phenylalanine | 93703 | Organic acids and derivatives | Carboxylic acids and derivatives | Amino acids, peptides, and analogues |
| (2-{[2-(3,4-dihydroxyphenyl)-5,7-dihydroxy-4-oxo-4H-chromen-3-yl]oxy}-4-hydroxy-5-(hydroxymethyl)oxolan-3-yl)oxidanesulfonic acid | HMDB0132731 | Phenylpropanoids and polyketides | Flavonoids | Flavonoid glycosides |
| 4,4'-Bis(dimethylamino)benzophenone | 72984 | Unclassified | Unclassified | Unclassified |
| 6-O-(3R,4-dihydroxy-2-methylene-butanoyl)-beta-D-glucopyranose | LMSL05000003 | Unclassified | Unclassified | Unclassified |
| Cyclopentanethiol | HMDB0039771 | Organosulfur compounds | Thiols | Alkylthiols |
| 12,13,15-trihydroxy-9E-octadecenoic acid | LMFA01050555 | Lipids and lipid-like molecules | Fatty Acyls | Fatty Acids and Conjugates |
| MG(16:0/0:0/0:0)[rac] | 75555 | Lipids and lipid-like molecules | Glycerolipids | Monoradylglycerols |
| Sphingosine | HMDB0000252 | Organic nitrogen compounds | Organonitrogen compounds | Amines |
| Cinnamoylglycine | HMDB0011621 | Organic acids and derivatives | Carboxylic acids and derivatives | Amino acids, peptides, and analogues |
| Caulerpin | 71556 | Unclassified | Unclassified | Unclassified |
| 3,4-Dihydroxybenzaldehyde | HMDB0059965 | Organic oxygen compounds | Organooxygen compounds | Carbonyl compounds |
| 4-Isothiocyanato-1-butene | HMDB0033867 | Organosulfur compounds | Isothiocyanates | Unclassified |
| 17-hydroxy-linolenic acid | LMFA02000239 | Lipids and lipid-like molecules | Fatty Acyls | Octadecanoids |
| Tyramine | HMDB0000306 | Benzenoids | Benzene and substituted derivatives | Phenethylamines |
| 13S-HOTrE(gamma) | 35514 | Lipids and lipid-like molecules | Fatty Acyls | Octadecanoids |
| Ethyl beta-D-glucopyranoside | HMDB0029968 | Organic oxygen compounds | Organooxygen compounds | Carbohydrates and carbohydrate conjugates |
| Tyrosyl-Glutamine | HMDB0029103 | Organic acids and derivatives | Carboxylic acids and derivatives | Amino acids, peptides, and analogues |
| O-Oxalylhomoserine | 66891 | Unclassified | Unclassified | Unclassified |
| Acetamiprid | 72313 | Unclassified | Unclassified | Unclassified |
| methyl 12,13-epoxy-9,15-octadecadienoate | 74810 | Lipids and lipid-like molecules | Fatty Acyls | Fatty Acids and Conjugates |
| epothilone D | LMPK04000001 | Lipids and lipid-like molecules | Polyketides | Macrolides and lactone polyketides |
| Adenine | HMDB0000034 | Organoheterocyclic compounds | Imidazopyrimidines | Purines and purine derivatives |
| Fensulfothion | 70114 | Unclassified | Unclassified | Unclassified |
| 6-Formylindolo [3,2-B] carbazole | 43424 | Unclassified | Unclassified | Unclassified |
| Gluconic acid | HMDB0000625 | Organic oxygen compounds | Organooxygen compounds | Carbohydrates and carbohydrate conjugates |
| Imidapril | HMDB0041907 | Organic acids and derivatives | Carboxylic acids and derivatives | Amino acids, peptides, and analogues |
| Erythrono-1,4-lactone | HMDB0000349 | Organoheterocyclic compounds | Lactones | Gamma butyrolactones |
| 2,3-Butanediol glucoside | HMDB0040822 | Organic oxygen compounds | Organooxygen compounds | Carbohydrates and carbohydrate conjugates |
| 8-Hydroxy-4,8-dimethyl-4E,9-decadienoic acid | LMFA01050373 | Lipids and lipid-like molecules | Fatty Acyls | Fatty Acids and Conjugates |
| alpha-(p-Methoxyphenyl)-6-methyl-2-pyridineacrylic acid | 70613 | Unclassified | Unclassified | Unclassified |
| Allopurinol | HMDB0014581 | Organoheterocyclic compounds | Pyrazolopyrimidines | Pyrazolo[3,4-d]pyrimidines |
| Homocitrulline | HMDB0000679 | Organic acids and derivatives | Carboxylic acids and derivatives | Amino acids, peptides, and analogues |
| Inulobiose | HMDB0029898 | Organic oxygen compounds | Organooxygen compounds | Carbohydrates and carbohydrate conjugates |
| PC(18:1(9E)/0:0)[U] | 46689 | Unclassified | Unclassified | Unclassified |
| 3,4,5-trihydroxy-6-{[4-hydroxy-5-(3-hydroxyphenyl)pentanoyl]oxy}oxane-2-carboxylic acid | HMDB0127755 | Organic oxygen compounds | Organooxygen compounds | Carbohydrates and carbohydrate conjugates |
| Tryptophyl-Cysteine | HMDB0029080 | Organic acids and derivatives | Carboxylic acids and derivatives | Amino acids, peptides, and analogues |
| 5'-Carboxy-gamma-chromanol | HMDB0012799 | Organoheterocyclic compounds | Benzopyrans | 1-benzopyrans |
| LysoPA(0:0/18:2(9Z,12Z)) | HMDB0007852 | Lipids and lipid-like molecules | Glycerophospholipids | Glycerophosphates |
| gamma-Glutamylleucine | HMDB0011171 | Organic acids and derivatives | Carboxylic acids and derivatives | Amino acids, peptides, and analogues |
| POB-PS | LMGP20040017 | Lipids and lipid-like molecules | Glycerophospholipids | Oxidized glycerophospholipids |
| 6-({[3,4-dihydroxy-4-(hydroxymethyl)oxolan-2-yl]oxy}methyl)oxane-2,3,4,5-tetrol | HMDB0140855 | Organic oxygen compounds | Organooxygen compounds | Carbohydrates and carbohydrate conjugates |
| 10-HODTA methyl ester | 65404 | Unclassified | Unclassified | Unclassified |
| 2-Methylhippuric acid | HMDB0011723 | Benzenoids | Benzene and substituted derivatives | Benzoic acids and derivatives |
| Taxifolin | HMDB0125371 | Phenylpropanoids and polyketides | Flavonoids | Flavans |
| 3,4-dihydroxy-2-(5-hydroxy-3,7-dimethylocta-2,6-dien-1-yl)benzoic acid | HMDB0134979 | Benzenoids | Benzene and substituted derivatives | Benzoic acids and derivatives |
| Terbufos | 72471 | Unclassified | Unclassified | Unclassified |
| Isopropyl citrate | 88388 | Unclassified | Unclassified | Unclassified |
| 5-Methoxyindoleacetate | HMDB0004096 | Organoheterocyclic compounds | Indoles and derivatives | Indolyl carboxylic acids and derivatives |
| Alpha-Linolenic acid | HMDB0001388 | Lipids and lipid-like molecules | Fatty Acyls | Lineolic acids and derivatives |
| 16-B1-PhytoP | LMFA02030004 | Lipids and lipid-like molecules | Fatty Acyls | Octadecanoids |
| Uracil | HMDB0000300 | Organoheterocyclic compounds | Diazines | Pyrimidines and pyrimidine derivatives |
| Araliacerebroside | HMDB0033621 | Lipids and lipid-like molecules | Sphingolipids | Glycosphingolipids |
| 1-(2-methoxy-21Z-octacosenyl)-sn-glycero-3-phosphoserine | LMGP03060029 | Lipids and lipid-like molecules | Glycerophospholipids | Glycerophosphoserines |
| 2-N,6-N-Bis(2,3-dihydroxybenzoyl)-L-lysine | 66403 | Unclassified | Unclassified | Unclassified |
| 3-carboxy-4-methyl-5-(4-hydroxypentyl)-2-furanpropanoic acid | LMFA01150068 | Lipids and lipid-like molecules | Fatty Acyls | Fatty Acids and Conjugates |
| Colnelenic acid | LMFA10000002 | Lipids and lipid-like molecules | Fatty Acyls | Fatty acids and conjugates |
| Harderoporphyrin | HMDB0000683 | Organoheterocyclic compounds | Tetrapyrroles and derivatives | Porphyrins |
| 2-Phenylethyl beta-D-glucopyranoside | HMDB0029819 | Organic oxygen compounds | Organooxygen compounds | Carbohydrates and carbohydrate conjugates |
| L-Isoleucine | HMDB0000172 | Organic acids and derivatives | Carboxylic acids and derivatives | Amino acids, peptides, and analogues |
| {3,8,15-trihydroxy-16,17-dimethoxy-9-oxotricyclo[12.3.1.1²,⁶]nonadeca-1(17),2,4,6(19),14(18),15-hexaen-7-yl}oxidanesulfonic acid | HMDB0133716 | Phenylpropanoids and polyketides | Diarylheptanoids | Cyclic diarylheptanoids |
| 4-fumarylacetoacetic acid | LMFA01170066 | Organic acids and derivatives | Keto acids and derivatives | Medium-chain keto acids and derivatives |
| 2-amino-4-({1-[(carboxymethyl)-C-hydroxycarbonimidoyl]-2-[(4-{3,5-dihydroxy-2,2-dimethyl-8-oxo-6-propyl-2H,3H,4H,8H-pyrano[3,2-g]chromen-10-yl}-4-oxobutyl)sulfanyl]ethyl}-C-hydroxycarbonimidoyl)butanoic acid | HMDB0130118 | Organic acids and derivatives | Carboxylic acids and derivatives | Amino acids, peptides, and analogues |
| 2-Dodecylbenzenesulfonic acid | HMDB0031031 | Benzenoids | Benzene and substituted derivatives | Benzenesulfonic acids and derivatives |
| Guanine | HMDB0000132 | Organoheterocyclic compounds | Imidazopyrimidines | Purines and purine derivatives |
| 6-[4-carboxy-2-hydroxy-6-(3,4,5-trihydroxybenzoyloxy)phenoxy]-3,4,5-trihydroxyoxane-2-carboxylic acid | HMDB0128328 | Phenylpropanoids and polyketides | Tannins | Hydrolyzable tannins |
| Panaxydol | LMFA05000028 | Lipids and lipid-like molecules | Fatty Acyls | Fatty alcohols |
| (S)-Nerolidol 3-O-[a-L-rhamnopyranosyl-(1->4)-a-L-rhamnopyranosyl-(1->6)-b-D-glucopyranoside] | HMDB0040846 | Organic oxygen compounds | Organooxygen compounds | Carbohydrates and carbohydrate conjugates |
| 4-hydroxy-butyric acid | LMFA01050006 | Lipids and lipid-like molecules | Fatty Acyls | Fatty acids and conjugates |
| N6-beta-Aspartyllysine | HMDB0004985 | Organic acids and derivatives | Carboxylic acids and derivatives | Amino acids, peptides, and analogues |
| PA(0:0/18:2(9Z,12Z)) | LMGP10050044 | Lipids and lipid-like molecules | Glycerophospholipids | Glycerophosphates |
| 1-(3,4-Dihydroxyphenyl)-1-decene-3,5-dione | 64193 | Unclassified | Unclassified | Unclassified |
| 9-oxo-13-hydroxy-11-octadecenoic acid | LMFA02000248 | Lipids and lipid-like molecules | Fatty Acyls | Octadecanoids |
| Cellulose, microcrystalline | HMDB0032197 | Organic oxygen compounds | Organooxygen compounds | Carbohydrates and carbohydrate conjugates |
| 2-(4-Methyl-5-thiazolyl)ethyl butanoate | HMDB0032418 | Organoheterocyclic compounds | Azoles | Thiazoles |
| 5-Butyltetrahydro-2-oxo-3-furancarboxylic acid | HMDB0030992 | Organoheterocyclic compounds | Lactones | Gamma butyrolactones |
| PON-PA | LMGP20070013 | Lipids and lipid-like molecules | Glycerophospholipids | Oxidized glycerophospholipids |
| Valyl-Leucine | HMDB0029131 | Organic acids and derivatives | Carboxylic acids and derivatives | Amino acids, peptides, and analogues |
| 2-(4-Hydroxyphenyl)naphthalic anhydride | HMDB0041121 | Benzenoids | Naphthalenes | Phenylnaphthalenes |
| S-(1,2-DICARBOXYETHYL)GLUTATHIONE | 44314 | Unclassified | Unclassified | Unclassified |
| 13R-HODE | 74643 | Lipids and lipid-like molecules | Fatty Acyls | Octadecanoids |
| 4-Hydroxy-2-oxoglutaric acid | HMDB0002070 | Organic acids and derivatives | Keto acids and derivatives | Gamma-keto acids and derivatives |
| Starch acetate | 86570 | Organic acids and derivatives | Carboxylic acids and derivatives | Tricarboxylic acids and derivatives |
| beta-Glucogallin | HMDB0038728 | Phenylpropanoids and polyketides | Tannins | Unclassified |
| 3'-Glucosyl-2',4',6'-trihydroxyacetophenone | HMDB0040621 | Organic oxygen compounds | Organooxygen compounds | Carbohydrates and carbohydrate conjugates |
| 2-hydroxyhexadecanoic acid | 34495 | Lipids and lipid-like molecules | Fatty Acyls | Fatty acids and conjugates |
| Lentialexin | LMFA05000672 | Lipids and lipid-like molecules | Fatty Acyls | Fatty alcohols |
| 6-[5-({[3,5-dihydroxy-2-(hydroxymethyl)-6-(3,4,5-trihydroxybenzoyloxy)oxan-4-yl]oxy}carbonyl)-2,3-dihydroxyphenoxy]-3,4,5-trihydroxyoxane-2-carboxylic acid | HMDB0133127 | Phenylpropanoids and polyketides | Tannins | Hydrolyzable tannins |
| Cytochalasin Npho | HMDB0035367 | Alkaloids and derivatives | Cytochalasans | Unclassified |
| 6-{[5-(3,4-dihydroxyphenyl)pentanoyl]oxy}-3,4,5-trihydroxyoxane-2-carboxylic acid | HMDB0127759 | Organic oxygen compounds | Organooxygen compounds | Carbohydrates and carbohydrate conjugates |
| 7-O-Methyltectorigenin 4'-O-gentiobioside | LMPK12050377 | Lipids and lipid-like molecules | Polyketides | Flavonoids |
| Nimesulide | 44341 | Unclassified | Unclassified | Unclassified |
| N-Undecylbenzenesulfonic acid | HMDB0032549 | Benzenoids | Benzene and substituted derivatives | Benzenesulfonic acids and derivatives |
| 9R-hydroxy-10E,12E-octadecadienoic acid, methyl ester | LMFA01050549 | Lipids and lipid-like molecules | Fatty Acyls | Fatty Acids and Conjugates |
| Leucyl-Aspartate | 85829 | Organic acids and derivatives | Carboxylic acids and derivatives | Amino acids, peptides, and analogues |
| Adenosine | HMDB0000050 | Nucleosides, nucleotides, and analogues | Purine nucleosides | Unclassified |
| 2-Methyl-1-methylthio-2-butene | HMDB0032411 | Organosulfur compounds | Thioethers | Dialkylthioethers |
| 1-Nitro-5,6-dihydroxy-dihydronaphthalene | HMDB0060328 | Benzenoids | Naphthalenes | Nitronaphthalenes |
| 12,13-dihydroxy-11-methoxy-9-octadecenoic acid | LMFA01080006 | Lipids and lipid-like molecules | Fatty Acyls | Fatty Acids and Conjugates |
| (1R,2R)-3-[(1,2-Dihydro-2-hydroxy-1-naphthalenyl)thio]-2-oxopropanoic acid | 70331 | Unclassified | Unclassified | Unclassified |
| 7-Methylinosine | HMDB0003950 | Nucleosides, nucleotides, and analogues | Purine nucleosides | Unclassified |
| Gingerglycolipid A | HMDB0041093 | Lipids and lipid-like molecules | Glycerolipids | Glycosylglycerols |
| PA(16:0/0:0) | 40944 | Lipids and lipid-like molecules | Glycerophospholipids | Glycerophosphates |
| 3,11,12-Trihydroxy-1(10)-spirovetiven-2-one | HMDB0038154 | Lipids and lipid-like molecules | Prenol lipids | Sesquiterpenoids |
| Sodium folinate | 71887 | Unclassified | Unclassified | Unclassified |
| PA(18:1(11Z)/15:0) | HMDB0114898 | Lipids and lipid-like molecules | Glycerophospholipids | Glycerophosphates |
| Sphinganine | HMDB0000269 | Organic nitrogen compounds | Organonitrogen compounds | Amines |
| 2,5-Dioxopentanoate | HMDB0060365 | Organic acids and derivatives | Keto acids and derivatives | Short-chain keto acids and derivatives |
| Mirtazapine | HMDB0014514 | Organoheterocyclic compounds | Piperazinoazepines | Unclassified |
| PA(18:1(9Z)/0:0) | LMGP10050008 | Lipids and lipid-like molecules | Glycerophospholipids | Glycerophosphates |
| 4-Dodecylbenzenesulfonic Acid | HMDB0059915 | Benzenoids | Benzene and substituted derivatives | Benzenesulfonic acids and derivatives |
| Azidocillin | 85583 | Organoheterocyclic compounds | Lactams | Beta lactams |
| Ligustilide | HMDB0034277 | Organoheterocyclic compounds | Isobenzofurans | Unclassified |
| Bilastine | HMDB0240232 | Organoheterocyclic compounds | Benzimidazoles | Unclassified |
| Convicine | 66969 | Unclassified | Unclassified | Unclassified |
| Asymmetric dimethylarginine | HMDB0001539 | Organic acids and derivatives | Carboxylic acids and derivatives | Amino acids, peptides, and analogues |
| Uridine | HMDB0000296 | Nucleosides, nucleotides, and analogues | Pyrimidine nucleosides | Unclassified |
| 6-{[3-(2H-1,3-benzodioxol-5-yl)-3-oxopropanoyl]oxy}-3,4,5-trihydroxyoxane-2-carboxylic acid | HMDB0129411 | Organic oxygen compounds | Organooxygen compounds | Carbohydrates and carbohydrate conjugates |
| 1-NBD-decanoyl-2-decanoyl-sn-Glycerol | 62997 | Unclassified | Unclassified | Unclassified |
| PC(19:1(9Z)/0:0) | LMGP01050130 | Lipids and lipid-like molecules | Glycerophospholipids | Glycerophosphocholines |
| (9R,13R)-10-oxo-11-phytoenoic acid | LMFA02010002 | Lipids and lipid-like molecules | Fatty Acyls | Octadecanoids |
| Umbelliferone | HMDB0029865 | Phenylpropanoids and polyketides | Coumarins and derivatives | Hydroxycoumarins |
| 8-nonenoylglycine | HMDB0094813 | Organic acids and derivatives | Carboxylic acids and derivatives | Amino acids, peptides, and analogues |
| 3,4,5-trihydroxy-6-({7-oxo-7H-furo[3,2-g]chromen-4-yl}oxy)oxane-2-carboxylic acid | HMDB0129424 | Organic oxygen compounds | Organooxygen compounds | Carbohydrates and carbohydrate conjugates |
| Cyclodopa glucoside | HMDB0029833 | Organic oxygen compounds | Organooxygen compounds | Carbohydrates and carbohydrate conjugates |
| 1-Oleoyl Lysophosphatidic Acid (sodium salt) | 5432 | Unclassified | Unclassified | Unclassified |
| 10-amino-decanoic acid | LMFA01100002 | Lipids and lipid-like molecules | Fatty Acyls | Fatty Acids and Conjugates |
| Gingerglycolipid B | HMDB0041094 | Lipids and lipid-like molecules | Glycerolipids | Glycosylglycerols |
| N-(16,16-dimethy-5Z,8Z,11Z,14Z-docosatetraenoyl)-ethanolamine | LMFA08040018 | Lipids and lipid-like molecules | Fatty Acyls | Fatty amides |
| LysoPA(0:0/16:0) | HMDB0007849 | Lipids and lipid-like molecules | Glycerophospholipids | Glycerophosphates |
| 6-Hydroxy-2-bornanone glucoside | HMDB0034558 | Lipids and lipid-like molecules | Prenol lipids | Terpene glycosides |
| Oleoyl Ethanolamide | 24103 | Organic nitrogen compounds | Organonitrogen compounds | Amines |
| Xanthosine | HMDB0000299 | Nucleosides, nucleotides, and analogues | Purine nucleosides | Unclassified |
| 11-dehydro-TXB2-d4 | 45968 | Unclassified | Unclassified | Unclassified |
| D-Glucuronic acid | HMDB0000127 | Organic oxygen compounds | Organooxygen compounds | Carbohydrates and carbohydrate conjugates |
| 2-Hydroxyenterodiol | HMDB0041649 | Lignans, neolignans and related compounds | Dibenzylbutane lignans | Dibenzylbutanediol lignans |
| 9S-HpOTrE | LMFA02000018 | Lipids and lipid-like molecules | Fatty Acyls | Octadecanoids |
| Lusitanicoside | HMDB0034120 | Organic oxygen compounds | Organooxygen compounds | Carbohydrates and carbohydrate conjugates |
| Avermectin B1b monosaccharide | 63689 | Unclassified | Unclassified | Unclassified |
| N-(4-aminobutyl)-3-(4-hydroxyphenyl)propanimidic acid | HMDB0139525 | Benzenoids | Phenols | 1-hydroxy-2-unsubstituted benzenoids |
| Gamma-glutamyl-Isoleucine | 86044 | Unclassified | Unclassified | Unclassified |
| Valyl-Phenylalanine | HMDB0029134 | Organic acids and derivatives | Carboxylic acids and derivatives | Amino acids, peptides, and analogues |
| Polyethylene, oxidized | HMDB0032472 | Organic acids and derivatives | Keto acids and derivatives | Medium-chain keto acids and derivatives |
| Citrusin A | HMDB0039230 | Lignans, neolignans and related compounds | Lignan glycosides | Unclassified |
| Aspartame | HMDB0001894 | Organic acids and derivatives | Carboxylic acids and derivatives | Amino acids, peptides, and analogues |
| lysophosphatidic acid | LMGP10050045 | Lipids and lipid-like molecules | Glycerophospholipids | Glycerophosphates |
| 3-Hydroxybutyric acid | HMDB0000357 | Organic acids and derivatives | Hydroxy acids and derivatives | Beta hydroxy acids and derivatives |
| Trans-Hexa-dec-2-enoic acid | HMDB0010735 | Lipids and lipid-like molecules | Fatty Acyls | Fatty acids and conjugates |
| 4-Methoxyglucobrassicin | 66963 | Unclassified | Unclassified | Unclassified |
| 1-O-(3R,4-dihydroxy-2-methylene-butanoyl)-beta-D-glucopyranose | LMFA13010038 | Lipids and lipid-like molecules | Fatty Acyls | Fatty acyl glycosides |
| 16-epi-16-D1t-PhytoP | LMFA02030036 | Lipids and lipid-like molecules | Fatty Acyls | Octadecanoids |
| O-Desmethyltramadol glucuronide | HMDB0060856 | Organic oxygen compounds | Organooxygen compounds | Carbohydrates and carbohydrate conjugates |
| Pipermethystine | HMDB0033486 | Organoheterocyclic compounds | Pyridines and derivatives | Hydropyridines |
| Stearyl monoglyceridyl citrate | 88504 | Unclassified | Unclassified | Unclassified |
| Garcinia lactone dibutyl ester | HMDB0040462 | Organic acids and derivatives | Carboxylic acids and derivatives | Tricarboxylic acids and derivatives |
| Pyroglutamylvaline | HMDB0094651 | Organic acids and derivatives | Carboxylic acids and derivatives | Amino acids, peptides, and analogues |
| Threoninyl-Leucine | HMDB0029065 | Organic acids and derivatives | Carboxylic acids and derivatives | Amino acids, peptides, and analogues |
| OON-PA | LMGP20070023 | Lipids and lipid-like molecules | Glycerophospholipids | Oxidized glycerophospholipids |
| PE(14:0/24:0) | HMDB0008848 | Lipids and lipid-like molecules | Glycerophospholipids | Glycerophosphoethanolamines |
| Glucose 6-phosphate | HMDB0001401 | Organic oxygen compounds | Organooxygen compounds | Carbohydrates and carbohydrate conjugates |
| PE(17:1(9Z)/0:0) | 46714 | Lipids and lipid-like molecules | Glycerophospholipids | Glycerophosphoethanolamines |
| 6-(butanoyloxy)-3,4,5-trihydroxyoxane-2-carboxylic acid | HMDB0130127 | Organic oxygen compounds | Organooxygen compounds | Carbohydrates and carbohydrate conjugates |
| L-Phenylalanine | HMDB0000159 | Organic acids and derivatives | Carboxylic acids and derivatives | Amino acids, peptides, and analogues |
| 3-Methyl-5-propyl-2-furanundecanoic acid | HMDB0061646 | Lipids and lipid-like molecules | Fatty Acyls | Fatty acids and conjugates |
| 1,6-Digalloyl-beta-D-glucopyranose | HMDB0039179 | Phenylpropanoids and polyketides | Tannins | Unclassified |
| Arginyl-Proline | HMDB0028717 | Organic acids and derivatives | Carboxylic acids and derivatives | Amino acids, peptides, and analogues |
| MG(0:0/18:1(9Z)/0:0) | 62321 | Lipids and lipid-like molecules | Glycerolipids | Monoradylglycerols |
| (R)-(Homo)3-citrate | 71255 | Unclassified | Unclassified | Unclassified |
| Isoleucyl-Glutamine | HMDB0028905 | Organic acids and derivatives | Carboxylic acids and derivatives | Amino acids, peptides, and analogues |
| CAY10512 | 45185 | Unclassified | Unclassified | Unclassified |
| 2-benzyl-2-hydroxybutanedioic acid | HMDB0142172 | Phenylpropanoids and polyketides | Phenylpropanoic acids | Unclassified |
| 6-Thioinosinic acid | HMDB0060791 | Nucleosides, nucleotides, and analogues | Purine nucleosides | Unclassified |
| DG(8:0/8:0/0:0) | HMDB0116368 | Lipids and lipid-like molecules | Glycerolipids | Diradylglycerols |
| D-glycero-L-galacto-Octulose | HMDB0029954 | Organic oxygen compounds | Organooxygen compounds | Carbohydrates and carbohydrate conjugates |
| 15-hydroxy stearic acid | LMFA02000133 | Lipids and lipid-like molecules | Fatty Acyls | Octadecanoids |
| Gluconasturtiin | HMDB0038423 | Organic oxygen compounds | Organooxygen compounds | Carbohydrates and carbohydrate conjugates |
| 4-({2-[(3-{2-[2-(acetyloxy)propan-2-yl]-7-oxo-2H,3H,7H-furo[3,2-g]chromen-6-yl}-1-hydroxy-3-methylbutan-2-yl)sulfanyl]-1-[(carboxymethyl)-C-hydroxycarbonimidoyl]ethyl}-C-hydroxycarbonimidoyl)-2-aminobutanoic acid | HMDB0130306 | Organic acids and derivatives | Carboxylic acids and derivatives | Amino acids, peptides, and analogues |
| 9R,10S-EpOME | 43441 | Lipids and lipid-like molecules | Fatty Acyls | Octadecanoids |
| LysoPC(18:1(9Z)) | HMDB0002815 | Lipids and lipid-like molecules | Glycerophospholipids | Glycerophosphocholines |
| (±)9-HpODE | 64785 | Unclassified | Unclassified | Unclassified |
| Ticarcillin | HMDB0015545 | Organic acids and derivatives | Carboxylic acids and derivatives | Amino acids, peptides, and analogues |
| Taraxacolide 1-O-b-D-glucopyranoside | HMDB0035610 | Lipids and lipid-like molecules | Prenol lipids | Terpene lactones |
| N-Desmethyleletriptan | HMDB0013919 | Organoheterocyclic compounds | Indoles and derivatives | Indoles |
| 12-Hydroxy-8,10-octadecadienoic acid | HMDB0029998 | Lipids and lipid-like molecules | Fatty Acyls | Lineolic acids and derivatives |
| NVP-BEZ235 | 64764 | Unclassified | Unclassified | Unclassified |
| 4-Oxo-1-(3-pyridyl)-1-butanone | HMDB0062406 | Organic oxygen compounds | Organooxygen compounds | Carbonyl compounds |
| L-Lyxonate | HMDB0060255 | Organic oxygen compounds | Organooxygen compounds | Carbohydrates and carbohydrate conjugates |
| 2-Methoxy-3-(4-methoxyphenyl)propanoic acid | HMDB0039428 | Phenylpropanoids and polyketides | Phenylpropanoic acids | Unclassified |
| PE(18:1(9Z)/0:0) | 40778 | Lipids and lipid-like molecules | Glycerophospholipids | Glycerophosphoethanolamines |
| PA(0:0/18:1(9Z)) | LMGP10050014 | Lipids and lipid-like molecules | Glycerophospholipids | Glycerophosphates |
| 3-Deoxy-D-glycero-D-galacto-2-nonulosonic acid | HMDB0000425 | Organic oxygen compounds | Organooxygen compounds | Carbohydrates and carbohydrate conjugates |
| Serylisoleucine | HMDB0029042 | Organic acids and derivatives | Carboxylic acids and derivatives | Amino acids, peptides, and analogues |
| PE(P-16:0/0:0) | 46719 | Lipids and lipid-like molecules | Glycerophospholipids | Glycerophosphoethanolamines |
| Metaproterenol 3-O-sulfate | 1178 | Unclassified | Unclassified | Unclassified |
| Morphinan-3-ol, hydrogen sulfate | 1979 | Unclassified | Unclassified | Unclassified |
| Aspoxicillin | 69555 | Unclassified | Unclassified | Unclassified |
| N-stearoyl glutamine | LMFA08020130 | Lipids and lipid-like molecules | Fatty Acyls | Fatty amides |
| 7,8-Dihydroneopterin | HMDB0002275 | Organoheterocyclic compounds | Pteridines and derivatives | Pterins and derivatives |
| Glutamylisoleucine | HMDB0028822 | Organic acids and derivatives | Carboxylic acids and derivatives | Amino acids, peptides, and analogues |
| 7-Methylxanthine | HMDB0001991 | Organoheterocyclic compounds | Imidazopyrimidines | Purines and purine derivatives |
| LysoPC(0:0/16:0) | HMDB0240262 | Lipids and lipid-like molecules | Glycerophospholipids | Glycerophosphocholines |
| S-(2,5-Dimethyl-3-furanyl) 2-furancarbothioate | HMDB0039585 | Organoheterocyclic compounds | Furans | Furoic acid and derivatives |
| 3,4,5-trihydroxy-6-(2-hydroxyethoxy)oxane-2-carboxylic acid | HMDB0131294 | Organic oxygen compounds | Organooxygen compounds | Carbohydrates and carbohydrate conjugates |
| 2-Phenylaminoadenosine | HMDB0001069 | Nucleosides, nucleotides, and analogues | Purine nucleosides | Unclassified |
| 3',4'-Methylenedioxyfurano[2'',3'':6,7]aurone | LMPK12130007 | Unclassified | Unclassified | Unclassified |
| Nandrolone | HMDB0002725 | Lipids and lipid-like molecules | Steroids and steroid derivatives | Estrane steroids |
| Tyrosyl-Alanine | HMDB0029098 | Organic acids and derivatives | Carboxylic acids and derivatives | Amino acids, peptides, and analogues |
| Valyl-Lysine | HMDB0029132 | Organic acids and derivatives | Carboxylic acids and derivatives | Amino acids, peptides, and analogues |
| Spironolactone | HMDB0014565 | Lipids and lipid-like molecules | Steroids and steroid derivatives | Steroid lactones |
| 5-(3-methoxyphenyl)-4-(sulfooxy)pentanoic acid | HMDB0127785 | Lipids and lipid-like molecules | Fatty Acyls | Fatty acids and conjugates |
| 4-(N-Maleimido)benzyltrimethylammonium iodide | 69630 | Unclassified | Unclassified | Unclassified |
| beta-D-Xylopyranosyl-(1->5)-alpha-L-arabinofuranosyl-(1->5)-L-arabinose | HMDB0038860 | Organic oxygen compounds | Organooxygen compounds | Carbohydrates and carbohydrate conjugates |
| {[6-(5,7-dihydroxy-4-oxo-3,4-dihydro-2H-1-benzopyran-2-yl)-7-hydroxy-2-(4-methylpent-3-en-1-yl)-2H-chromen-2-yl]methoxy}sulfonic acid | HMDB0132814 | Phenylpropanoids and polyketides | Flavonoids | Pyranoflavonoids |
| Fumaric acid | HMDB0000134 | Organic acids and derivatives | Carboxylic acids and derivatives | Dicarboxylic acids and derivatives |
| 2,3,4-Trihydroxybenzylhydrazide | 1291 | Unclassified | Unclassified | Unclassified |
| 10-hydroxy-8E-Decene-2,4,6-triynoic acid | LMFA01030714 | Lipids and lipid-like molecules | Fatty Acyls | Fatty Acids and Conjugates |
| Gingerglycolipid C | HMDB0041095 | Lipids and lipid-like molecules | Glycerolipids | Glycosylglycerols |
| 9(10)-EpODE | HMDB0010220 | Lipids and lipid-like molecules | Fatty Acyls | Fatty acids and conjugates |
| [10]-Shogaol | 87698 | Benzenoids | Phenols | Methoxyphenols |
| alpha-N-(3-hydroxyhexadecanoyl) L-ornithine | LMFA08020242 | Lipids and lipid-like molecules | Fatty Acyls | Fatty amides |
| Bifenazate | 72390 | Unclassified | Unclassified | Unclassified |
| (±)-(Z)-2-(5-Tetradecenyl)cyclobutanone | HMDB0037543 | Organic oxygen compounds | Organooxygen compounds | Carbonyl compounds |
| 2-methyl-dodecanedioic acid | LMFA01170010 | Lipids and lipid-like molecules | Fatty Acyls | Fatty Acids and Conjugates |
| Zalcitabine diphosphate | 3064 | Unclassified | Unclassified | Unclassified |
| Olanzapine | HMDB0005012 | Organoheterocyclic compounds | Benzodiazepines | Unclassified |
| PG(18:1(9Z)/0:0) | 40878 | Lipids and lipid-like molecules | Glycerophospholipids | Glycerophosphoglycerols |
| PS(P-20:0/0:0) | LMGP03070001 | Lipids and lipid-like molecules | Glycerophospholipids | Glycerophosphoserines |
| AVOCADYNONE ACETATE | 43724 | Unclassified | Unclassified | Unclassified |
| PE-Cer(d14:2(4E,6E)/24:1(15Z)) | LMSP03020037 | Lipids and lipid-like molecules | Sphingolipids | Phosphosphingolipids |
| Magnesium Sulfate | 85410 | Unclassified | Unclassified | Unclassified |
| (3,4,5,6-tetrahydroxyoxan-2-yl)methyl 3-(4-hydroxy-3-methoxyphenyl)prop-2-enoate | HMDB0125101 | Phenylpropanoids and polyketides | Cinnamic acids and derivatives | Hydroxycinnamic acids and derivatives |
| (3alphaOH,20S,24S)-3,19:20,24-Diepoxydammarane-3,25-diol | HMDB0034683 | Lipids and lipid-like molecules | Prenol lipids | Terpene glycosides |
| 8Z-decen-4,6-diynoic acid | LMFA01050421 | Lipids and lipid-like molecules | Fatty Acyls | Fatty Acids and Conjugates |
| 9,12,15-octadecatrienal | LMFA06000102 | Lipids and lipid-like molecules | Fatty Acyls | Fatty aldehydes |
| Apholate | 73037 | Unclassified | Unclassified | Unclassified |
| (±)-Glycerol 1,2-diacetate | HMDB0031712 | Lipids and lipid-like molecules | Glycerolipids | Diradylglycerols |
| gamma-Glutamylasparagine | HMDB0029144 | Organic acids and derivatives | Carboxylic acids and derivatives | Amino acids, peptides, and analogues |
| Latrunculin B | 45288 | Unclassified | Unclassified | Unclassified |
| Cortisol | HMDB0000063 | Lipids and lipid-like molecules | Steroids and steroid derivatives | Hydroxysteroids |
| PC(O-5:0/0:0)[R] | LMGP01060020 | Lipids and lipid-like molecules | Glycerophospholipids | Glycerophosphocholines |
| Citrusin C | HMDB0038708 | Organic oxygen compounds | Organooxygen compounds | Carbohydrates and carbohydrate conjugates |
| LysoPC(14:1(9Z)) | 61690 | Lipids and lipid-like molecules | Glycerophospholipids | Glycerophosphocholines |
| famprofazone | 96167 | Organoheterocyclic compounds | Azoles | Pyrazoles |
| Diethylhexyl adipate | HMDB0040270 | Lipids and lipid-like molecules | Fatty Acyls | Fatty acid esters |
| N-Deschlorobenzoyl indomethacin | HMDB0013988 | Organoheterocyclic compounds | Indoles and derivatives | Indolyl carboxylic acids and derivatives |
| 4-Deoxy-beta-D-gluc-4-enuronosyl-(1,3)-N-acetyl-D-galactosamine 4-sulfate | 66269 | Unclassified | Unclassified | Unclassified |
| (R)-Mevalonic acid-5-pyrophosphate | HMDB0001981 | Organic oxygen compounds | Organic oxoanionic compounds | Organic pyrophosphates |
| [6]-Gingerdiol 3-acetate | 95051 | Lipids and lipid-like molecules | Fatty Acyls | Fatty alcohol esters |
| Arginyl-Histidine | HMDB0028711 | Organic acids and derivatives | Carboxylic acids and derivatives | Amino acids, peptides, and analogues |
| Neriantogenin | HMDB0030044 | Lipids and lipid-like molecules | Steroids and steroid derivatives | Steroid lactones |
| trans-3-Hydroxycotinine glucuronide | 6080 | Organic oxygen compounds | Organooxygen compounds | Carbohydrates and carbohydrate conjugates |
| Europine | 68230 | Unclassified | Unclassified | Unclassified |
| Dihomomethionine | 64496 | Unclassified | Unclassified | Unclassified |
| 11-trans-LTE4 | LMFA03020022 | Lipids and lipid-like molecules | Fatty Acyls | Eicosanoids |
| 12-Dehydroporson | 95815 | Lipids and lipid-like molecules | Fatty Acyls | Fatty acyl glycosides |
| Phenylalanyl-Threonine | HMDB0029005 | Organic acids and derivatives | Carboxylic acids and derivatives | Amino acids, peptides, and analogues |
| N-(5Z,8Z,11Z,14Z-docosatetraenoyl)-ethanolamine | LMFA08040021 | Lipids and lipid-like molecules | Fatty Acyls | Fatty amides |
| 2-Oxo-6-methylthiohexanoic acid | 64495 | Unclassified | Unclassified | Unclassified |
| N2'-Acetylgentamicin C1a | 65995 | Unclassified | Unclassified | Unclassified |
| Crotoxyphos | 72730 | Unclassified | Unclassified | Unclassified |
| (E)-4-(6-methyl-1,2-dithiin-3-yl)but-1-en-3-yn-1-ol | LMFA12000367 | Lipids and lipid-like molecules | Fatty Acyls | Oxygenated hydrocarbons |
| Di-n-octyl phthalate | 69894 | Unclassified | Unclassified | Unclassified |
| Nitrendipine | HMDB0015187 | Organoheterocyclic compounds | Pyridines and derivatives | Hydropyridines |
| Leucyl-Isoleucine | HMDB0028932 | Organic acids and derivatives | Carboxylic acids and derivatives | Amino acids, peptides, and analogues |
| Methyl helianthenoate F glucoside | HMDB0040797 | Lipids and lipid-like molecules | Fatty Acyls | Fatty acyl glycosides |
| N-(3-carboxypropanoyl)-N-hydroxycadaverine | LMFA08020187 | Lipids and lipid-like molecules | Fatty Acyls | Fatty amides |
| Corchorusoside A | HMDB0032823 | Lipids and lipid-like molecules | Steroids and steroid derivatives | Steroid lactones |
| Valyl-Arginine | HMDB0029121 | Organic acids and derivatives | Carboxylic acids and derivatives | Amino acids, peptides, and analogues |
| 6-(5-ethyl-2,3-dihydroxyphenoxy)-3,4,5-trihydroxyoxane-2-carboxylic acid | HMDB0128028 | Organic oxygen compounds | Organooxygen compounds | Carbohydrates and carbohydrate conjugates |
| Vanillic acid | HMDB0000484 | Benzenoids | Benzene and substituted derivatives | Benzoic acids and derivatives |
| 2-Hydroxy-6-ketononatrienedioate | 63525 | Unclassified | Unclassified | Unclassified |
| PA(20:1(11Z)/0:0) | LMGP10050026 | Lipids and lipid-like molecules | Glycerophospholipids | Glycerophosphates |
| 3-Hydroxymethyltriazolopthalazinone | 3167 | Unclassified | Unclassified | Unclassified |
| Hydroxyhexanoycarnitine | LMFA07070072 | Lipids and lipid-like molecules | Fatty Acyls | Fatty acid esters |
| 11-oxo-undeca-5,8-dienoic acid | LMFA01060220 | Lipids and lipid-like molecules | Fatty Acyls | Fatty Acids and Conjugates |
| N-lactoyl-Tryptophan | HMDB0062178 | Organic acids and derivatives | Carboxylic acids and derivatives | Amino acids, peptides, and analogues |
| PS(P-16:0/0:0) | LMGP03070003 | Lipids and lipid-like molecules | Glycerophospholipids | Glycerophosphoserines |
| Pirimiphos-methyl | 72211 | Unclassified | Unclassified | Unclassified |
| Marimastat | HMDB0014924 | Organic oxygen compounds | Organooxygen compounds | Alcohols and polyols |
| DIHYDROCELASTROL | 44189 | Unclassified | Unclassified | Unclassified |
| Licoagrodin | HMDB0035372 | Phenylpropanoids and polyketides | Flavonoids | Flavans |
| Cerulenin | HMDB0015168 | Organoheterocyclic compounds | Epoxides | Oxirane carboxylic acids and derivatives |
| 4-Butyl-5-propylthiazole | 94608 | Organoheterocyclic compounds | Azoles | Thiazoles |
| PA(0:0/18:0) | LMGP10050043 | Lipids and lipid-like molecules | Glycerophospholipids | Glycerophosphates |
| (1S,2R,4R)-p-Menth-8-ene-2,10-diol 2-glucoside | HMDB0039055 | Lipids and lipid-like molecules | Prenol lipids | Terpene glycosides |
| Alanyl-Leucine | HMDB0028691 | Organic acids and derivatives | Carboxylic acids and derivatives | Amino acids, peptides, and analogues |
| 9,10-epoxyoctadecanoic acid | HMDB0061650 | Lipids and lipid-like molecules | Fatty Acyls | Fatty acids and conjugates |
| (Z)-3-Oxo-2-(2-pentenyl)-1-cyclopenteneacetic acid | HMDB0030197 | Organic oxygen compounds | Organooxygen compounds | Carbonyl compounds |
| Ricinine | HMDB0042006 | Organoheterocyclic compounds | Pyridines and derivatives | 3-pyridinecarbonitriles |
| PtdIns-(1,2-dihexanoyl) | 64898 | Unclassified | Unclassified | Unclassified |
| Endothion | 72741 | Unclassified | Unclassified | Unclassified |
| 4'-Methoxychalcone | LMPK12120188 | Phenylpropanoids and polyketides | Linear 1,3-diarylpropanoids | Chalcones and dihydrochalcones |
| 2-Hydroxy-2-ethylsuccinic acid | HMDB0059758 | Lipids and lipid-like molecules | Fatty Acyls | Fatty acids and conjugates |
| (S)-Nerolidol 3-O-[a-L-Rhamnopyranosyl-(1->4)-a-L-rhamnopyranosyl-(1->2)-b-D-glucopyranoside] | HMDB0040845 | Organic oxygen compounds | Organooxygen compounds | Carbohydrates and carbohydrate conjugates |
| Coriandrone C | HMDB0041525 | Phenylpropanoids and polyketides | Isocoumarins and derivatives | Unclassified |
| Crosatoside B | HMDB0039253 | Organic oxygen compounds | Organooxygen compounds | Carbohydrates and carbohydrate conjugates |
| 3-hydroxy-3-(3-hydroxyphenyl)propanoic acid-O-sulphate | HMDB0059967 | Organic acids and derivatives | Hydroxy acids and derivatives | Beta hydroxy acids and derivatives |
| 1-(5-Phosphoribosyl)-4-(N-succinocarboxamide)-5-aminoimidazole | 66260 | Unclassified | Unclassified | Unclassified |
| Promazine N-oxide sulfoxide | 2134 | Unclassified | Unclassified | Unclassified |
| 3,3'-Thiobispropanoic acid | HMDB0031162 | Organic acids and derivatives | Carboxylic acids and derivatives | Dicarboxylic acids and derivatives |
| Valyl-Glutamine | HMDB0029125 | Organic acids and derivatives | Carboxylic acids and derivatives | Amino acids, peptides, and analogues |
| PS(22:4(7Z,10Z,13Z,16Z)/22:5(4Z,7Z,10Z,13Z,16Z)) | HMDB0112811 | Lipids and lipid-like molecules | Glycerophospholipids | Glycerophosphoserines |
| N-Acetylhistidine | HMDB0032055 | Organic acids and derivatives | Carboxylic acids and derivatives | Amino acids, peptides, and analogues |
| Compactin diol lactone | 69858 | Unclassified | Unclassified | Unclassified |
| PG(16:0/0:0) | LMGP04050008 | Lipids and lipid-like molecules | Glycerophospholipids | Glycerophosphoglycerols |
| 5alpha-Androstan-3alpha,17beta-diol disulfate | HMDB0094682 | Lipids and lipid-like molecules | Steroids and steroid derivatives | Sulfated steroids |
| Agomelatine | HMDB0015636 | Organic acids and derivatives | Carboxylic acids and derivatives | Carboxylic acid derivatives |
| N-linoleoyl dopamine | LMFA08020270 | Lipids and lipid-like molecules | Fatty Acyls | Fatty amides |
| Methysergide | HMDB0014392 | Alkaloids and derivatives | Ergoline and derivatives | Lysergic acids and derivatives |
| 9-hydroxy-16-oxo-hexadecanoic acid | LMFA01170059 | Lipids and lipid-like molecules | Fatty Acyls | Fatty Acids and Conjugates |
| (±)-3-(4-Hydroxyphenyl)-1,2-propanediol 4'-O-glucoside | 88974 | Organic oxygen compounds | Organooxygen compounds | Carbohydrates and carbohydrate conjugates |
| Calpeptin | 68942 | Unclassified | Unclassified | Unclassified |
| UNC0224 | 64826 | Unclassified | Unclassified | Unclassified |
| Pergolide | HMDB0015317 | Organoheterocyclic compounds | Quinolines and derivatives | Indoloquinolines |
| Tyrosyl-Leucine | HMDB0029109 | Organic acids and derivatives | Carboxylic acids and derivatives | Amino acids, peptides, and analogues |
| L-Tryptophan | HMDB0000929 | Organoheterocyclic compounds | Indoles and derivatives | Indolyl carboxylic acids and derivatives |
| 17-HOME(9Z) | LMFA02000180 | Lipids and lipid-like molecules | Fatty Acyls | Octadecanoids |
| 2-Methylbutyroylcarnitine | HMDB0000378 | Lipids and lipid-like molecules | Fatty Acyls | Fatty acid esters |
| 4-ene-Valproic acid | HMDB0013897 | Lipids and lipid-like molecules | Fatty Acyls | Fatty acids and conjugates |
| 4-hydroxy-5-[4-hydroxy-3-(sulfooxy)phenyl]pentanoic acid | HMDB0127746 | Lipids and lipid-like molecules | Fatty Acyls | Fatty acids and conjugates |
| N-HFG | 64631 | Unclassified | Unclassified | Unclassified |
| 3,4,5-trihydroxy-6-[(4-hydroxy-3-methylbut-2-enoyl)oxy]oxane-2-carboxylic acid | HMDB0128929 | Organic oxygen compounds | Organooxygen compounds | Carbohydrates and carbohydrate conjugates |
| (+)-Myristinin A | LMPK12020229 | Lipids and lipid-like molecules | Polyketides | Flavonoids |
| 13(S)-HpOTrE | 36052 | Unclassified | Unclassified | Unclassified |
| S-Phenylmercapturic acid | HMDB0042011 | Organic acids and derivatives | Carboxylic acids and derivatives | Amino acids, peptides, and analogues |
| Alkaloid AQC2 | HMDB0038595 | Alkaloids and derivatives | Tropane alkaloids | Unclassified |
| Indicine | 68248 | Unclassified | Unclassified | Unclassified |
| 12-Hydroxy-7-oxo-8,11,13-abietatrien-18-al | HMDB0040746 | Lipids and lipid-like molecules | Prenol lipids | Diterpenoids |
| Aspartylphenylalanine | HMDB0000706 | Organic acids and derivatives | Carboxylic acids and derivatives | Amino acids, peptides, and analogues |
| (-)-11-Hydroxy-9,15,16-trioxooctadecanoic acid | HMDB0038940 | Lipids and lipid-like molecules | Fatty Acyls | Fatty acids and conjugates |
| Kanzonol J | HMDB0040605 | Phenylpropanoids and polyketides | Isoflavonoids | Pyranoisoflavonoids |
| Cucumopine | 67006 | Unclassified | Unclassified | Unclassified |
| xi-2,3-Dihydro-2-oxo-1H-indole-3-acetic acid | HMDB0035514 | Organoheterocyclic compounds | Indoles and derivatives | Indolyl carboxylic acids and derivatives |
| Nelumboside | HMDB0038464 | Phenylpropanoids and polyketides | Flavonoids | Flavonoid glycosides |
| L-cis-Cyclo(aspartylphenylalanyl) | HMDB0031360 | Organic acids and derivatives | Carboxylic acids and derivatives | Amino acids, peptides, and analogues |
| O-Ureidohomoserine | HMDB0012271 | Organic acids and derivatives | Carboxylic acids and derivatives | Amino acids, peptides, and analogues |
| Aspartyl-Isoleucine | HMDB0028756 | Organic acids and derivatives | Carboxylic acids and derivatives | Amino acids, peptides, and analogues |
| 4-hydroxy-8cis-sphingenine | LMSP01080009 | Lipids and lipid-like molecules | Sphingolipids | Sphingoid bases |
| 1-(9Z,12Z,15Z-octadecatrienoyl)-glycero-3-phosphate | HMDB0062320 | Lipids and lipid-like molecules | Glycerophospholipids | Glycerophosphates |
| Prosulfocarb | 72532 | Unclassified | Unclassified | Unclassified |
| 16-hydroxy-9E-hexadecenoic acid | LMFA01050192 | Lipids and lipid-like molecules | Fatty Acyls | Fatty Acids and Conjugates |
| Gymnodimine | 73510 | Organoheterocyclic compounds | Pyridines and derivatives | Hydropyridines |
| Vitamin D2 3-glucuronide | LMST03010072 | Lipids and lipid-like molecules | Steroids and steroid derivatives | Vitamin D and derivatives |
| PC(14:2(Z)l8,8/0:0)[U] | 40281 | Unclassified | Unclassified | Unclassified |
| MG(18:0/0:0/0:0) | 61993 | Lipids and lipid-like molecules | Glycerolipids | Monoradylglycerols |
| 6beta-acetoxy-24-methylcholestan-3beta,5alpha,22R,24-tetrol | 84015 | Unclassified | Unclassified | Unclassified |
| Doxorubicin-semiquinone | HMDB0060822 | Organic oxygen compounds | Organooxygen compounds | Carbohydrates and carbohydrate conjugates |
| Mannosyl-1beta-phosphomycoketide C30 | LMPK01000058 | Lipids and lipid-like molecules | Polyketides | Linear polyketides |
| Aprepitant | HMDB0014811 | Organoheterocyclic compounds | Oxazinanes | Morpholines |
| Caryoptosidic acid | HMDB0034249 | Lipids and lipid-like molecules | Prenol lipids | Terpene glycosides |
| Pogostol | HMDB0033239 | Lipids and lipid-like molecules | Prenol lipids | Sesquiterpenoids |
| desmethylastemizole | HMDB0061013 | Organoheterocyclic compounds | Benzimidazoles | Unclassified |
| 9(R)-HODE | 45660 | Unclassified | Unclassified | Unclassified |
| Propofol glucuronide | HMDB0060933 | Organic oxygen compounds | Organooxygen compounds | Carbohydrates and carbohydrate conjugates |
| (R)C(R)S-S-Propylcysteine sulfoxide | HMDB0029442 | Organic acids and derivatives | Carboxylic acids and derivatives | Amino acids, peptides, and analogues |
| Binapacryl | 72773 | Unclassified | Unclassified | Unclassified |
| 5-ethyl-5-methyl-2,4-oxazolidinedione | HMDB0061082 | Organoheterocyclic compounds | Azolidines | Oxazolidines |
| Westiellamide | 71070 | Unclassified | Unclassified | Unclassified |
| 1H-Indole-3-carboxaldehyde | HMDB0029737 | Organoheterocyclic compounds | Indoles and derivatives | Indoles |
| 9R,10S-Epoxy-3Z,6Z-nonadecadiene | LMFA12000294 | Lipids and lipid-like molecules | Fatty Acyls | Oxygenated hydrocarbons |
| DG(18:2(9Z,12Z)/22:4(7Z,10Z,13Z,16Z)/0:0) | HMDB0007263 | Lipids and lipid-like molecules | Glycerolipids | Diradylglycerols |
| DIPROTIN A | 44309 | Unclassified | Unclassified | Unclassified |
| [6]-Gingerdiol 5-O-beta-D-glucopyranoside | HMDB0036123 | Lipids and lipid-like molecules | Fatty Acyls | Fatty acyl glycosides |
| 6-Oxopiperidine-2-carboxylic acid | HMDB0061705 | Organic acids and derivatives | Carboxylic acids and derivatives | Amino acids, peptides, and analogues |
| PA(P-16:0/0:0) | LMGP10070003 | Lipids and lipid-like molecules | Glycerophospholipids | Glycerophosphates |
| Tartronate semialdehyde | HMDB0006938 | Organic oxygen compounds | Organooxygen compounds | Carbohydrates and carbohydrate conjugates |
| Ikarisoside D | LMPK12111724 | Lipids and lipid-like molecules | Polyketides | Flavonoids |
| (±)-threo-1-(p-Hydroxyphenyl)propylene glycol 4'-glucoside | HMDB0033068 | Organic oxygen compounds | Organooxygen compounds | Carbohydrates and carbohydrate conjugates |
| Gibberellin A36 | LMPR0104170019 | Lipids and lipid-like molecules | Prenol lipids | Isoprenoids |
| Succinic acid | HMDB0000254 | Organic acids and derivatives | Carboxylic acids and derivatives | Dicarboxylic acids and derivatives |
| METHOPRENE (S) | 44012 | Unclassified | Unclassified | Unclassified |
| 3',4'-Methylenedioxy-[2'',3'':7,8]furanoflavanone | LMPK12140068 | Unclassified | Unclassified | Unclassified |
| 2-Naphthalenol 2-aminobenzoate | 94419 | Benzenoids | Naphthalenes | Unclassified |
| 3,6-octadecadiynoic acid | LMFA01030515 | Lipids and lipid-like molecules | Fatty Acyls | Fatty Acids and Conjugates |
| L-Agaridoxin | HMDB0029445 | Organic acids and derivatives | Carboxylic acids and derivatives | Amino acids, peptides, and analogues |
| PC(O-6:0/O-6:0) | 40250 | Unclassified | Unclassified | Unclassified |
| Chamanetin | 52636 | Lipids and lipid-like molecules | Polyketides | Flavonoids |
| L-Oxalylalbizziine | HMDB0039164 | Organic acids and derivatives | Carboxylic acids and derivatives | Amino acids, peptides, and analogues |
| 1-Pentanesulfenothioic acid | HMDB0031160 | Organosulfur compounds | Sulfenyl compounds | Unclassified |
| Glutaminylleucine | HMDB0028801 | Organic acids and derivatives | Carboxylic acids and derivatives | Amino acids, peptides, and analogues |
| 15-OxoEDE | 45658 | Unclassified | Unclassified | Unclassified |
| Pentostatin | HMDB0014692 | Organoheterocyclic compounds | Imidazodiazepines | Unclassified |
| Austroinulin | HMDB0036802 | Lipids and lipid-like molecules | Prenol lipids | Diterpenoids |
| Isoleucyl-Arginine | HMDB0028901 | Organic acids and derivatives | Carboxylic acids and derivatives | Amino acids, peptides, and analogues |
| Tetranor-12(R)-HETE | 35513 | Unclassified | Unclassified | Unclassified |
| 6-Caffeoylsucrose | HMDB0035486 | Lipids and lipid-like molecules | Steroids and steroid derivatives | Steroidal glycosides |
| Isoleucyl-Lysine | HMDB0028912 | Organic acids and derivatives | Carboxylic acids and derivatives | Amino acids, peptides, and analogues |
| Boc-Asn-OPhNO2 | 65720 | Unclassified | Unclassified | Unclassified |
| Wasalexin A | HMDB0034765 | Organoheterocyclic compounds | Indoles and derivatives | Unclassified |
| JWH 307 | 85204 | Unclassified | Unclassified | Unclassified |
| Phenethyl decanoate | HMDB0032464 | Lipids and lipid-like molecules | Fatty Acyls | Fatty acid esters |
| 3-(hydroxymethyl)-1H-isochromen-1-one | HMDB0128615 | Phenylpropanoids and polyketides | Isocoumarins and derivatives | Unclassified |
| Physagulin A | HMDB0041047 | Lipids and lipid-like molecules | Steroids and steroid derivatives | Steroid lactones |
| 13S-HpODE | LMFA02000034 | Lipids and lipid-like molecules | Fatty Acyls | Octadecanoids |
| (-)-Blebbistatin | 45431 | Unclassified | Unclassified | Unclassified |
| 2-Keto-3-deoxyoctonate (KDO) | 4143 | Unclassified | Unclassified | Unclassified |
| N-Succinyl-L-citrulline | 63487 | Unclassified | Unclassified | Unclassified |
| Petunidin 3-(6''-acetylglucoside) | LMPK12010356 | Unclassified | Unclassified | Unclassified |
| L-Leucine | HMDB0000687 | Organic acids and derivatives | Carboxylic acids and derivatives | Amino acids, peptides, and analogues |
| Fludarabine phosphate | 675 | Nucleosides, nucleotides, and analogues | Purine nucleotides | Purine ribonucleotides |
| 8-hydroxy-13E,17-Octadecadiene-9,11-diynoic acid | LMFA01031064 | Lipids and lipid-like molecules | Fatty Acyls | Fatty Acids and Conjugates |
| Hex-Honaucin A | 65488 | Unclassified | Unclassified | Unclassified |
| Morphine 3,6-diglucuronide | 1360 | Unclassified | Unclassified | Unclassified |
| Pseudomonine | HMDB0041438 | Organic acids and derivatives | Carboxylic acids and derivatives | Amino acids, peptides, and analogues |
| trans-Ferulic acid | HMDB0000954 | Phenylpropanoids and polyketides | Cinnamic acids and derivatives | Hydroxycinnamic acids and derivatives |
| Pipecuronium | HMDB0015431 | Lipids and lipid-like molecules | Steroids and steroid derivatives | Steroid esters |
| 1-Deoxy-D-xylulose | HMDB0001292 | Organic oxygen compounds | Organooxygen compounds | Carbohydrates and carbohydrate conjugates |
| monodeallydihydroxyalmitrine | 927 | Unclassified | Unclassified | Unclassified |
| 6-({3,7-dihydroxy-2-[3-hydroxy-10-(4-hydroxy-3-methoxyphenyl)-2-oxo-4-oxatricyclo[4.3.1.0³,⁷]decan-8-yl]-4-oxo-3,4-dihydro-2H-1-benzopyran-5-yl}oxy)-3,4,5-trihydroxyoxane-2-carboxylic acid | HMDB0128741 | Phenylpropanoids and polyketides | Flavonoids | Flavans |
| 5,2',6'-Trihydroxy-7,8-dimethoxyflavone 2'-glucuronide | LMPK12111296 | Unclassified | Unclassified | Unclassified |
| Aminoadipic acid | HMDB0000510 | Organic acids and derivatives | Carboxylic acids and derivatives | Amino acids, peptides, and analogues |
| Carphenazine | HMDB0015172 | Organoheterocyclic compounds | Benzothiazines | Phenothiazines |
| Acrinathrin | 72216 | Unclassified | Unclassified | Unclassified |
| 6-Chloro-N-(1-methylethyl)-1,3,5-triazine-2,4-diamine | HMDB0033249 | Organoheterocyclic compounds | Triazines | Aminotriazines |
| 5,6,7-trihydroxy-3-phenyl-4H-chromen-4-one | HMDB0129963 | Phenylpropanoids and polyketides | Isoflavonoids | Isoflav-2-enes |
| 1-(2-Thienyl)-1,2-propanedione | HMDB0040002 | Organic oxygen compounds | Organooxygen compounds | Carbonyl compounds |
| Kabiramide C | 65484 | Unclassified | Unclassified | Unclassified |
| Lysyl-Serine | 85862 | Organic acids and derivatives | Carboxylic acids and derivatives | Amino acids, peptides, and analogues |
| Diphemanil Methylsulfate | 85422 | Unclassified | Unclassified | Unclassified |
| 3-O-(6-O-alpha-D-Xylosylphospho-alpha-D-mannopyranosyl)-alpha-D-mannopyranose | 73349 | Unclassified | Unclassified | Unclassified |
| Astin I | HMDB0041419 | Organic acids and derivatives | Peptidomimetics | Hybrid peptides |
| N-Phenylacetylglutamic acid | HMDB0059772 | Organic acids and derivatives | Carboxylic acids and derivatives | Amino acids, peptides, and analogues |
| Lyso-PAF C-16 | 24070 | Unclassified | Unclassified | Unclassified |
| {4-[5,7-dihydroxy-8-(3-methylbut-2-en-1-yl)-4-oxo-3,4-dihydro-2H-1-benzopyran-3-yl]-2-hydroxy-6-(3-methylbut-2-en-1-yl)phenyl}oxidanesulfonic acid | HMDB0134748 | Phenylpropanoids and polyketides | Isoflavonoids | Isoflavans |
| N5-(4-Methoxybenzyl)glutamine | HMDB0033598 | Organic acids and derivatives | Carboxylic acids and derivatives | Amino acids, peptides, and analogues |
| PG(a-13:0/a-15:0) | HMDB0116638 | Lipids and lipid-like molecules | Glycerophospholipids | Glycerophosphoglycerols |
| Trigonelline | HMDB0000875 | Alkaloids and derivatives | Unclassified | Unclassified |
| Ketotifen | HMDB0015056 | Organoheterocyclic compounds | Cycloheptathiophenes | Unclassified |

Table S6 The positive ion modes of metabolites between T1 and T2

| **ID** | **Metabolites** | **Formula** | **m/z** | **Retention time (min)** | **Class** | **P-value** | **VIP** |
| --- | --- | --- | --- | --- | --- | --- | --- |
| 4.68_149.0238m/z | Phthalic acid | C8H6O4 | 149.024 | 4.681816667 | Benzene and substituted derivatives | 1.7E-07 | 52.1295 |
| 11.41_294.2198n | γ- 6(7)-EpODE | C18H30O3 | 277.216 | 11.40518333 | Unclassified | 0.0062 | 16.3901 |
| 2.02_208.0939n | Dambonitol | C8H16O6 | 231.084 | 2.019183333 | Organooxygen compounds | 1.4E-07 | 11.5555 |
| 13.23_122.0961m/z | Tropinone | C8H13NO | 122.096 | 13.22638333 | Unclassified | 0.03534 | 11.2242 |
| 4.68_514.0440n | (2-{[2-(3,4-dihydroxyphenyl)-5,7-dihydroxy-4-oxo-4H-chromen-3-yl]oxy}-4-hydroxy-5-(hydroxymethyl)oxolan-3-yl)oxidanesulfonic acid | C20H18O14S | 537.033 | 4.681816667 | Flavonoids | 1.4E-08 | 10.3459 |
| 5.16_386.1214n | 3,4,5-trihydroxy-6-{[4-hydroxy-5-(3-hydroxyphenyl)pentanoyl]oxy}oxane-2-carboxylic acid | C17H22O10 | 404.155 | 5.157133333 | Organooxygen compounds | 0.00031 | 10.3161 |
| 4.68_121.0276m/z | 3,4-Dihydroxybenzaldehyde | C7H6O3 | 121.028 | 4.681816667 | Organooxygen compounds | 2.7E-06 | 7.95318 |
| 2.02_309.1314n | Tyrosyl-Glutamine | C14H19N3O5 | 332.1232 | 2.019183333 | Carboxylic acids and derivatives | 8.83189E-07 | 6.599188 |
| 13.33_356.2925n | MG(18:1(9Z)/0:0/0:0) | C21H40O4 | 357.2998 | 13.3337 | Glycerolipids | 0.015727618 | 6.339056 |
| 12.00_352.2615n | MG(0:0/18:3(6Z,9Z,12Z)/0:0) | C21H36O4 | 353.2688 | 11.9983 | Fatty Acyls | 0.001982971 | 6.035293 |
| 0.80_324.1036n | Prazepam | C19H17ClN2O | 325.1124 | 0.804766667 | Benzodiazepines | 0.032588216 | 5.961976 |
| 2.53_103.0577m/z | Cyclopentanethiol | C5H10S | 103.0577 | 2.526816667 | Thiols | 0.044513486 | 5.467572 |
| 2.03_405.1911n | Imidapril | C20H27N3O6 | 444.1557 | 2.03435 | Carboxylic acids and derivatives | 4.67262E-06 | 5.372643 |
| 6.94_269.1047n | alpha-(p-Methoxyphenyl)-6-methyl-2-pyridineacrylic acid | C16H15NO3 | 270.112 | 6.943666667 | Unclassified | 7.58223E-05 | 5.107889 |
| 12.59_354.2764n | MG(0:0/18:2(9Z,12Z)/0:0) | C21H38O4 | 355.2836 | 12.59191667 | Fatty Acyls | 0.00387851 | 5.085925 |
| 0.76_220.0553n | 2-(2-Thienylmethylene)-1,6-dioxaspiro[4.4]non-3-ene | C12H12O2S | 203.052 | 0.760783333 | Organooxygen compounds | 0.001263763 | 4.979657 |
| 15.84_124.0867m/z | L-Histidinol | C6H11N3O | 124.0867 | 15.84201667 | Organonitrogen compounds | 0.032568159 | 4.972221 |
| 2.16_132.1016m/z | L-Isoleucine | C6H13NO2 | 132.1016 | 2.1555 | Carboxylic acids and derivatives | 0.003092788 | 4.831405 |
| 2.02_325.1124m/z | Inulobiose | C12H22O11 | 325.1124 | 2.019183333 | Organooxygen compounds | 4.44714E-05 | 4.735675 |
| 2.02_228.0754m/z | Homocitrulline | C7H15N3O3 | 228.0754 | 2.019183333 | Carboxylic acids and derivatives | 2.93315E-10 | 4.705866 |
| 12.52_278.2243n | Gamma-Linolenic acid | C18H30O2 | 279.2316 | 12.51576667 | Fatty Acyls | 0.005721276 | 4.436461 |
| 15.02_936.5808n | 1,2-Di-(9Z,12Z,15Z-octadecatrienoyl)-3-(Galactosyl-alpha-1-6-Galactosyl-beta-1)-glycerol | C51H84O15 | 954.6147 | 15.02341667 | Glycerolipids | 0.018863941 | 4.215574 |
| 6.50_305.0685m/z | Taxifolin | C15H12O7 | 305.0685 | 6.498866667 | Flavonoids | 0.000146068 | 4.15944 |
| 0.85_360.1492m/z | beta-Lactose | C12H22O11 | 360.1492 | 0.849933333 | Organooxygen compounds | 4.34296E-05 | 4.139474 |
| 12.52_278.2243n | Gamma-Linolenic acid | C18H30O2 | 279.2316 | 12.51576667 | Fatty Acyls | 0.005721276 | 4.436461 |
| 15.02_936.5808n | 1,2-Di-(9Z,12Z,15Z-octadecatrienoyl)-3-(Galactosyl-alpha-1-6-Galactosyl-beta-1)-glycerol | C51H84O15 | 954.6147 | 15.02341667 | Glycerolipids | 0.018863941 | 4.215574 |
| 6.50_305.0685m/z | Taxifolin | C15H12O7 | 305.0685 | 6.498866667 | Flavonoids | 0.000146068 | 4.15944 |
| 0.85_360.1492m/z | beta-Lactose | C12H22O11 | 360.1492 | 0.849933333 | Organooxygen compounds | 4.34296E-05 | 4.139474 |
| 3.83_224.1280m/z | 2,3-Dihydro-6-methoxy-2,2-dimethyl-4H-1-benzopyran-4-one | C12H14O3 | 224.128 | 3.830883333 | Benzopyrans | 0.045520901 | 4.041629 |
| 15.94_586.4625n | DG(16:1n7/0:0/18:4n3) | C37H62O5 | 587.4698 | 15.94451667 | Fatty Acyls | 0.008252355 | 4.00564 |
| 4.94_386.1207n | 6-[(4,7-dihydroxy-2,2-dimethyl-3,4-dihydro-2H-1-benzopyran-5-yl)oxy]-3,4,5-trihydroxyoxane-2-carboxylic acid | C17H22O10 | 369.1174 | 4.937983333 | Organooxygen compounds | 0.00028702 | 3.854879 |
| 2.02_418.1356n | 2-N,6-N-Bis(2,3-dihydroxybenzoyl)-L-lysine | C20H22N2O8 | 436.1695 | 2.019183333 | Unclassified | 5.5185E-07 | 3.851092 |
| 5.16_210.0885n | 2-Methoxy-3-(4-methoxyphenyl)propanoic acid | C11H14O4 | 193.0852 | 5.157133333 | Phenylpropanoic acids | 0.000263122 | 3.62139 |
| 12.59_262.2288n | Farnesyl acetone | C18H30O | 263.2365 | 12.59191667 | Unclassified | 0.002680415 | 3.556801 |
| 10.99_292.2033n | Colnelenic acid | C18H28O3 | 275.2 | 10.99403333 | Fatty Acyls | 0.015437546 | 3.523755 |
| 4.68_521.0535m/z | 6-[4-carboxy-2-hydroxy-6-(3,4,5-trihydroxybenzoyloxy)phenoxy]-3,4,5-trihydroxyoxane-2-carboxylic acid | C20H18O15 | 521.0535 | 4.681816667 | Tannins | 0.000607376 | 3.501551 |
| 1.48_130.0494m/z | (R)-(+)-2-Pyrrolidone-5-carboxylic acid | C5H7NO3 | 130.0494 | 1.47605 | Unclassified | 0.048640524 | 3.417025 |
| 10.89_222.1620n | R-cucujolide V | C14H22O2 | 223.1693 | 10.88855 | Fatty Acyls | 0.034608553 | 3.364842 |
| 9.00_308.1981n | 16-B1-PhytoP | C18H28O4 | 291.1948 | 9.00035 | Fatty Acyls | 0.006664761 | 3.313178 |
| 11.59_279.2310m/z | Alpha-Linolenic acid | C18H30O2 | 279.231 | 11.58566667 | Fatty Acyls | 0.013170352 | 3.179952 |
| 8.19_261.1097m/z | Benzyl b-L-arabinopyranoside | C13H18O4 | 261.1097 | 8.188733333 | Unclassified | 0.023851691 | 3.121406 |
| 2.02_468.1111n | {3,8,15-trihydroxy-16,17-dimethoxy-9-oxotricyclo[12.3.1.1²,⁶]nonadeca-1(17),2,4,6(19),14(18),15-hexaen-7-yl}oxidanesulfonic acid | C21H24O10S | 507.0899 | 2.019183333 | Diarylheptanoids | 3.91596E-07 | 3.011041 |
| 3.70_643.1156m/z | 6-[5-({[3,5-dihydroxy-2-(hydroxymethyl)-6-(3,4,5-trihydroxybenzoyloxy)oxan-4-yl]oxy}carbonyl)-2,3-dihydroxyphenoxy]-3,4,5-trihydroxyoxane-2-carboxylic acid | C26H28O20 | 643.1156 | 3.6974 | Tannins | 0.018406998 | 2.988685 |
| 4.78_226.0474n | 3-(3,4-dihydroxy-5-methoxyphenyl)oxirane-2-carboxylic acid | C10H10O6 | 209.0441 | 4.784316667 | Phenols | 0.008394447 | 2.970067 |
| 2.73_208.0364n | 3-(2H-1,3-benzodioxol-5-yl)-3-oxopropanoic acid | C10H8O5 | 226.0719 | 2.7303 | Benzodioxoles | 0.04132615 | 2.944605 |
| 2.02_306.0938n | Starch acetate | C12H18O9 | 324.1336 | 2.019183333 | Carboxylic acids and derivatives | 2.58912E-06 | 2.937154 |
| 4.63_263.1386m/z | Phenylalanylproline | C14H18N2O3 | 263.1386 | 4.634333333 | Carboxylic acids and derivatives | 0.008011674 | 2.804923 |
| 2.02_350.1096m/z | beta-Glucogallin | C13H16O10 | 350.1096 | 2.019183333 | Tannins | 2.30867E-09 | 2.754479 |
| 12.06_358.3668m/z | Behenic acid | C22H44O2 | 358.3668 | 12.0588 | Fatty Acyls | 0.013741626 | 2.64768 |
| 5.23_286.2008m/z | 3,11,12-Trihydroxy-1(10)-spirovetiven-2-one | C15H24O4 | 286.2008 | 5.23195 | Prenol lipids | 0.003844799 | 2.636094 |
| 15.34_731.5540n | Araliacerebroside | C40H77NO10 | 732.5613 | 15.3359 | Sphingolipids | 0.015411731 | 2.535967 |
| 0.88_152.0562m/z | Guanine | C5H5N5O | 152.0562 | 0.8811 | Imidazopyrimidines | 0.007138997 | 2.524471 |
| 13.91_283.2625m/z | 15-hydroxy stearic acid | C18H36O3 | 283.2625 | 13.91016667 | Fatty Acyls | 0.002249717 | 2.515591 |
| 13.33_264.2444n | 9,12,15-Octadecatrien-1-ol | C18H32O | 265.252 | 13.3337 | Fatty Acyls | 0.011778655 | 2.4925 |
| 8.73_211.1683m/z | 11-Hydroxy-9-tridecenoic acid | C13H24O3 | 211.1683 | 8.729533333 | Fatty Acyls | 0.022958994 | 2.487117 |
| 4.91_374.1357n | (-)-Wikstromol | C20H22O7 | 357.1324 | 4.908466667 | Furanoid lignans | 0.000156956 | 2.448205 |
| 2.02_245.0762m/z | Uridine | C9H12N2O6 | 245.0762 | 2.019183333 | Pyrimidine nucleosides | 0.005791341 | 2.428458 |
| 2.03_495.1481n | Sodium folinate | C20H22N7NaO7 | 518.1373 | 2.03435 | Unclassified | 6.25883E-07 | 2.411136 |
| 0.93_118.0857m/z | L-Valine | C5H11NO2 | 118.0857 | 0.926933333 | Carboxylic acids and derivatives | 0.00406525 | 2.38256 |
| 2.17_502.2484m/z | Bilastine | C28H37N3O3 | 502.2484 | 2.170683333 | Benzimidazoles | 0.034742193 | 2.357054 |
| 4.49_294.1221n | Aspartame | C14H18N2O5 | 317.1207 | 4.494333333 | Carboxylic acids and derivatives | 0.001460523 | 2.328013 |
| 2.08_367.0677m/z | 6-{[3-(2H-1,3-benzodioxol-5-yl)-3-oxopropanoyl]oxy}-3,4,5-trihydroxyoxane-2-carboxylic acid | C16H16O11 | 367.0677 | 2.079683333 | Organooxygen compounds | 1.76735E-05 | 2.321428 |
| 5.71_163.0380m/z | Umbelliferone | C9H6O3 | 163.038 | 5.710416667 | Coumarins and derivatives | 0.000137237 | 2.287568 |
| 0.78_383.1137m/z | 5-Hydroxyflavone | C21H20O8 | 383.1137 | 0.775783333 | Flavonoids | 0.001816602 | 2.285302 |
| 8.06_238.1201n | 1,2,3,4-Tetramethoxy-5-(2-propenyl)benzene | C13H18O4 | 261.1093 | 8.05575 | Phenol ethers | 0.0390577 | 2.264807 |
| 13.15_262.2288n | 9,12,15-octadecatrienal | C18H30O | 263.2359 | 13.15105 | Fatty Acyls | 0.001047991 | 2.253135 |
| 4.92_395.2331m/z | 11-dehydro-TXB2-d4 | C20H28D4O6 | 395.2331 | 4.922983333 | Unclassified | 0.014980491 | 2.252527 |
| 0.83_684.2298n | Galabiose | C24H44O22 | 685.2411 | 0.834766667 | Organooxygen compounds | 0.000353713 | 2.251822 |
| 11.65_278.1511n | Monoethylhexyl phthalic acid | C16H22O4 | 279.1584 | 11.647 | Benzene and substituted derivatives | 0.032504551 | 2.244576 |
| 2.02_358.0959m/z | Azidocillin | C16H17N5O4S | 358.0959 | 2.019183333 | Lactams | 1.84285E-08 | 2.244198 |
| 5.14_265.0942n | 2-(2-Phenylacetoxy)propionylglycine | C13H15NO5 | 266.1015 | 5.1413 | Carboxylic acids and derivatives | 0.015423275 | 2.236966 |
| 6.50_307.0654m/z | Xanthosine | C10H12N4O6 | 307.0654 | 6.498866667 | Purine nucleosides | 0.000103129 | 2.229727 |
| 3.95_443.2375m/z | O-Desmethyltramadol glucuronide | C21H31NO8 | 443.2375 | 3.951216667 | Organooxygen compounds | 0.018648901 | 2.194365 |
| 0.87_227.0424n | 5-(2'-Carboxyethyl)-4,6-Dihydroxypicolinate | C9H9NO6 | 228.0497 | 0.865266667 | Pyridines and derivatives | 0.025661234 | 2.184372 |
| 13.91_264.2444n | (±)-(Z)-2-(5-Tetradecenyl)cyclobutanone | C18H32O | 265.2518 | 13.91016667 | Organooxygen compounds | 0.002404045 | 2.179143 |
| 4.01_460.2181m/z | Lusitanicoside | C21H30O10 | 460.2181 | 4.0102 | Organooxygen compounds | 0.021148203 | 2.173256 |
| 5.82_408.2585m/z | (3S,5R,6S,7E,9x)-7-Megastigmene-3,6,9-triol 9-glucoside | C19H34O8 | 408.2585 | 5.818083333 | Fatty Acyls | 0.03415835 | 2.170323 |
| 8.84_278.0859n | (2-Chlorophenyl)diphenylmethane | C19H15Cl | 279.0932 | 8.837783333 | Unclassified | 0.019174842 | 2.154827 |
| 14.84_386.3409m/z | N-(16,16-dimethy-5Z,8Z,11Z,14Z-docosatetraenoyl)-ethanolamine | C26H45NO2 | 386.3409 | 14.83726667 | Fatty Acyls | 0.017582829 | 2.139502 |
| 2.02_113.0340m/z | Uracil | C4H4N2O2 | 113.034 | 2.019183333 | Diazines | 0.027894638 | 2.129124 |
| 8.88_318.3002m/z | 17-hydroxy stearic acid | C18H36O3 | 318.3002 | 8.882016667 | Fatty Acyls | 0.01829888 | 2.107985 |
| 12.23_582.4333n | Polidocanol | C30H62O10 | 600.4671 | 12.22695 | Unclassified | 0.038780114 | 2.079196 |
| 3.29_294.0944n | 1-O-(3R,4-dihydroxy-2-methylene-butanoyl)-beta-D-glucopyranose | C11H18O9 | 312.1283 | 3.286583333 | Fatty Acyls | 0.008148406 | 1.983559 |
| 4.68_523.0473m/z | 1,6-Digalloyl-beta-D-glucopyranose | C20H20O14 | 523.0473 | 4.681816667 | Tannins | 1.72812E-06 | 1.982639 |
| 0.88_504.1680n | Maltotriose | C18H32O16 | 527.1556 | 0.8811 | Organooxygen compounds | 0.017314134 | 1.978814 |
| 5.16_772.2403n | Alhagidin | C34H44O20 | 795.2298 | 5.157133333 | Polyketides | 0.000837634 | 1.947688 |
| 3.83_165.0540m/z | 9-hydroxy-7E-Nonene-3,5-diynoic acid | C9H8O3 | 165.054 | 3.830883333 | Fatty Acyls | 0.045714475 | 1.945059 |
| 13.11_410.3044n | 15(S)-15-methyl PGF2α isopropyl ester | C24H42O5 | 411.3117 | 13.10538333 | Unclassified | 0.016541781 | 1.939454 |
| 0.76_432.1697m/z | beta-D-Xylopyranosyl-(1->5)-alpha-L-arabinofuranosyl-(1->5)-L-arabinose | C15H26O13 | 432.1697 | 0.760783333 | Organooxygen compounds | 0.000463019 | 1.890836 |
| 2.47_307.0463m/z | 6-Thioinosinic acid | C10H12N4O4S | 307.0463 | 2.46815 | Purine nucleosides | 1.5831E-05 | 1.889866 |
| 2.08_260.1612m/z | Isoleucyl-Glutamine | C11H21N3O4 | 260.1612 | 2.079683333 | Carboxylic acids and derivatives | 0.020398216 | 1.874067 |
| 13.15_281.2467m/z | 17-HOME(9Z) | C18H34O3 | 281.2467 | 13.15105 | Fatty Acyls | 0.001480008 | 1.845843 |
| 12.30_328.2605n | Nonadecandioic acid | C19H36O4 | 329.2678 | 12.30278333 | Fatty Acyls | 0.007899658 | 1.842804 |
| 2.02_247.0566m/z | 2-benzyl-2-hydroxybutanedioic acid | C11H12O5 | 247.0566 | 2.019183333 | Phenylpropanoic acids | 1.95815E-09 | 1.8351 |
| 0.87_174.0395m/z | gamma-Carboxyglutamic acid | C6H9NO6 | 174.0395 | 0.865266667 | Carboxylic acids and derivatives | 0.020628516 | 1.811781 |
| 4.91_536.1884n | 8-Hydroxypinoresinol 8-glucoside | C26H32O12 | 559.1776 | 4.908466667 | Lignan glycosides | 0.000383559 | 1.794577 |
| 12.00_260.2131n | 6-[1]-ladderane hexanol | C18H28O | 261.2206 | 11.9983 | Fatty Acyls | 0.001274812 | 1.768316 |
| 3.76_428.1575m/z | Cilazprilat | C20H27N3O5 | 428.1575 | 3.756716667 | Unclassified | 0.048116033 | 1.743171 |
| 2.14_247.1281m/z | Glutamylvaline | C10H18N2O5 | 247.1281 | 2.140333333 | Carboxylic acids and derivatives | 0.010219213 | 1.728759 |
| 7.72_219.1484m/z | N-(4-aminobutyl)-3-(4-hydroxyphenyl)propanimidic acid | C13H20N2O2 | 219.1484 | 7.715766667 | Phenols | 0.01993406 | 1.706937 |
| 4.67_355.0318m/z | 4-(N-Maleimido)benzyltrimethylammonium iodide | C14H17IN2O2 | 355.0318 | 4.66565 | Unclassified | 0.001754533 | 1.695669 |
| 5.16_425.0831m/z | 6-[(4,5-dihydroxy-2,2-dimethyl-3,4-dihydro-2H-1-benzopyran-7-yl)oxy]-3,4,5-trihydroxyoxane-2-carboxylic acid | C17H22O10 | 425.0831 | 5.157133333 | Organooxygen compounds | 0.000776395 | 1.68059 |
| 2.03_339.1106m/z | Bifenazate | C17H20N2O3 | 339.1106 | 2.03435 | Unclassified | 2.74167E-07 | 1.65289 |
| 0.82_343.1224m/z | Melibiose | C12H22O11 | 343.1224 | 0.819766667 | Organooxygen compounds | 0.002083749 | 1.611712 |
| 14.00_679.2443n | 4-({2-[(3-{2-[2-(acetyloxy)propan-2-yl]-7-oxo-2H,3H,7H-furo[3,2-g]chromen-6-yl}-1-hydroxy-3-methylbutan-2-yl)sulfanyl]-1-[(carboxymethyl)-C-hydroxycarbonimidoyl]ethyl}-C-hydroxycarbonimidoyl)-2-aminobutanoic acid | C31H41N3O12S | 680.2516 | 14.00165 | Carboxylic acids and derivatives | 0.039974194 | 1.592922 |
| 4.85_331.1380m/z | (±)-threo-1-(p-Hydroxyphenyl)propylene glycol 4'-glucoside | C15H22O8 | 331.138 | 4.846483333 | Organooxygen compounds | 0.003681541 | 1.585634 |
| 1.97_378.0595n | 3,4,5-trihydroxy-6-({7-oxo-7H-furo[3,2-g]chromen-4-yl}oxy)oxane-2-carboxylic acid | C17H14O10 | 417.0212 | 1.97335 | Organooxygen compounds | 0.000223586 | 1.560268 |
| 2.19_416.2114m/z | famprofazone | C24H31N3O | 416.2114 | 2.185833333 | Azoles | 6.55601E-05 | 1.55272 |
| 5.02_312.1194n | 3-Hydroxychavicol 1-glucoside | C15H20O7 | 313.1268 | 5.015966667 | Organooxygen compounds | 0.001944561 | 1.547503 |
| 2.57_254.1603m/z | Arginyl-Proline | C11H21N5O3 | 254.1603 | 2.569816667 | Carboxylic acids and derivatives | 0.045044585 | 1.539064 |
| 0.82_127.0379m/z | 5-Hydroxymethyl-2-furancarboxaldehyde | C6H6O3 | 127.0379 | 0.819766667 | Organooxygen compounds | 0.032942831 | 1.535743 |
| 0.85_138.0542m/z | Trigonelline | C7H7NO2 | 138.0542 | 0.849933333 | Unclassified | 0.00016103 | 1.504395 |
| 8.06_336.0792m/z | SB 221284 | C16H14F3N3OS | 336.0792 | 8.05575 | Unclassified | 0.016986867 | 1.491748 |
| 13.93_380.2083m/z | Gibberellin A36 | C20H26O6 | 380.2083 | 13.9255 | Prenol lipids | 0.001291621 | 1.488069 |
| 0.78_119.0571n | L-Threonine | C4H9NO3 | 120.0641 | 0.775783333 | Carboxylic acids and derivatives | 0.007275153 | 1.488013 |
| 7.95_305.1276m/z | Ketophenylbutazone | C19H18N2O3 | 305.1276 | 7.952916667 | Benzene and substituted derivatives | 0.0021746 | 1.487536 |
| 4.72_458.1338n | 5a,11a-Dehydrooxytetracycline | C22H22N2O9 | 476.1676 | 4.724483333 | Unclassified | 0.001921498 | 1.461902 |
| 2.47_314.0905n | Crotoxyphos | C14H19O6P | 337.0797 | 2.46815 | Unclassified | 3.54454E-06 | 1.456829 |
| 5.00_159.0434m/z | 10-hydroxy-8E-Decene-2,4,6-triynoic acid | C10H8O3 | 159.0434 | 5.0003 | Fatty Acyls | 0.002786303 | 1.436815 |
| 0.82_298.0723m/z | S-NEPC | C16H13NO6 | 298.0723 | 0.819766667 | Unclassified | 0.001444245 | 1.427985 |
| 5.16_406.1027m/z | Flumetover | C19H20F3NO3 | 406.1027 | 5.157133333 | Unclassified | 0.001712964 | 1.42301 |
| 5.17_264.0856m/z | Coriandrone C | C13H10O5 | 264.0856 | 5.17245 | Isocoumarins and derivatives | 3.10115E-05 | 1.41583 |
| 2.02_213.0400m/z | 2-Hydroxy-6-ketononatrienedioate | C9H8O6 | 213.04 | 2.019183333 | Unclassified | 3.11261E-09 | 1.412338 |
| 0.88_203.1484m/z | Asymmetric dimethylarginine | C8H18N4O2 | 203.1484 | 0.8811 | Carboxylic acids and derivatives | 0.013000307 | 1.412335 |
| 13.18_340.3206m/z | 12Z,15Z-heneicosadienoic acid | C21H38O2 | 340.3206 | 13.18105 | Fatty Acyls | 0.025751101 | 1.409451 |
| 0.87_522.2010m/z | Levan | C18H32O16 | 522.201 | 0.865266667 | Organooxygen compounds | 0.042377662 | 1.408607 |
| 13.15_337.1662m/z | Propofol glucuronide | C18H26O7 | 337.1662 | 13.15105 | Organooxygen compounds | 0.006191582 | 1.3817 |
| 4.98_491.2572m/z | DIHYDROCELASTROL | C29H40O4 | 491.2572 | 4.984633333 | Unclassified | 0.0085514 | 1.36292 |
| 0.83_281.0733m/z | 5-Methylchrysene | C19H14 | 281.0733 | 0.834766667 | Unclassified | 0.002144456 | 1.329625 |
| 0.82_169.0654n | N-HFG | C7H8FN3O | 208.0421 | 0.819766667 | Unclassified | 0.001433619 | 1.321413 |
| 3.23_344.1708m/z | Citrusin C | C16H22O7 | 344.1708 | 3.228766667 | Organooxygen compounds | 0.044162735 | 1.314365 |
| 3.67_258.0898m/z | Ritalinic acid | C13H17NO2 | 258.0898 | 3.668233333 | Organonitrogen compounds | 0.019577377 | 1.293729 |
|  |  |  |  |  |  |  |  |
| 4.74_446.2384m/z | Taraxacolide 1-O-b-D-glucopyranoside | C21H32O9 | 446.2384 | 4.738816667 | Prenol lipids | 0.039967543 | 1.291475 |
| 13.26_393.3479m/z | Adrenoyl ethanolamide | C24H41NO2 | 393.3479 | 13.25705 | Organonitrogen compounds | 0.028011881 | 1.283062 |
| 7.25_222.0576m/z | S-Phenylmercapturic acid | C11H13NO3S | 222.0576 | 7.2508 | Carboxylic acids and derivatives | 0.038047007 | 1.26679 |
| 13.91_339.1818m/z | Pibutidine | C19H24N4O3 | 339.1818 | 13.91016667 | Organonitrogen compounds | 0.001125323 | 1.258879 |
| 5.77_312.2160m/z | Tanacetol A | C17H26O4 | 312.216 | 5.772083333 | Prenol lipids | 0.008107319 | 1.24511 |
| 5.69_475.1424m/z | 5alpha-Androstan-3alpha,17beta-diol disulfate | C19H32O8S2 | 475.1424 | 5.694916667 | Steroids and steroid derivatives | 0.008732477 | 1.24372 |
| 0.70_255.0618m/z | 5-Carboxy-2'-deoxyuridine | C10H12N2O7 | 255.0618 | 0.699616667 | Pyrimidine nucleosides | 0.000740474 | 1.241598 |
| 3.21_525.1777m/z | Ikarisoside D | C28H30O11 | 525.1777 | 3.214266667 | Polyketides | 4.26621E-05 | 1.24127 |
| 0.90_245.1366n | Valyl-Glutamine | C10H19N3O4 | 284.0966 | 0.896933333 | Carboxylic acids and derivatives | 0.037574292 | 1.240563 |
| 2.73_180.0648m/z | Adrenochrome | C9H9NO3 | 180.0648 | 2.7303 | Indoles and derivatives | 0.033400031 | 1.23295 |
| 11.56_319.1430m/z | MEFEXAMIDE | C15H24N2O3 | 319.143 | 11.555 | Unclassified | 0.023089483 | 1.224437 |
| 4.27_552.1827m/z | Aprepitant | C23H21F7N4O3 | 552.1827 | 4.265016667 | Oxazinanes | 0.000187019 | 1.21623 |
| 4.62_305.1091m/z | Vicine | C10H16N4O7 | 305.1091 | 4.618833333 | Organooxygen compounds | 0.007050856 | 1.213704 |
| 3.70_314.0762m/z | N-Succinyl-L-citrulline | C10H17N3O6 | 314.0762 | 3.6974 | Unclassified | 0.018401748 | 1.197549 |
| 4.74_344.1153m/z | Niazinin A | C15H21NO6S | 344.1153 | 4.738816667 | Organooxygen compounds | 4.68256E-05 | 1.196062 |
| 10.54_240.1717n | (1S,2S)-3-oxo-2-pentyl-cyclopentanebutanoic acid | C14H24O3 | 241.179 | 10.53823333 | Fatty Acyls | 0.011482755 | 1.191355 |
| 0.82_798.1652n | Apigenin 4'-[feruloyl-(->2)-glucuronyl-(1->2)-glucuronide] | C37H34O20 | 821.1544 | 0.819766667 | Flavonoids | 5.72028E-08 | 1.177065 |
| 12.83_334.2498n | (±)14(15)-EET methyl ester | C21H34O3 | 317.2465 | 12.83473333 | Unclassified | 0.044062545 | 1.176121 |
| 11.52_349.2341m/z | AVOCADYNE ACETATE | C19H34O4 | 349.2341 | 11.52433333 | Unclassified | 0.005159428 | 1.175335 |
| 0.73_219.0258m/z | 3-Deoxyarabinohexonic acid | C6H12O6 | 219.0258 | 0.731433333 | Hydroxy acids and derivatives | 0.038960693 | 1.168102 |
| 2.05_658.1619m/z | Nelumboside | C27H28O18 | 658.1619 | 2.049516667 | Flavonoids | 0.000207257 | 1.167347 |
| 12.52_335.1506m/z | Hydrocinchonine | C19H24N2O | 335.1506 | 12.51576667 | Cinchona alkaloids | 0.002979596 | 1.157666 |
| 5.16_827.1673m/z | 6-{4-[(1E)-3-{4,5-dihydroxy-3-[hydroxy(3,4,5-trihydroxyoxan-2-yl)methyl]-2,6-dioxo-5-[3,4,5-trihydroxy-6-(hydroxymethyl)oxan-2-yl]cyclohex-3-en-1-yl}-3-oxoprop-1-en-1-yl]phenoxy}-3,4,5-trihydroxyoxane-2-carboxylic acid | C33H40O22 | 827.1673 | 5.157133333 | Organooxygen compounds | 0.001157408 | 1.156349 |
| 4.91_372.1623m/z | Diphemanil Methylsulfate | C21H27NO4S | 372.1623 | 4.908466667 | Unclassified | 0.010945914 | 1.154194 |
| 11.41_392.1718m/z | Methysergide | C21H27N3O2 | 392.1718 | 11.40518333 | Ergoline and derivatives | 0.001767085 | 1.147748 |
| 2.02_678.1998m/z | 6-({3,7-dihydroxy-2-[3-hydroxy-10-(4-hydroxy-3-methoxyphenyl)-2-oxo-4-oxatricyclo[4.3.1.0³,⁷]decan-8-yl]-4-oxo-3,4-dihydro-2H-1-benzopyran-5-yl}oxy)-3,4,5-trihydroxyoxane-2-carboxylic acid | C31H32O16 | 678.1998 | 2.019183333 | Flavonoids | 1.90017E-07 | 1.144597 |
| 4.42_700.2568n | N-dodecanoyl-L-Homoserine lactone-3-hydrazone-fluorescein | C37H40N4O8S | 723.246 | 4.416183333 | Unclassified | 0.00142262 | 1.144592 |
| 5.08_462.2660m/z | desmethylastemizole | C27H29FN4O | 462.266 | 5.078633333 | Benzimidazoles | 3.94137E-05 | 1.144213 |
| 2.45_559.1670m/z | Acrinathrin | C26H21F6NO5 | 559.167 | 2.453483333 | Unclassified | 0.013901104 | 1.133873 |
| 2.45_272.1017m/z | Lysyl-Serine | C9H19N3O4 | 272.1017 | 2.453483333 | Carboxylic acids and derivatives | 0.004067691 | 1.128692 |
| 3.11_313.1306m/z | Pseudomonine | C16H18N4O4 | 313.1306 | 3.107433333 | Carboxylic acids and derivatives | 0.003281647 | 1.121648 |
| 11.45_382.2710n | ent-7-F2t-dihomo-IsoP | C22H38O5 | 400.3048 | 11.44966667 | Fatty Acyls | 0.030300158 | 1.114271 |
| 15.58_395.3632m/z | 5alpha-Cholestane | C27H48 | 395.3632 | 15.57688333 | Steroids and steroid derivatives | 0.019975273 | 1.110552 |
| 0.73_175.1178m/z | L-Arginine | C6H14N4O2 | 175.1178 | 0.731433333 | Carboxylic acids and derivatives | 0.03509792 | 1.101649 |
| 5.34_476.2837m/z | [6]-Gingerdiol 5-O-beta-D-glucopyranoside | C23H38O9 | 476.2837 | 5.33995 | Fatty Acyls | 1.44349E-05 | 1.095511 |
| 3.19_392.0846m/z | Boc-Asn-OPhNO2 | C15H19N3O7 | 392.0846 | 3.185266667 | Unclassified | 0.008794168 | 1.09386 |
| 0.87_580.0459m/z | Pentafluorobenzenesulfonyl fluorescein | C26H11F5O7S | 580.0459 | 0.865266667 | Unclassified | 0.007190893 | 1.089749 |
| 11.97_572.3914n | IC202A | C27H52N6O7 | 590.4252 | 11.9678 | Fatty Acyls | 0.001008671 | 1.089536 |
| 5.74_220.0959m/z | 3-Hydroxy-carbofuran | C12H15NO4 | 220.0959 | 5.74125 | Coumarans | 0.016208399 | 1.089303 |
| 6.42_174.0543m/z | 5-Phenyl-1,3-oxazinane-2,4-dione | C10H9NO3 | 174.0543 | 6.4232 | Benzene and substituted derivatives | 0.005727355 | 1.085775 |
| 5.16_811.1954m/z | Cyanidin 3-O-[2''-O-(xylosyl) glucoside] 5-O-(6'''-O-malonyl) glucoside | C35H40O23 | 811.1954 | 5.157133333 | Unclassified | 0.000136171 | 1.08548 |
| 3.21_548.2538m/z | N6'-Acetylkanamycin-B | C20H39N5O11 | 548.2538 | 3.214266667 | Unclassified | 1.05542E-05 | 1.084816 |
| 0.85_659.1019m/z | 6-{[6-(3,4-dihydroxy-6-methyl-5-oxooxan-2-yl)-5-hydroxy-2-(4-hydroxy-3-methoxyphenyl)-4-oxo-4H-chromen-7-yl]oxy}-3,4,5-trihydroxyoxane-2-carboxylic acid | C28H28O16 | 659.1019 | 0.849933333 | Flavonoids | 0.000315633 | 1.067201 |
| 4.49_258.1692m/z | Hydroxyhexanoycarnitine | C13H25NO5 | 258.1692 | 4.494333333 | Fatty Acyls | 0.021899167 | 1.063738 |
| 13.26_393.2968m/z | Dioctyl hexanedioate | C22H42O4 | 393.2968 | 13.25705 | Fatty Acyls | 0.03936139 | 1.04715 |
| 12.04_324.2290n | methyl 15-hydroperoxy-9Z,12Z,16E-octadecatrienoate | C19H32O4 | 325.2363 | 12.04363333 | Fatty Acyls | 0.012825789 | 1.035493 |
| 5.51_356.1276n | Brosimacutin G | C20H20O6 | 357.1323 | 5.510766667 | Polyketides | 0.007781198 | 1.033191 |
| 10.63_342.2990m/z | Cer(d18:1/2:0) | C20H39NO3 | 342.299 | 10.62923333 | Sphingolipids | 0.01272129 | 1.029698 |
| 5.85_146.0591m/z | 1H-Indole-3-carboxaldehyde | C9H7NO | 146.0591 | 5.84875 | Indoles and derivatives | 0.011107126 | 1.017605 |
| 4.37_194.0570n | trans-Ferulic acid | C10H10O4 | 177.0537 | 4.369516667 | Cinnamic acids and derivatives | 0.021797648 | 1.013321 |
| 4.46_450.2111m/z | Melledonal A | C23H28O8 | 450.2111 | 4.463016667 | Prenol lipids | 0.042410528 | 1.010705 |
| 2.02_267.0577m/z | CAY10512 | C15H13FO | 267.0577 | 2.019183333 | Unclassified | 0.028557946 | 1.007097 |
| 13.74_395.2193m/z | Neriantogenin | C23H32O4 | 395.2193 | 13.74285 | Steroids and steroid derivatives | 0.049994759 | 1.004635 |
| 4.91_286.1283m/z | Acarbose (component 1) | C13H21NO7 | 286.1283 | 4.908466667 | Unclassified | 0.00587596 | 1.000234 |

Table S7 The negative ion modes of metabolites between T1 and T2

| **ID** | **Metabolites** | **Formula** | **m/z** | **Retention time (min)** | **Class** | **P-value** | **VIP** |
| --- | --- | --- | --- | --- | --- | --- | --- |
| 4.68_121.0303m/z | 4-Hydroxybenzaldehyde | C7H6O2 | 121.03 | 4.681733333 | Organooxygen compounds | 9.3E-08 | 12.6601 |
| 5.16_340.1100n | Diphenadione | C23H16O3 | 385.116 | 5.161866667 | Unclassified | 7.7E-05 | 11.2717 |
| 3.72_294.0950n | 6-O-(3R,4-dihydroxy-2-methylene-butanoyl)-beta-D-glucopyranose | C11H18O9 | 587.183 | 3.715466667 | Unclassified | 0.03759 | 9.96905 |
| 11.41_294.2205n | 13S-HOTrE(gamma) | C18H30O3 | 293.2133 | 11.40625 | Fatty Acyls | 0.001673579 | 6.995179 |
| 0.89_134.0218n | L-Malic acid | C4H6O5 | 133.0153 | 0.894833333 | Hydroxy acids and derivatives | 0.025516365 | 6.963853 |
| 2.05_208.0947n | Ethyl beta-D-glucopyranoside | C8H16O6 | 253.0931 | 2.046916667 | Organooxygen compounds | 1.37788E-05 | 6.428179 |
| 4.67_353.0276m/z | Fensulfothion | C11H17O4PS2 | 353.0276 | 4.669233333 | Unclassified | 9.86611E-09 | 5.821931 |
| 0.84_439.0761m/z | Thidiazuron | C9H8N4OS | 439.0761 | 0.843833333 | Unclassified | 1.79178E-08 | 5.666185 |
| 2.49_252.1206n | 2,3-Butanediol glucoside | C10H20O7 | 297.1195 | 2.48505 | Organooxygen compounds | 0.000999295 | 5.123279 |
| 0.77_215.0333m/z | 2-C-Methyl-D-erythritol 4-phosphate | C5H13O7P | 215.0333 | 0.768683333 | Unclassified | 0.006661575 | 4.684705 |
| 4.95_386.1216n | 6-{[5-(3,4-dihydroxyphenyl)pentanoyl]oxy}-3,4,5-trihydroxyoxane-2-carboxylic acid | C17H22O10 | 385.1144 | 4.946716667 | Organooxygen compounds | 0.000220856 | 4.592378 |
| 3.72_250.1056n | Isopropyl citrate | C10H18O7 | 249.0983 | 3.715466667 | Unclassified | 0.020304927 | 4.042664 |
| 0.84_342.1151n | D-Maltose | C12H22O11 | 387.1133 | 0.843833333 | Organooxygen compounds | 8.53508E-05 | 4.023276 |
| 4.90_181.0152m/z | 4-fumarylacetoacetic acid | C8H8O6 | 181.0152 | 4.89605 | Keto acids and derivatives | 1.56229E-05 | 3.646487 |
| 5.74_220.0986m/z | Metaxalone | C12H15NO3 | 220.0986 | 5.741483333 | Phenol ethers | 0.01206889 | 3.466098 |
| 3.72_207.0880m/z | 4-hydroxy-butyric acid | C4H8O3 | 207.088 | 3.715466667 | Fatty Acyls | 0.008527525 | 3.191645 |
| 5.02_347.1342m/z | Garcinia lactone dibutyl ester | C14H22O7 | 347.1342 | 5.022533333 | Carboxylic acids and derivatives | 0.010088072 | 2.784705 |
| 4.92_433.2080m/z | 5a,6a-Epoxy-7E-megastigmene-3a,9e-diol 3-glucoside | C19H32O8 | 433.208 | 4.921383333 | Organooxygen compounds | 0.017265499 | 2.770417 |
| 0.87_638.1875n | 7-O-Methyltectorigenin 4'-O-gentiobioside | C29H34O16 | 637.1824 | 0.869166667 | Polyketides | 0.008580391 | 2.733195 |
| 0.86_263.0646n | Ascorbalamic acid | C9H13NO8 | 262.0573 | 0.8565 | Carboxylic acids and derivatives | 0.047689116 | 2.7328 |
| 0.81_179.0560m/z | Alpha-D-Glucopyranoside | C6H12O6 | 179.056 | 0.806683333 | Organooxygen compounds | 0.013979178 | 2.718552 |
| 4.91_581.1869m/z | 10-Acetoxyligustroside | C27H34O14 | 581.1869 | 4.908716667 | Prenol lipids | 0.002006323 | 2.662558 |
| 0.83_135.0302m/z | Threonic acid | C4H8O5 | 135.0302 | 0.8315 | Organooxygen compounds | 0.033362639 | 2.576096 |
| 3.72_609.1646m/z | Convicine | C10H15N3O8 | 609.1646 | 3.715466667 | Unclassified | 0.019072391 | 2.487572 |
| 0.79_129.0193m/z | 2,5-Dioxopentanoate | C5H6O4 | 129.0193 | 0.794183333 | Keto acids and derivatives | 0.043765082 | 2.336138 |
| 0.95_289.0176m/z | cis-Resveratrol 4'-sulfate | C14H12O6S | 289.0176 | 0.945333333 | Stilbenes | 0.007149847 | 2.267242 |
| 5.14_264.0875m/z | N-Phenylacetylglutamic acid | C13H15NO5 | 264.0875 | 5.136366667 | Carboxylic acids and derivatives | 0.004172241 | 2.104275 |
| 11.00_291.1968m/z | 9S-HpOTrE | C18H30O4 | 291.1968 | 10.99978333 | Fatty Acyls | 0.00884996 | 2.071223 |
| 6.96_268.0979m/z | Pipermethystine | C16H17NO4 | 268.0979 | 6.960566667 | Pyridines and derivatives | 0.000140389 | 2.043 |
| 5.88_441.1425m/z | Methyl salicylate O-[rhamnosyl-(1->6)-glucoside] | C20H28O12 | 441.1425 | 5.88015 | Organooxygen compounds | 0.016470538 | 1.992756 |
| 11.36_297.2439m/z | 8Z-decen-4,6-diynoic acid | C18H34O3 | 297.2439 | 11.36408333 | Fatty Acyls | 4.35238E-05 | 1.991886 |
| 2.11_103.0403m/z | 3-Hydroxybutyric acid | C4H8O3 | 103.0403 | 2.11025 | Hydroxy acids and derivatives | 0.000118904 | 1.937778 |
| 0.81_165.0406m/z | L-Lyxonate | C5H10O6 | 165.0406 | 0.806683333 | Organooxygen compounds | 0.001539345 | 1.921586 |
| 10.76_559.3121m/z | 1-NBD-decanoyl-2-decanoyl-sn-Glycerol | C29H46N4O8 | 559.3121 | 10.75746667 | Unclassified | 0.021415855 | 1.921414 |
| 3.77_338.0882m/z | Cyclodopa glucoside | C15H19NO9 | 338.0882 | 3.765466667 | Organooxygen compounds | 0.01592629 | 1.880199 |
| 0.82_237.0613m/z | 3,4,5-trihydroxy-6-(2-hydroxyethoxy)oxane-2-carboxylic acid | C8H14O8 | 237.0613 | 0.819183333 | Organooxygen compounds | 0.029042251 | 1.813856 |
| 0.79_399.0940m/z | Biflorin | C16H18O9 | 399.094 | 0.794183333 | Organooxygen compounds | 0.02982155 | 1.789244 |
| 13.14_279.2336m/z | Linoleic acid | C18H32O2 | 279.2336 | 13.14128333 | Fatty Acyls | 0.000169998 | 1.775888 |
| 5.43_221.0279m/z | S-(2,5-Dimethyl-3-furanyl) 2-furancarbothioate | C11H10O3S | 221.0279 | 5.4265 | Furans | 0.003699938 | 1.741141 |
| 6.52_303.0534m/z | 5-(3-methoxyphenyl)-4-(sulfooxy)pentanoic acid | C12H16O7S | 303.0534 | 6.520433333 | Fatty Acyls | 1.53141E-05 | 1.715287 |
| 0.87_601.1281m/z | 1-Guanidino-1-deoxy-scyllo-inositol 4-phosphate | C7H16N3O8P | 601.1281 | 0.869166667 | Unclassified | 0.026959375 | 1.70745 |
| 8.99_307.1911m/z | 16-epi-16-D1t-PhytoP | C18H30O5 | 307.1911 | 8.993083333 | Fatty Acyls | 0.014007486 | 1.700479 |
| 11.08_295.2283m/z | (±)9-HODE | C18H32O3 | 295.2283 | 11.07626667 | Unclassified | 0.042752889 | 1.691594 |
| 4.51_339.1310m/z | 2,3,4-Trihydroxybenzylhydrazide | C7H10N2O3 | 339.131 | 4.505233333 | Unclassified | 0.012879727 | 1.667352 |
| 5.19_636.1908m/z | Morphine 3,6-diglucuronide | C29H35NO15 | 636.1908 | 5.186866667 | Unclassified | 0.006638502 | 1.640478 |
| 2.08_311.1339m/z | Olanzapine | C17H20N4S | 311.1339 | 2.084916667 | Benzodiazepines | 5.25701E-06 | 1.626432 |
| 11.58_295.2281m/z | 12-Hydroxy-8,10-octadecadienoic acid | C18H32O3 | 295.2281 | 11.57573333 | Fatty Acyls | 0.005620905 | 1.598719 |
| 4.92_394.1711m/z | Latrunculin B | C20H29NO5S | 394.1711 | 4.921383333 | Unclassified | 0.010948208 | 1.596619 |
| 4.27_351.1288m/z | (±)-Glycerol 1,2-diacetate | C7H12O5 | 351.1288 | 4.26575 | Glycerolipids | 0.000188254 | 1.582379 |
| 2.19_432.1341m/z | Apholate | C12H24N9P3 | 432.1341 | 2.185566667 | Unclassified | 0.010672624 | 1.580864 |
| 5.85_144.0461m/z | 4-Oxo-1-(3-pyridyl)-1-butanone | C9H9NO2 | 144.0461 | 5.854983333 | Organooxygen compounds | 0.032070053 | 1.560797 |
| 4.37_356.1099n | (3,4,5,6-tetrahydroxyoxan-2-yl)methyl 3-(4-hydroxy-3-methoxyphenyl)prop-2-enoate | C16H20O9 | 355.1026 | 4.366916667 | Cinnamic acids and derivatives | 0.011021726 | 1.516383 |
| 0.86_609.1866m/z | Pirimiphos-methyl | C11H20N3O3PS | 609.1866 | 0.8565 | Unclassified | 0.000888608 | 1.514915 |
| 5.16_793.2168m/z | Rhamnetin 3-rhamnosyl-(1->3)(4'''-acetylrhamnosyl)(1->6)-galactoside | C36H44O21 | 793.2168 | 5.161866667 | Polyketides | 0.003642939 | 1.457998 |
| 2.92_300.0846n | (3,4,5,6-tetrahydroxyoxan-2-yl)methyl 4-hydroxybenzoate | C13H16O8 | 299.0773 | 2.91585 | Benzene and substituted derivatives | 0.024626512 | 1.436618 |
| 2.03_504.0669m/z | 4-Deoxy-beta-D-gluc-4-enuronosyl-(1,3)-N-acetyl-D-galactosamine 4-sulfate | C14H21NO14S | 504.0669 | 2.034416667 | Unclassified | 3.26307E-06 | 1.393938 |
| 0.88_643.1695m/z | [1-(4-methoxyphenyl)-3-phenylpropoxy]sulfonic acid | C16H18O5S | 643.1695 | 0.882166667 | Linear 1,3-diarylpropanoids | 0.021923374 | 1.390441 |
| 6.96_224.1081m/z | Agomelatine | C15H17NO2 | 224.1081 | 6.960566667 | Carboxylic acids and derivatives | 2.05018E-05 | 1.367418 |
| 5.09_399.1285m/z | Methyl helianthenoate F glucoside | C17H22O8 | 399.1285 | 5.085866667 | Fatty Acyls | 0.013250932 | 1.329669 |
| 2.49_351.0377m/z | 4-hydroxy-5-[4-hydroxy-3-(sulfooxy)phenyl]pentanoic acid | C11H14O8S | 351.0377 | 2.48505 | Fatty Acyls | 4.92193E-06 | 1.319551 |
| 4.74_509.1859m/z | 7,8-Dihydroneopterin | C9H13N5O4 | 509.1859 | 4.744383333 | Pteridines and derivatives | 0.028678469 | 1.309579 |
| 4.74_509.1859m/z | 7,8-Dihydroneopterin | C9H13N5O4 | 509.1859 | 4.744383333 | Pteridines and derivatives | 0.028678469 | 1.309579 |
| 0.84_379.0822m/z | 19-Hydroxy-8-O-methyltetrangulol | C20H14O5 | 379.0822 | 0.843833333 | Unclassified | 0.01846726 | 1.272608 |
| 1.68_188.0566m/z | 5-ethyl-5-methyl-2,4-oxazolidinedione | C6H9NO3 | 188.0566 | 1.676116667 | Azolidines | 0.043708002 | 1.264029 |
| 0.84_368.0578n | {6-hydroxy-3-[3-(3-hydroxyphenyl)-3-oxopropyl]-2-methoxyphenyl}oxidanesulfonic acid | C16H16O8S | 367.0505 | 0.843833333 | Linear 1,3-diarylpropanoids | 0.021898417 | 1.244595 |
| 3.32_249.0982m/z | Pentostatin | C11H16N4O4 | 249.0982 | 3.319183333 | Imidazodiazepines | 0.001631902 | 1.22706 |
| 13.93_281.2488m/z | Elaidic acid | C18H34O2 | 281.2488 | 13.9304 | Fatty Acyls | 0.021564983 | 1.217842 |
| 0.68_164.9351m/z | Magnesium Sulfate | MgO4S | 164.9351 | 0.67835 | Unclassified | 0.008574191 | 1.205152 |
| 3.72_262.0144n | 3-hydroxy-3-(3-hydroxyphenyl)propanoic acid-O-sulphate | C9H10O7S | 307.0113 | 3.715466667 | Hydroxy acids and derivatives | 0.007476506 | 1.189662 |
| 0.88_337.0767m/z | 3,4,5-trihydroxy-6-[(4-hydroxy-3-methylbut-2-enoyl)oxy]oxane-2-carboxylic acid | C11H16O9 | 337.0767 | 0.882166667 | Organooxygen compounds | 0.029813912 | 1.178312 |
| 7.48_451.1960m/z | phorbol 13-acetate | C22H30O7 | 451.196 | 7.4839 | Prenol lipids | 0.004642044 | 1.176485 |
| 4.69_327.1100m/z | Ricinine | C8H8N2O2 | 327.11 | 4.6944 | Pyridines and derivatives | 0.000106959 | 1.176186 |
| 0.84_152.9866m/z | 2,4,6-Trithiaheptane | C4H10S3 | 152.9866 | 0.843833333 | Thioacetals | 0.025611745 | 1.171058 |
| 5.01_207.0122m/z | 1-Naphthalenesulfonic acid | C10H8O3S | 207.0122 | 5.009866667 | Unclassified | 0.005399079 | 1.168408 |
| 5.55_141.0923m/z | 2-Octenoic acid | C8H14O2 | 141.0923 | 5.552333333 | Fatty Acyls | 0.003811481 | 1.15976 |
| 2.08_364.0448m/z | Fludarabine phosphate | C10H13FN5O7P | 364.0448 | 2.084916667 | Purine nucleotides | 1.19519E-05 | 1.153464 |
| 4.68_377.1803m/z | (1S,2R,4R)-p-Menth-8-ene-2,10-diol 2-glucoside | C16H28O7 | 377.1803 | 4.681733333 | Prenol lipids | 5.51269E-05 | 1.152866 |
| 4.71_421.1644m/z | Sophoraflavanone D | C25H28O7 | 421.1644 | 4.7069 | Polyketides | 0.006205278 | 1.129664 |
| 2.21_162.0529n | 2-Hydroxy-2-ethylsuccinic acid | C6H10O5 | 161.0459 | 2.210566667 | Fatty Acyls | 0.044194987 | 1.129474 |
| 4.74_243.1716m/z | Leucyl-Isoleucine | C12H24N2O3 | 243.1716 | 4.744383333 | Carboxylic acids and derivatives | 0.037018763 | 1.129005 |
| 2.07_130.0875m/z | L-Leucine | C6H13NO2 | 130.0875 | 2.07225 | Carboxylic acids and derivatives | 0.015220051 | 1.12383 |
| 10.80_375.1661m/z | Schizonepetoside B | C16H26O7 | 375.1661 | 10.79578333 | Unclassified | 0.009423083 | 1.114144 |
| 5.74_178.0877m/z | 1,2,3,4-Tetrahydroisoquinoline | C9H11N | 178.0877 | 5.741483333 | Tetrahydroisoquinolines | 0.023817383 | 1.111824 |
| 5.65_377.0903m/z | Thiotepa | C6H12N3PS | 377.0903 | 5.653333333 | Organic thiophosphoric acids and derivatives | 0.035289414 | 1.11 |
| 2.05_307.0115m/z | 1-(2-Thienyl)-1,2-propanedione | C7H6O2S | 307.0115 | 2.046916667 | Organooxygen compounds | 5.77435E-07 | 1.08815 |
| 5.16_453.0996m/z | Nicorandil-N-oxide | C8H9N3O5 | 453.0996 | 5.161866667 | Unclassified | 2.82859E-06 | 1.075962 |
| 1.47_133.0503m/z | 1-Deoxy-D-xylulose | C5H10O4 | 133.0503 | 1.473783333 | Organooxygen compounds | 7.13546E-06 | 1.065558 |
| 5.12_207.0121m/z | 2-Naphthalenesulfonic acid | C10H8O3S | 207.0121 | 5.123866667 | Unclassified | 0.000326115 | 1.061958 |
| 10.72_293.2117m/z | 9(S)-HOTrE | C18H30O3 | 293.2117 | 10.7153 | Unclassified | 0.028904057 | 1.059512 |
| 7.20_531.1525m/z | 6-(5-{2-[4,6-dihydroxy-2-methoxy-3-(3-methylbut-2-en-1-yl)phenyl]acetyl}-2,4-dihydroxyphenoxy)-3,4,5-trihydroxyoxane-2-carboxylic acid | C26H30O13 | 531.1525 | 7.195883333 | Stilbenes | 0.023323026 | 1.051468 |
| 9.66_309.2066m/z | 13(S)-HpOTrE | C18H30O4 | 309.2066 | 9.656883333 | Unclassified | 0.033054469 | 1.045647 |
| 12.52_277.2176m/z | 9(10)-EpOME | C18H32O3 | 277.2176 | 12.51816667 | Fatty Acyls | 0.003970357 | 1.038422 |
| 0.93_103.0035m/z | Tartronate semialdehyde | C3H4O4 | 103.0035 | 0.932666667 | Organooxygen compounds | 0.0065572 | 1.035639 |
| 4.13_353.0988m/z | Indicaxanthin | C14H16N2O6 | 353.0988 | 4.127766667 | Carboxylic acids and derivatives | 0.032387835 | 1.028282 |
| 1.21_643.1714m/z | Isosorbide-2-glucuronide | C12H18O10 | 643.1714 | 1.212816667 | Unclassified | 0.025798764 | 1.018758 |
| 4.87_295.1394m/z | 2,3-Dihydroxy-3-methylpentanoic acid | C6H12O4 | 295.1394 | 4.870883333 | Fatty Acyls | 0.039743251 | 1.006972 |
| 2.27_223.0283m/z | 3,3'-Thiobispropanoic acid | C6H10O4S | 223.0283 | 2.273066667 | Carboxylic acids and derivatives | 0.004256848 | 1.006593 |
